# DMAP-Promoted Cascade Synthesis of Bispirooxindole–Cyclopentene–Isoxazolones

**DOI:** 10.3390/molecules31142461

**Published:** 2026-07-14

**Authors:** Wei Zhang, Rong-Rong Zhu, Da-Ming Du

**Affiliations:** School of Chemistry and Chemical Engineering, Beijing Institute of Technology, Beijing 100081, China; 3220231751@bit.edu.cn (W.Z.);

**Keywords:** Michael addition/cyclization, isoxazol-5-one, spirooxindole, DMAP catalysis

## Abstract

A DMAP-catalyzed Michael addition/cyclization cascade reaction has been developed for the efficient construction of a bispirooxindole–cyclopentene–isoxazolone framework. Under the optimized conditions (10 mol% DMAP, CH_2_Cl_2_, room temperature), the desired product was obtained in 92% yield with >20:1 diastereoselectivity. The reaction exhibited broad substrate scope, tolerating various substituents on both the isoxazolone and oxindole rings, and affording the corresponding products in 72–96% yields. The relative configuration of a representative product was determined as rel-(2’*S*,3*R*,3’*R*) by X-ray single-crystal diffraction. The utility of this method was further demonstrated by a gram-scale experiment (82% yield). This cascade reaction provides a concise and efficient route to structurally novel spiroisoxazolone derivatives bearing a cyano group and multiple spiro chiral centers.

## 1. Introduction

**Figure 1:**
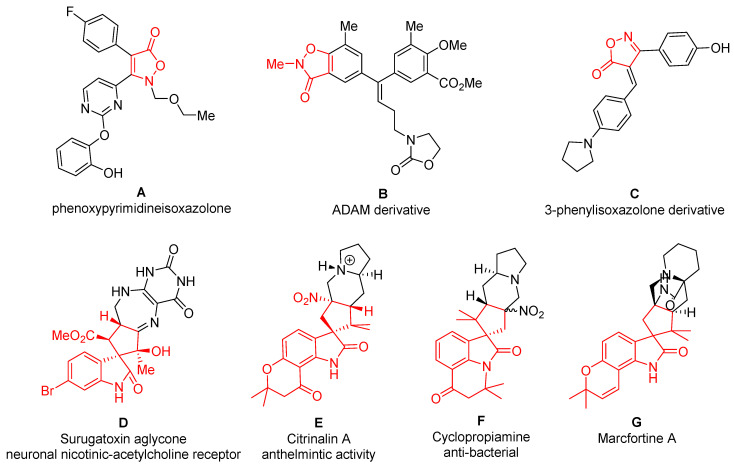
Examples of biologically active isoxazolone and spirocyclic compounds.

Isoxazolones are an important class of five-membered nitrogen- and oxygen-containing heterocyclic compounds widely found in natural products, pharmaceuticals, and agrochemicals. This heterocyclic system exhibits diverse pharmacological properties in clinical therapy, particularly in antitumor, antimicrobial (including fungi, viruses, and Mycobacterium tuberculosis), and anti-inflammatory applications [1,2,3,4,5,6]. For example, phenoxypyrimidine–isoxazolone Figure 1A can be used as a tumor necrosis factor-α (TNF-α) inhibitor [7]; the structurally modified isoxazolone–alkenyldiarylmethane hybrid Figure 1B retains anti-HIV activity while its enhanced metabolic stability effectively reduces systemic toxicity [8]; and (*Z*)-4-(4-pyrrolidinylbenzylidene)-3-phenyl-5(4*H*)-isoxazolone Figure 1C has been successfully employed as a potent androgen antagonist, displaying significant inhibitory effects on LNCaP cells expressing a point-mutated, constitutively active androgen receptor, with low side effects(Figure 1) [9].

Spirocyclic compounds are abundant in natural products and drug molecules [10,11,12], characterized by a polycyclic system connected through a spiro quaternary carbon. This topological feature endows them with significant stereochemical advantages: the spatial interactions between adjacent stereocenters impart a rigid conformation and a complex three-dimensional structure that distinguishes spirocycles from ordinary monocyclic compounds or planar systems. This can induce special electronic effects (e.g., inter-ring conjugation), thereby effectively modulating pharmacokinetic properties and enhancing drug potency and target selectivity when used as pharmaceutically active scaffolds [13,14,15,16,17,18,19,20]. Compounds featuring a multi-spirocyclopentane–oxindole framework represent a highly representative class of such structures. For instance, Surugatoxin aglycone Figure 1D exhibits nanomolar inhibitory activity against neuronal nicotinic acetylcholine receptors; the alkaloids citrinadin Figure 1E, hydroxyquinamide Figure 1F, and marcfortine Figure 1G show pronounced anthelmintic and antibacterial activities (Figure 1). Meanwhile, malononitrile derivatives have attracted considerable attention because their cyano groups can be efficiently converted into various functional groups such as carboxylic acids, amides, and heterocycles [21,22].

**Scheme 1:**
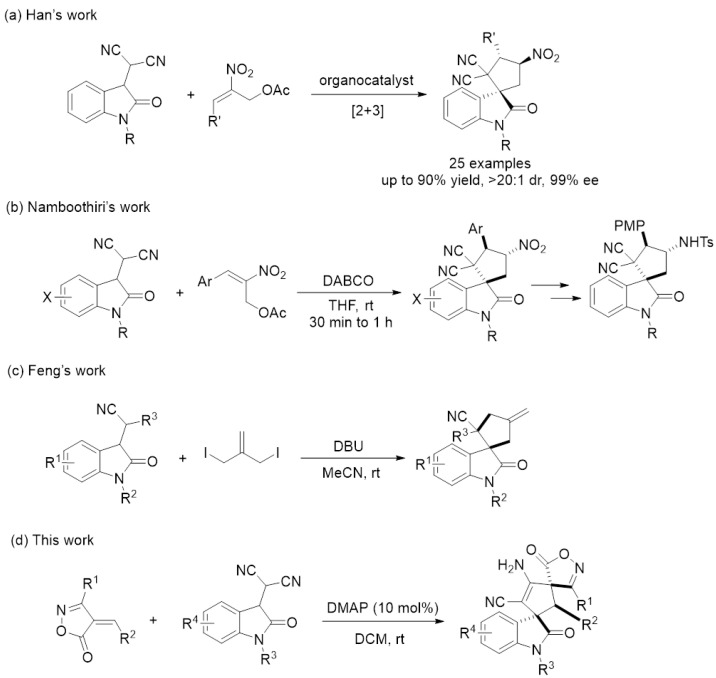
Studies on the transformation of 3-(2-oxoindolin-3-yl)malononitriles and our work [25,26,27].

To date, there have been reports on the construction of cyano-containing spirooxindoles using malononitrile derivatives [23,24]. Han and co-workers [25] employed 2-(2-oxoindolin-3-yl)malononitrile as 1,2-bisnucleophiles in an asymmetric catalytic [3 + 2] cycloaddition to efficiently build highly enantioselective spirocyclopentane–oxindoles. Similarly, Namboothiri [26] utilized a [3 + 2] cycloaddition strategy to achieve the highly stereoselective annulation of 2-(2-oxoindolin-3-yl)malononitrile with MBH acetates of nitroalkenes, providing a new approach to multi-stereocenter spirocyclic compounds. Feng and co-workers [27] reported a base-mediated [2 + 3] annulation of 2-(2-oxoindolin-3-yl)malononitrile with 3-iodo-2-iodomethylprop-1-ene to access methylenecyclopentane spirooxindoles (Scheme 1).

However, these efforts have mostly focused on the construction of spirocyclopentane or spiroquinoline frameworks, and efficient methods for the synthesis of bispirocyclic systems remain scarce, especially those involving malononitrile units as 1,3-bisnucleophiles in cascade cyclizations. Based on the above research status, the present study aims to develop an organocatalytic strategy to promote a Michael addition/cyclization cascade reaction between 4-benzylidene unsaturated isoxazol-5-ones and 2-(2-oxoindolin-3-yl)malononitriles, with the goal of accessing a novel class of cyano-containing bispirooxindole–cyclopentene–isoxazolone derivatives.

## 2. Results and Discussion

### 2.1. Optimization of Reaction Conditions

In the initial experimental stage, the Michael addition/cyclization cascade reaction of (*Z*)-4-benzylidene-3-methylisoxazol-5(4*H*)-one (**1a**) with 2-(1-methyl-2-oxoindolin-3-yl)malononitrile (**2a**) was attempted. Using 10 mol% triethylamine (Et3N) as the base in CH_2_Cl_2_ at room temperature for 10 h, the corresponding bispiro(isoxazolone-cyclopentenyl)oxindole **3aa** was obtained in 58% yield with excellent diastereoselectivity. Although the yield was moderate, this result preliminarily validated the feasibility of the reaction pathway, suggesting that further optimization could lead to improved efficiency. Consequently, subsequent screenings of catalyst type, catalyst loading, reaction temperature, and solvent were carried out using this model reaction.

First, the effect of the solvent was evaluated. When the protic solvent ethanol was used, the cascade reaction did not proceed as efficiently; the yield decreased slightly to 53% compared to that obtained with CH_2_Cl_2_. A series of aprotic solvents was then tested. Tetrahydrofuran, which has a polarity comparable to that of CH_2_Cl_2_, gave a yield of 55%, showing only a minor fluctuation. Using chloroform or the non-polar solvent toluene afforded **3aa** in yields of 56% and 57%, respectively. In contrast, when the strongly polar solvent acetonitrile was employed, the yield dropped significantly to 45%. Notably, excellent diastereoselectivity was maintained throughout all these solvent screenings.

Subsequently, a screening of catalysts was performed using CH_2_Cl_2_ as the solvent. When the organic bases 4-(dimethylamino)pyridine (DMAP), N,N-diisopropylethylamine (DIPEA), and 1,8-diazabicyclo[5.4.0]undec-7-ene (DBU) were employed, DMAP gave the highest yield of the cascade cyclization product (90%), while DIPEA and DBU afforded **3aa** in 76% and 55% yields, respectively. The promoting effect of several inorganic bases was also examined. NaHCO_3_ gave a yield comparable to that obtained with Et_3_N (57%), whereas the significantly stronger base Na_2_CO_3_ increased the yield to 87%. The influence of K_2_CO_3_ and Cs_2_CO_3_ was also evaluated; K_2_CO_3_ resulted in only a 43% yield, while Cs_2_CO_3_ maintained the yield at 63%. To gain a comprehensive understanding of the reaction between benzylidene unsaturated isoxazol-5-one **1a** and 2-(1-methyl-2-oxoindolin-3-yl)malononitrile **2a**, a control experiment was conducted in the absence of any catalyst (Table 1, entry 14). The results showed that the reaction still proceeded, indicating that the chosen substrates possess excellent inherent reactivity.

Elevating the temperature had a negative impact, likely due to the occurrence of side reactions, reducing the yield to 45%. When the temperature was raised to 50 °C, it was detrimental, giving only a 45% yield (Table 1, entry 15), whereas lowering the temperature to −10 °C maintained a high yield of 78% (Table 1, entry 16). In addition, the catalyst loading was optimized. When 20 mol% of DMAP was used, the product yield dropped to 57%; reducing the loading to 5 mol% still gave a yield of 77%. Therefore, 10 mol% DMAP was selected. Using CH_2_Cl_2_ as the solvent with 10 mol% DMAP, the effect of reaction temperature was further investigated. Throughout the entire optimization process, the diastereoselectivity (dr) consistently remained excellent (>20:1), which may be attributed to the inherent structural rigidity of the substrates or the kinetic advantage of the Michael addition step. For cases where dr is reported as >20:1, the minor diastereomer was not detectable by ^1^H NMR analysis of the crude reaction mixture.

Based on the above results, the optimal reaction conditions were established as follows: 10 mol% DMAP as the catalyst, CH_2_Cl_2_ as the solvent, and the reaction carried out at room temperature. Subsequently, the substrate scope was investigated under these optimized conditions.

### 2.2. Substrate Scope

**Scheme 2:**
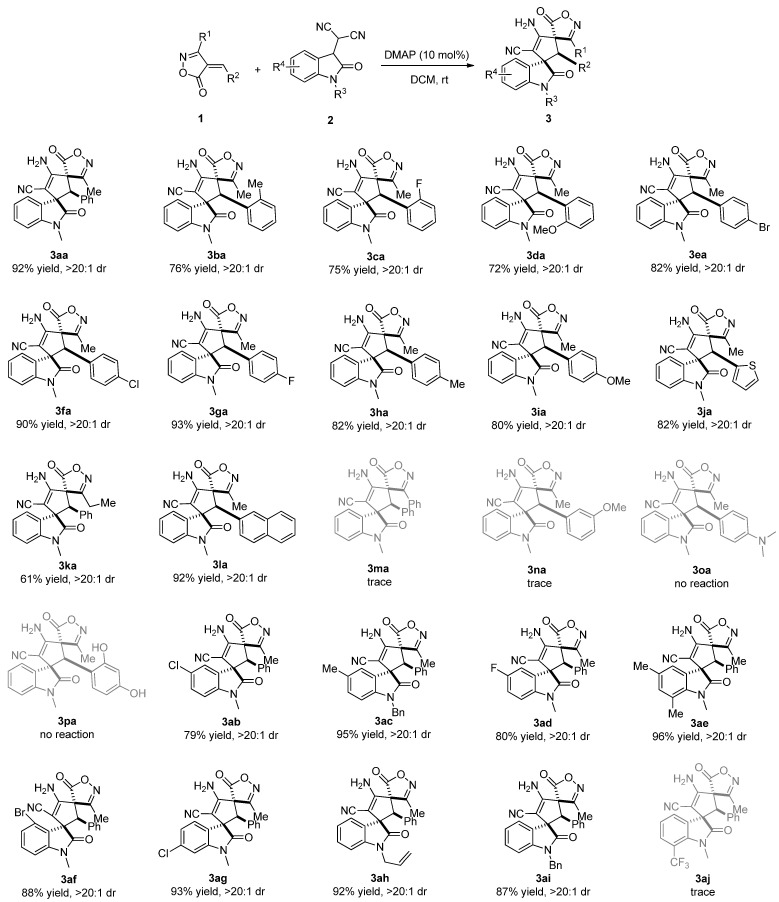
Unless otherwise specified, the reaction conditions were as follows: **1** (0.15 mmol), **2** (0.20 mmol), and DMAP (10 mol%) in 1.0 mL of CH_2_Cl_2_, stirred at room temperature for 12 h. Yields are isolated yields after column chromatography, and dr values were determined by ^1^H NMR analysis.

Based on the established optimal reaction conditions, the influence of the spatial distribution and electronic properties of substituents on the aromatic ring of the unsaturated isoxazol-5-ones, as well as the position and electronic nature of substituents on 2-(2-oxoindolin-3-yl)malononitriles, was systematically investigated. A series of structurally novel bispirooxindole–cyclopentene–isoxazol-5-one heterocyclic compounds were synthesized (Scheme 2).

Experimental data showed that under the standard conditions, the target product **3aa** was obtained in a 92% isolated yield in the absence of substituents. First, the effect of different substituents on the aromatic ring at the 4-position of the isoxazol-5-one was examined. When a substituent was present at the *ortho* position of the aromatic ring, regardless of whether it was electron-withdrawing or electron-donating, a significant decrease in yield was observed. For methyl or methoxy substituents, products **3ba** and **3da** were obtained in 76% and 72% yield, respectively; a fluorine substituent gave product **3ca** in 75% yield. When halogen substituents (F, Cl, Br) were present at the *para* position (R^2^), the reaction proceeded smoothly with good activity, reaching completion within 6–7 h. Products **3ea**, **3fa**, and **3ga** were obtained in 82%, 90%, and 93% yield, respectively. Electron-donating groups (methoxy, methyl) at the *para* position were also compatible, albeit with longer reaction times, and the yields of products **3ha** and **3ia** remained satisfactory. Furthermore, after replacing the aromatic ring with a naphthyl or a β-thienyl ring, the reaction still proceeded smoothly, affording the target products **3ja** (82%) and **3la** (92%) in good yields.

When a methoxy group was present at the *meta* position of the aromatic ring, only a trace amount of product **3na** was detected. When a strongly electron-donating group (dimethylamino) was located at the *para* position, the cascade cycloaddition was completely suppressed and did not occur as expected. The same outcome was observed when the substituent was changed to a moderately to strongly electron-donating group (hydroxy). When R^1^ was replaced by an ethyl or a phenyl group, the Michael/cyclization cascade reaction performed poorly: product **3ka** was obtained in only 61% yield, while product **3ma** could only be detected as a trace, presumably due to steric hindrance.

Subsequently, the diversity of the target products was examined by introducing various substituents onto the 2-(2-oxoindolin-3-yl)malononitrile component. It was found that both the electronic nature and the position of the substituents significantly influenced the reaction outcome. When electron-withdrawing groups were introduced at the 5-position, products **3ab** and **3ad** were obtained in only moderate yields. In contrast, electron-donating groups at the same position, as exemplified by **3ac** and **3ae**, led to excellent results. Furthermore, the introduction of electron-withdrawing groups at the 4- and 6-positions also proved to be well tolerated, affording the corresponding products in good yields (**3af**, **3ag**). Finally, the installation of allyl or benzyl groups at the 1-position of the oxindole ring still allowed the reaction to proceed smoothly, delivering the desired products in 92% and 87% yields (**3ah**, **3ai**).

**Scheme 3:**
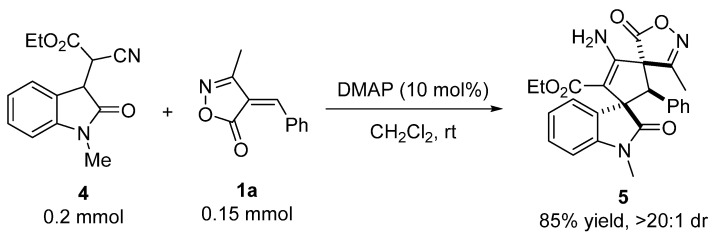
Substrate scope of ethyl 2-cyano-2-(1-methyl-2-oxoindolin-3-yl)acetate.

After a systematic investigation of the substrate scope of the Michael/cyclization cascade reaction, a specifically designed substrate, ethyl 2-cyano-2-(1-methyl-2-oxoindolin-3-yl)acetate (**4**), was synthesized. Under the standard reaction conditions, compound **4** was subjected to the cascade reaction with unsaturated isoxazolones, and the results showed that the catalytic system exhibited good reactivity, affording product **5** in 85% yield (as shown in Scheme 3). This outcome demonstrated that even a rationally designed less active substrate could still be efficiently transformed in this catalytic system.

### 2.3. Scaled-Up Synthesis

**Scheme 4:**
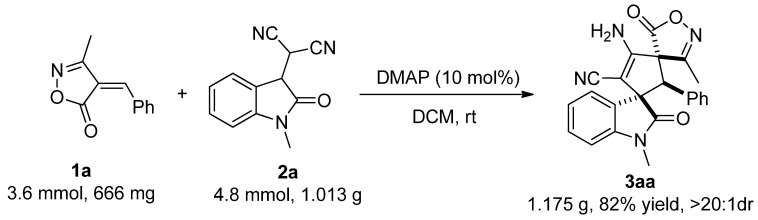
Gram-scale synthesis of **3aa**.

To evaluate the synthetic application potential of this Michael addition/cyclization cascade reaction, a gram-scale preparation was carried out under the optimized reaction conditions (as shown in Scheme 4). When the amount of reactants was scaled up to the gram level, the isolated yield of the target product showed a certain decrease compared to that obtained in the 0.1 mmol-scale reaction. Nevertheless, the stereochemical control of the product remained high during the scale-up process. This observation suggests that the stereochemistry-determining step in the reaction mechanism is only marginally affected by the reaction scale, whereas the change in yield might be related to engineering factors such as mass transfer efficiency or local concentration gradients.

### 2.4. X-Ray Crystal Structure

**Figure 2:**
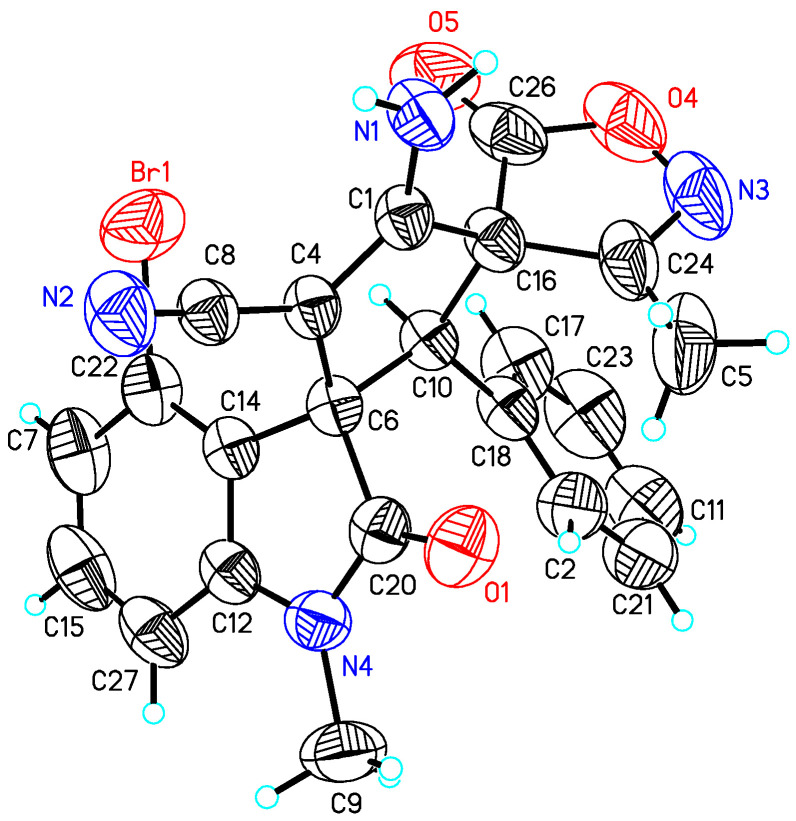
X-ray crystal structure of **3af** (one solvent ethyl acetate molecule was omitted for clarity).

The stereochemical structure of compound **3af** was determined by X-ray single-crystal diffraction analysis. The experimental results showed that the major diastereoisomer adopts a relative configuration of rel-(2’*S*,3*R*,3’*R*) [28] (Figure 2). Based on these structural elucidation results, the relative configurations of the other homologues were effectively assigned by analogy.

### 2.5. Reaction Mechanism

**Scheme 5:**
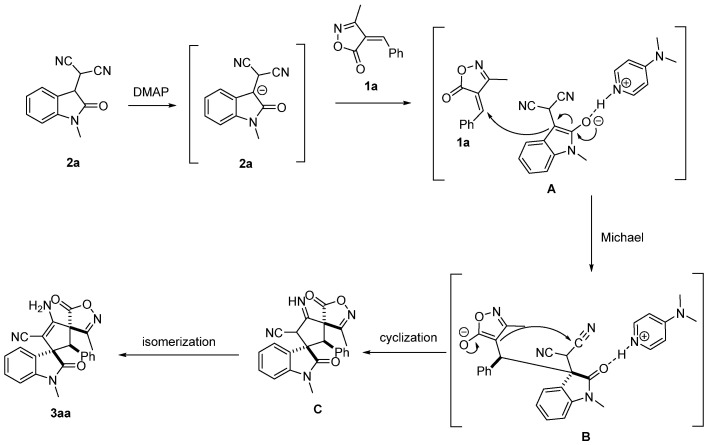
Proposed reaction mechanism.

To better understand the Michael addition/cyclization cascade reaction developed in this work, a plausible reaction mechanism is proposed based on the relative configuration of product **3af** (Scheme 5). First, the methine group of 2-(1-methyl-2-oxoindolin-3-yl)malononitrile **2a** is deprotonated by DMAP to generate an enolate. This enolate attacks the β-position of 3-methyl-4-benzylideneisoxazol-5-one **1a** via transition state **A**, leading to the formation of enolate intermediate **B** through a Michael addition. Subsequently, intermediate **B** undergoes an intramolecular cyclization to afford imine intermediate **C**, which then isomerizes to give the bispirocyclic product **3aa** and regenerates the catalyst for the next cycle.

## 3. Materials and Methods

### 3.1. General Information

Commercially available compounds were used without further purification. Solvents were dried according to standard procedures. Column chromatography was performed with silica gel (200–300 mesh) (Xinnuo, Yantai, China). Melting points were determined with a WRX-4 melting-point apparatus (Yice, Shanghai, China) and are uncorrected. ^1^H NMR spectra were measured with a Bruker Ascend 400 MHz spectrometer (Billerica, MA, USA), chemical shifts were reported in *δ* (ppm) units relative to tetramethylsilane (TMS) as internal standard. ^13^C NMR spectra were measured at 101 MHz with a 400 MHz spectrometer, chemical shifts are reported in ppm relative to tetramethylsilane and referenced to solvent peak (DMSO-d_6_, δC = 39.43). The ^19^F NMR spectra were measured at 376 MHz. Proton coupling patterns are intricately characterized as diverse spectroscopic signatures, ranging from singlets (s) to doublets (d), triplets (t), quartets (q), multiplets (m), and broadened signals (br s). High-resolution mass spectra were acquired using the Agilent 6520 accurate-mass-Q-TOF MS system (Agilent, Beijing, China), which incorporates an electrospray ionization (ESI+) source.

### 3.2. Experimental Materials

A series of unsaturated isoxazolones were synthesized according to procedures in the literature [29,30,31], while the synthesis of 2-(2-oxoindolin-3-yl)malononitriles **2a−j** followed procedures in the literature, the synthesis of organocatalysts followed procedures in the literature [32,33,34].

### 3.3. General Procedure for the Synthesis of Spirooxindole–Cyclopentene–Isoxazolones 3

Under a dry atmosphere, a 4 mL reaction vial was charged with 4-unsaturated isoxazol-5-one **1** (0.15 mmol) and 2-(2-oxoindolin-3-yl)malononitrile **2** (0.20 mmol) using a precision analytical balance. Then, 10 mol% of 4-dimethylaminopyridine (DMAP) was added as the catalyst. The reaction mixture was dissolved in 1.0 mL of CH_2_Cl_2_, and the resulting mixture was stirred magnetically at 25 ± 2 °C. The reaction progress was monitored by thin-layer chromatography (eluent: petroleum ether/ethyl acetate = 3:1, *v*/*v*). After complete consumption of the starting materials, the mixture was concentrated under reduced pressure, and the residue was purified by flash column chromatography on silica gel using a gradient elution of CH_2_Cl_2_/ethyl acetate from 10:1 to 3:1 (*v*/*v*) to afford the desired product **3**.

*rel-(2’S,3S,3’R)-4’-amino-1,3’’-dimethyl-2,5’’-dioxo-2’-phenyl-5’’H-dispiro[indoline-3,1’-cyclopentane-3’,4’’-isoxazol]-4’-ene-5’-carbonitrile* (**3aa**): according to the general procedure, the product was obtained as a white solid (54.9 mg, 92% yield), m.p. 237–240 °C; ^1^H NMR (400 MHz, DMSO-*d*_6_): δ 8.14 (d, *J* = 7.2 Hz, 1H), 7.46 (s, 2H), 7.31 (td, *J*_1_ = 7.6 Hz, *J*_2_ = 0.8 Hz, 1H), 7.22–7.15 (m, 2H), 7.08 (t, *J* = 7.6 Hz, 2H), 6.98–6.91 (m, 3H), 4.22 (s, 1H), 3.05 (s, 3H), 2.28 (s, 3H) ppm; ^13^C NMR (101 MHz, DMSO-*d*_6_): δ 176.2, 175.94, 175.89, 165.7, 155.9, 143.1, 130.7, 129.5, 129.4, 129.0, 128.4, 127.7, 126.9, 122.5, 115.5, 109.0, 79.4, 67.1, 62.8, 56.9, 26.5, 11.9 ppm. HRMS (ESI): *m/z* calcd. for C_23_H_19_N_4_O_3_ [M + H]^+^ 399.1452, found 399.1449.
*rel-(2’S,3S,3’R)-4’-amino-1,3’’-dimethyl-2,5’’-dioxo-2’-(o-tolyl)-5’’H-dispiro[indoline-3,1’-cyclopentane-3’,4’’-isoxazol]-4’-ene-5’-carbonitrile* (**3ba**): according to the general procedure, the product was obtained as a white solid (47.0 mg, 76% yield), m.p. 239–242 °C; ^1^H NMR (400 MHz, DMSO-*d*_6_): δ 8.07 (dd, *J*_1_ = 7.4 Hz, *J*_2_ = 0.6 Hz, 1H), 7.47 (s, 2H), 7.28 (td, *J*_1_ = 7.6 Hz, *J*_2_ = 1.2 Hz, 1H), 7.20 (td, *J*_1_ = 7.6, *J*_2_ = 0.8 Hz, 1H), 7.06 (t, *J* = 6.4 Hz, 2H), 7.02–6.98 (m, 1H), 6.88 (d, *J* = 7.6 Hz, 1H), 6.76 (t, *J* = 7.4 Hz, 1H), 4.72 (s, 1H), 3.04 (s, 3H), 2.29 (s, 3H), 2.21 (s, 3H) ppm; ^13^C NMR (101 MHz, DMSO-*d*_6_): δ 176.4, 176.2, 165.7, 155.8, 143.0, 137.4, 130.5, 129.4, 129.2, 128.9, 128.3, 127.6, 127.0, 125.3, 122.4, 115.5, 108.9, 79.4, 67.1, 62.9, 50.7, 26.5, 19.2, 12.2 ppm. HRMS (ESI): *m/z* calcd. for C_24_H_21_N_4_O_3_ [M + H]^+^ 413.1608, found 413.1622.
*rel-(2’S,3S,3’R)-4’-amino-2’-(2-fluorophenyl)-1,3’’-dimethyl-2,5’’-dioxo-5’’H-dispiro[indoline-3,1’-cyclopentane-3’,4’’-isoxazol]-4’-ene-5’-carbonitrile* (**3ca**): according to the general procedure, the product was obtained as a white solid (46.8 mg, 75% yield), m.p. 225–228 °C; ^1^H NMR (400 MHz, DMSO-*d*_6_): δ 8.13 (d, *J* = 7.2 Hz, 1H), 7.53 (s, 2H), 7.35 (td, *J*_1_ = 7.7 Hz, *J*_2_ = 1.1 Hz, 1H), 7.26–7.21 (m, 2H), 7.15–7.10 (m, 1H), 7.01–6.95 (m, 2H), 6.86–6.81 (m, 1H), 4.77 (d, *J* = 0.8 Hz, 1H), 3.06 (s, 3H), 2.25 (s, 3H) ppm; ^13^C NMR (101 MHz, DMSO-*d*_6_): δ 176.0, 175.5, 165.6, 160.4 (^1^*J*_C-F_ = 247.0 Hz), 155.4, 143.2, 131.0 (^3^*J*_C-F_ = 9.0 Hz), 129.84, 129.83, 129.76, 127.3, 126.8, 124.1 (^4^*J*_C-F_ = 3.3 Hz), 122.6, 117.3 (^3^*J*_C-F_ = 12.0 Hz), 115.268 (^2^*J*_C-F_ = 23.5 Hz), 115.264, 109.1, 79.5, 66.8, 62.1, 55.9, 26.5, 11.6 ppm. ^19^F NMR (376 MHz, DMSO-*d*_6_) δ −117.6 ppm. HRMS (ESI): *m/z* calcd. for C_23_H_18_FN_4_O_3_ [M + H]^+^ 417.1357, found 417.1378.
*rel-(2’S,3S,3’R)-4’-amino-2’-(2-methoxyphenyl)-1,3’’-dimethyl-2,5’’-dioxo-5’’H-dispiro[indoline-3,1’-cyclopentane-3’,4’’-isoxazol]-4’-ene-5’-carbonitrile* (**3da**): according to the general procedure, the product was obtained as a white solid (46.82mg, 72% yield), m.p. 225–228 °C; ^1^H NMR (500 MHz, DMSO-*d*_6_): δ 8.10 (d, *J* = 7.0 Hz, 1H), 7.39 (s, 2H), 7.34 (t, *J* = 7.5 Hz), 7.21 (t, *J* = 7.5 Hz, 1H), 7.11 (t, *J* = 7.8 Hz, 1H), 6.94 (d, *J* = 8.0 Hz, 1H), 6.89 (d, *J* = 8.5 Hz 2H), 6.50 (t, *J* = 7.5 Hz, 1H), 5.10 (s, 1H), 3.70 (s, 3H), 3.04 (s, 3H), 2.24 (s, 3H) ppm; ^13^C NMR (101 MHz, DMSO-*d*_6_): δ 176.3, 175.8, 165.9, 157.5, 155.7, 143.3, 129.7, 129.4, 129.2, 128.1, 126.9, 122.3, 119.7, 118.9, 115.5, 111.2, 108.9, 79.9, 67.1, 62.1, 55.94, 55.86, 26.5, 11.9 ppm. HRMS (ESI): *m/z* calcd. for C_24_H_21_N_4_O_4_ [M + H]^+^ 429.1557, found 429.1563.
*rel-(2’S,3S,3’R)-4’-amino-2’-(4-bromophenyl)-1,3’’-dimethyl-2,5’’-dioxo-5’’H-dispiro[indoline-3,1’-cyclopentane-3’,4’’-isoxazol]-4’-ene-5’-carbonitrile* (**3ea**): according to the general procedure, the product was obtained as a white solid (58.6 mg, 82% yield), m.p. 242–245 °C; ^1^H NMR (400 MHz, DMSO-*d*_6_): δ 8.10 (d, *J* = 7.1 Hz, 1H), 7.50 (s, 2H), 7.36–7.30 (m, 3H), 7.21 (t, *J* = 7.3 Hz, 1H), 6.96–6.93 (m, 3H), 4.20 (s, 1H), 3.07 (s, 3H), 2.29 (s, 3H) ppm; ^13^C NMR (101 MHz, DMSO-*d*_6_): δ 176.0, 175.8, 165.7, 155.7, 143.1, 131.5, 131.3, 129.9, 129.6, 127.3, 126.7, 122.7, 122.6, 115.4, 109.1, 79.1, 66.8, 62.5, 56.0, 26.5, 11.8 ppm. HRMS (ESI): *m/z* calcd. for C_23_H_18_^79^BrN_4_O_3_ [M + H]^+^ 477.0557, found 477.0559; calcd. for C_23_H_18_^81^BrN_4_O_3_ [M + H]^+^ 479.0536, found 479.0543.
*rel-(2’S,3S,3’R)-4’-amino-2’-(4-chlorophenyl)-1,3’’-dimethyl-2,5’’-dioxo-5’’H-dispiro[indoline-3,1’-cyclopentane-3’,4’’-isoxazol]-4’-ene-5’-carbonitrile* (**3fa**): according to the general procedure, the product was obtained as a white solid (58.3 mg, 90% yield), m.p. 234–236 °C; ^1^ H NMR (400 MHz, DMSO-*d*_6_): δ 8.10 (dd, *J*_1_ = 7.6 Hz, *J*_2_ = 0.8 Hz, 1H), 7.49 (s, 2H), 7.34 (td, *J*_1_ = 7.7 Hz, *J*_2_ =1.2 Hz, 1H), 7.23–7.15 (m, 3H), 7.02–6.99 (m, 2H), 6.95 (d, *J* = 7.6 Hz, 1H), 4.21 (s, 1H), 3.06 (s, 3H), 2.28 (s, 3H) ppm; ^13^C NMR (101 MHz, DMSO-*d*_6_): δ 176.0, 175.8, 165.7, 155.7, 143.1, 133.9, 131.2, 131.1, 129.6, 129.5, 128.4, 127.3, 126.7, 122.6, 115.4, 109.0, 79.1, 66.9, 62.6, 56.0, 26.5, 11.8 ppm. HRMS (ESI): *m/z* calcd. for C_23_H_18_ClN_4_O_3_ [M + H]^+^ 433.1062, found 433.1070.
*rel-(2’S,3S,3’R)-4’-amino-2’-(4-fluorophenyl)-1,3’’-dimethyl-2,5’’-dioxo-5’’H-dispiro[indoline-3,1’-cyclopentane-3’,4’’-isoxazol]-4’-ene-5’-carbonitrile* (**3ga**): according to the general procedure, the product was obtained as a white solid (58.1 mg, 93% yield), m.p. 248–250 °C; ^1^H NMR (400 MHz, DMSO-*d*_6_): δ 8.12 (d, *J* = 7.4 Hz, 1H), 7.49 (s, 2H), 7.33 (t, *J* = 7.5 Hz, 1H), 7.22 (t, *J* = 7.5 Hz, 1H), 7.06–7.02 (m, 2H), 6.97–6.92 (m, 3H), 4.23 (s, 1H), 3.06 (s, 3H), 2.29 (s, 3H) ppm; ^13^C NMR (101 MHz, DMSO-*d*_6_): δ 176.1, 175.8, 165.7, 162.1 (^1^*J*_C-F_ = 247.6 Hz), 155.7, 143.1, 131.6 (^3^*J*_C-F_ = 8.5 Hz), 129.6, 127.5, 126.8 (^4^*J*_C-F_ = 3.1 Hz), 126.7, 122.5, 115.4, 115.3 (^2^*J*_C-F_ = 21.5 Hz), 109.0, 79.1, 67.0, 62.7, 56.0, 26.5, 11.8 ppm. ^19^F NMR (376 MHz, DMSO-*d*_6_): δ −112.4 ppm. HRMS (ESI): *m/z* calcd. for C_23_H_18_FN_4_O_3_ [M + H]^+^ 417.1357, found 417.1373.
*rel-(2’S,3S,3’R)-4’-amino-1,3’’-dimethyl-2,5’’-dioxo-2’-(p-tolyl)-5’’H-dispiro[indoline-3,1’-cyclopentane-3’,4’’-isoxazol]-4’-ene-5’-carbonitrile* (**3ha**): according to the general procedure, the product was obtained as a white solid (50.7 mg, 82% yield), m.p. 240–243 °C; ^1^H NMR (400 MHz, DMSO-*d*_6_): δ 8.16–8.11 (m, 1H), 7.44 (s, 2H), 7.31 (td, *J*_1_ = 7.6 Hz, *J*_2_ = 1.2 Hz, 1H), 7.20 (td, *J*_1_ = 7.6 Hz, *J*_2_ = 0.8 Hz, 1H), 6.93 (d, *J* = 7.7 Hz, 1H), 6.90–6.83 (m, 4H), 4.18 (s, 1H), 3.05 (s, 3H), 2.26 (s, 3H), 2.12 (s, 3H) ppm; ^13^C NMR (101 MHz, DMSO-*d*_6_): δ 176.2, 175.9, 165.7, 155.8, 143.1, 138.3, 129.4, 129.3, 128.9, 127.7, 127.6, 126.8, 122.4, 115.5, 108.9, 79.3, 67.1, 62.7, 56.7, 26.5, 20.4, 11.8 ppm. HRMS (ESI): *m/z* calcd. for C_24_H_21_N_4_O_3_ [M + H]^+^ 413.1608, found 413.1621.
*rel-(2’S,3S,3’R)-4’-amino-2’-(4-methoxyphenyl)-1,3’’-dimethyl-2,5’’-dioxo-5’’H-dispiro[indoline-3,1’-cyclopentane-3’,4’’-isoxazol]-4’-ene-5’-carbonitrile* (**3ia**): according to the general procedure, the product was obtained as a white solid (51.5 mg, 80% yield), m.p. 240–243 °C; ^1^H NMR (400 MHz, DMSO-*d*_6_): δ 8.14 (d, *J* = 7.0 Hz, 1H), 7.44 (s, 2H), 7.32 (td, *J*_1_ = 7.6 Hz, *J*_2_ = 1.2 Hz, 1H), 7.24–7.17 (m, 1H), 6.94–6.89 (m, 3H), 6.62 (d, *J* = 8.8 Hz, 2H), 4.18 (s, 1H), 3.61 (s, 3H), 3.05 (s, 3H), 2.26 (s, 3H) ppm; ^13^C NMR (101 MHz, DMSO-*d*_6_): δ 176.3, 175.9, 165.7, 159.3, 155.8, 143.2, 130.7, 129.4, 127.8, 126.8, 122.4, 122.3, 115.5, 113.6, 108.9, 79.3, 67.2, 62.8, 56.4, 54.8, 26.5, 11.8 ppm. HRMS (ESI): *m/z* calcd. for C_24_H_21_N_4_O_4_ [M + H]^+^ 429.1557, found 429.1564.
*rel-(2’S,3S,3’R)-4’-amino-1,3’’-dimethyl-2,5’’-dioxo-2’-(thiophen-2-yl)-5’’H-dispiro[indoline-3,1’-cyclopentane-3’,4’’-isoxazol]-4’-ene-5’-carbonitrile* (**3ja**): according to the general procedure, the product was obtained as a white solid (49.7 mg, 82% yield), m.p. 217–220 °C; ^1^H NMR (400 MHz, DMSO-*d*_6_): δ 8.14 (d, *J* = 7.4 Hz, 1H), 7.48 (s, 2H), 7.37 (t, *J* = 7.6 Hz, 1H), 7.32–7.30 (m, 1H), 7.21 (t, *J* = 7.5 Hz, 1H), 6.99 (d, *J* = 7.8 Hz, 1H), 6.82–6.78 (m, 2H), 4.58 (s, 1H), 3.10 (s, 3H), 2.28 (s, 3H) ppm; ^13^C NMR (101 MHz, DMSO-*d*_6_): δ 175.9, 175.2, 165.4, 155.2, 143.5, 131.5, 129.9, 129.1, 128.0, 127.3, 127.2, 126.2, 122.6, 115.3, 108.9, 79.1, 67.3, 62.3, 52.3, 26.6, 11.7 ppm. HRMS (ESI): *m/z* calcd. for C_21_H_17_N_4_O_3_S [M + H]^+^ 405.1016, found 405.1025.
*rel-(2’S,3S,3’R)-4’-amino-3’’-ethyl-1-methyl-2,5’’-dioxo-2’-phenyl-5’’H-dispiro[indoline-3,1’-cyclopentane-3’,4’’-isoxazol]-4’-ene-5’-carbonitrile* (**3ka**): according to the general procedure, the product was obtained as a white solid (37.7 mg, 61% yield), m.p. 244–247 °C; ^1^H NMR (400 MHz, DMSO-*d*_6_): δ 8.12 (d, *J* = 6.8 Hz, 1H), 7.46 (s, 2H), 7.30 (td, *J*_1_ = 7.8 Hz, *J*_2_ = 1.2 Hz, 1H), 7.22–7.14 (m, 2H), 7.08 (t, *J* = 7.6 Hz, 2H), 6.96–6.90 (m, 3H), 4.25 (s, 1H), 3.05 (s, 3H), 2.87–2.75 (m, 1H), 2.61–2.52 (m, 1H), 1.26 (t, *J* = 7.3 Hz, 3H) ppm; ^13^C NMR (101 MHz, DMSO-*d*_6_): δ 176.2, 176.1, 169.1, 156.2, 143.1, 130.7, 129.4, 129.3, 128.8, 128.3, 127.7, 126.8, 122.5, 115.5, 108.9, 79.2, 67.0, 62.7, 57.0, 26.5, 20.2, 8.6 ppm. HRMS (ESI): *m/z* calcd. for C_24_H_21_N_4_O_3_ [M + H]^+^ 413.1608, found 413.1623.
*rel-(2’S,3S,3’R)-4’-amino-1,3’’-dimethyl-2’-(naphthalen-2-yl)-2,5’’-dioxo-5’’H-dispiro[indoline-3,1’-cyclopentane-3’,4’’-isoxazol]-4’-ene-5’-carbonitrile* (**3la**): according to the general procedure, the product was obtained as a white solid (61.9 mg, 92% yield), m.p. 173–175 °C; ^1^H NMR (400 MHz, DMSO-*d*_6_): δ 8.25 (d, *J* = 7.1 Hz, 1H), 7.78–7.72 (m, 1H), 7.70–7.65 (m, 1H), 7.63–7.57 (m, 2H), 7.52 (s, 2H), 7.49–7.43 (m, 2H), 7.33–7.23 (m, 2H), 7.09 (d, *J* = 7.9 Hz, 1H), 6.87 (d, *J* = 7.5 Hz, 1H), 4.41 (s, 1H), 3.02 (s, 3H), 2.35 (s, 3H) ppm; ^13^C NMR (101 MHz, DMSO-*d*_6_): δ 176.2, 175.9, 165.8, 155.9, 143.1, 132.5, 132.1, 129.6, 129.5, 128.2, 127.69, 127.66, 127.2, 127.0, 126.8, 126.5, 126.1, 122.5, 115.5, 109.0, 79.2, 67.1, 62.8, 57.1, 26.4, 11.9 ppm. HRMS (ESI): *m/z* calcd. for C_27_H_21_N_4_O_3_ [M + H]^+^ 449.1608, found 449.1613.
*rel-(2’S,3S,3’R)-4’-amino-5-chloro-1,3’’-dimethyl-2,5’’-dioxo-2’-phenyl-5’’H-dispiro[indoline-3,1’-cyclopentane-3’,4’’-isoxazol]-4’-ene-5’-carbonitrile* (**3ab**): according to the general procedure, the product was obtained as a white solid (51.2 mg, 79% yield), m.p. 244–247 °C; ^1^H NMR (500 MHz, DMSO-*d*_6_): δ 8.17 (d, *J* = 2.0 Hz, 1H), 7.58 (s, 2H), 7.40 (dd, *J*_1_ = 8.3 Hz, *J*_2_ = 2.3 Hz, 1H), 7.20 (t, *J* = 7.3 Hz, 1H), 7.13 (t, *J* = 7.5 Hz, 2H), 6.99–6.95 (m, 3H), 4.22 (s, 1H), 3.06 (s, 3H), 2.28 (s, 3H) ppm; ^13^C NMR (101 MHz, DMSO-*d*_6_): δ 176.2, 175.7, 165.6, 156.2, 142.0, 130.3, 129.7, 129.4, 129.1, 128.5, 126.7, 115.2, 110.6, 78.5, 66.8, 62.8, 56.7, 26.7, 11.8 ppm. HRMS (ESI): *m/z* calcd. for C_23_H_18_ClN_4_O_3_ [M + H]^+^ 433.1062, found 433.1078.
*rel-(2’S,3S,3’R)-4’-amino-1-benzyl-3’’,5-dimethyl-2,5’’-dioxo-2’-phenyl-5’’H-dispiro[indoline-3,1’-cyclopentane-3’,4’’-isoxazol]-4’-ene-5’-carbonitrile* (**3ac**): according to the general procedure, the product was obtained as a white solid (58.7 mg, 95% yield), m.p. 243–245 °C; ^1^H NMR (400 MHz, DMSO-*d*_6_): δ 8.02 (s, 1H), 7.51 (s, 2H), 7.23 (t, *J* = 7.4 Hz, 1H), 7.16–7.04 (m, 5H), 7.00 (d, *J* = 7.5 Hz, 3H), 6.79 (d, *J* = 7.3 Hz, 2H), 6.56 (d, *J* = 8.0 Hz, 1H), 4.94 (d, *J* = 16.2 Hz, 1H), 4.72 (d, *J* = 16.2 Hz, 1H), 4.28 (s, 1H), 2.35 (s, 3H), 2.30 (s, 3H) ppm; ^13^C NMR (101 MHz, DMSO-*d*_6_): δ 176.4, 175.8, 165.7, 155.9, 139.8, 135.2, 131.4, 130.5, 129.7, 129.6, 129.0, 128.4, 128.2, 127.9, 127.7, 127.0, 126.2, 115.7, 109.2, 79.2, 66.9, 62.9, 57.3, 42.8, 20.9, 11.8 ppm. HRMS (ESI): *m/z* calcd. for C_24_H_21_N_4_O_3_ [M + H]^+^ 489.1921, found 489.1930.
*rel-(2’S,3S,3’R)-4’-amino-5-fluoro-1,3’’-dimethyl-2,5’’-dioxo-2’-phenyl-5’’H-dispiro[indoline-3,1’-cyclopentane-3’,4’’-isoxazol]-4’-ene-5’-carbonitrile* (**3ad**): according to the general procedure, the product was obtained as a white solid (49.9 mg, 80% yield), m.p. 224−227 °C; ^1^H NMR (400 MHz, DMSO-*d*_6_): δ 7.97 (dd, *J*_1_ = 8.8 Hz, *J*_2_ = 2.7 Hz, 1H), 7.56 (s, 2H), 7.23–7.11 (m, 4H), 6.99–6.94 (m, 3H), 4.24 (s, 1H), 3.06 (s, 3H), 2.28 (s, 3H) ppm; ^13^C NMR (101 MHz, DMSO-*d*_6_): δ 176.2, 175.8, 165.6, 158.4 (^1^*J*_C-F_ = 238.9 Hz), 156.1, 139.4 (^4^*J*_C-F_ = 1.6 Hz), 130.4, 129.5 (^3^*J*_C-F_ = 8.1 Hz), 129.10, 129.05, 128.5, 115.9 (^2^*J*_C-F_ = 23.6 Hz), 115.2, 114.3 (^2^*J*_C-F_ = 26.3 Hz), 110.0 (^3^*J*_C-F_ = 8.2 Hz), 78.7, 66.8, 63.0, 56.7, 26.7, 11.8 ppm; ^19^F NMR (376 MHz, DMSO-*d*_6_) δ −120.1 ppm. HRMS (ESI): *m/z* calcd. for C_23_H_18_FN_4_O_3_ [M + H]^+^ 417.1357, found 417.1360.
*rel-(2’S,3S,3’R)-4’-amino-1,3’’,5,7-tetramethyl-2,5’’-dioxo-2’-phenyl-5’’H-dispiro[indoline-3,1’-cyclopentane-3’,4’’-isoxazol]-4’-ene-5’-carbonitrile* (**3ae**): according to the general procedure, the product was obtained as a white solid (61.4 mg, 96% yield), m.p. 151−154 °C; ^1^H NMR (400 MHz, DMSO-*d*_6_): δ 7.84 (s, 1H), 7.40 (s, 2H), 7.18 (t, *J* = 7.3 Hz, 1H), 7.09 (t, *J* = 7.6 Hz, 2H), 6.96 (d, *J* = 7.4 Hz, 2H), 6.87 (s, 1H), 4.20 (s, 1H), 3.29 (s, 3H), 2.37 (s, 3H), 2.32 (s, 3H), 2.26 (s, 3H) ppm; ^13^C NMR (101 MHz, DMSO-*d*_6_): δ 176.8, 175.8, 165.7, 155.5, 138.5, 133.4, 131.0, 130.8, 129.5, 128.8, 128.4, 128.3, 125.5, 119.5, 115.6, 80.1, 67.0, 62.2, 57.0, 29.6, 20.6, 18.2, 11.8 ppm. HRMS (ESI): *m/z* calcd. for C_25_H_23_N_4_O_3_ [M + H]^+^ 427.1765, found 427.1776.
*rel-(2’S,3S,3’R)-4’-amino-4-bromo-1,3’’-dimethyl-2,5’’-dioxo-2’-phenyl-5’’H-dispiro[indoline-3,1’-cyclopentane-3’,4’’-isoxazol]-4’-ene-5’-carbonitrile* (**3af**): according to the general procedure, the product was obtained as a white solid (62.8 mg, 88% yield), m.p. 148−151 °C; ^1^H NMR (400 MHz, DMSO-*d*_6_): δ 7.63 (s, 2H), 7.33 (dd, *J*_1_ = 8.0 Hz, *J*_2_ = 0.8 Hz, 1H), 7.29–7.20 (m, 4H), 6.94 (dd, *J*_1_ = 7.6 Hz, *J*_2_ = 0.4 Hz, 1H), 6.86 (d, *J* = 7.1 Hz, 2H), 4.86 (s, 1H), 2.96 (s, 3H), 2.60 (s, 3H) ppm; ^13^C NMR (101 MHz, DMSO-*d*_6_): δ 177.2, 175.0, 166.1, 157.5, 145.1, 131.5, 130.4, 129.2, 128.8, 128.4, 127.4, 124.6, 118.4, 115.6, 108.4, 75.7, 66.7, 64.2, 59.7, 26.3, 14.4 ppm. HRMS (ESI): *m/z* calcd. for C_23_H_18_^79^BrN_4_O_3_ [M + H]^+^ 477.0557, found 477.0533; calcd. for C_23_H_18_^81^BrN_4_O_3_ [M + H]^+^ 479.0536, found 479.0524.
*rel-(2’S,3S,3’R)-4’-amino-6-chloro-1,3’’-dimethyl-2,5’’-dioxo-2’-phenyl-5’’H-dispiro[indoline-3,1’-cyclopentane-3’,4’’-isoxazol]-4’-ene-5’-carbonitrile* (**3ag**): according to the general procedure, the product was obtained as a white solid (60.3 mg, 93%% yield), m.p. 229−231 °C; ^1^H NMR (400 MHz, DMSO-*d*_6_): δ 8.12 (d, *J* = 8.1 Hz, 1H), 7.54 (s, 2H), 7.29 (dd, *J*_1_ = 8.1 Hz, *J*_2_ = 1.9 Hz, 1H), 7.22–7.17 (m, 1H), 7.15–7.09 (m, 3H), 6.97 (d, *J* = 7.4 Hz, 2H), 4.21 (s, 1H), 3.06 (s, 3H), 2.28 (s, 3H) ppm; ^13^C NMR (101 MHz, DMSO-*d*_6_): δ 176.3, 176.0, 165.6, 156.0, 144.6, 134.0, 130.4, 129.3, 129.1, 128.5, 128.1, 126.5, 122.3, 115.3, 109.6, 78.7, 66.9, 62.4, 56.8, 26.7, 11.8 ppm. HRMS (ESI): *m/z* calcd. for C_23_H_18_ClN_4_O_3_ [M + H]^+^ 433.1062, found 433.1051.
*rel-(2’S,3S,3’R)-1-allyl-4’-amino-3’’-methyl-2,5’’-dioxo-2’-phenyl-5’’H-dispiro[indoline-3,1’-cyclopentane-3’,4’’-isoxazol]-4’-ene-5’-carbonitrile* (**3ah**): according to the general procedure, the product was obtained as a white solid (58.5 mg, 92% yield), m.p. 220−223 °C; ^1^H NMR (400 MHz, DMSO-*d*_6_): δ 8.16 (dd, *J*_1_ = 7.2 Hz, *J*_2_ = 0.8 Hz, 1H), 7.50 (s, 2H), 7.27 (td, *J*_1_ = 7.6 Hz, *J*_2_ = 1.2 Hz, 1H), 7.23–7.15 (m, 2H), 7.08 (t, *J* = 7.7 Hz, 2H), 6.98 (d, *J* = 7.5 Hz, 2H), 6.79 (d, *J* = 7.7 Hz, 1H), 5.68–5.58 (m, 1H), 4.83 (dd, *J*_1_ = 10.8 Hz, *J*_2_ = 0.8 Hz, 1H), 4.39 (dd, *J*_1_ = 17.6 Hz, *J*_2_ = 0.8 Hz, 1H), 4.433–4.27 (m, 1H), 4.22–4.14 (m, 2H), 2.281 (s, 3H) ppm; ^13^C NMR (101 MHz, DMSO-*d*_6_): δ 176.1, 175.8, 165.7, 155.9, 142.2, 130.7, 130.5, 129.6, 129.4, 129.0, 128.3, 127.7, 126.9, 122.4, 115.6, 115.2, 109.5, 79.1, 67.0, 62.8, 57.1, 41.5, 11.8 ppm. HRMS (ESI): *m/z* calcd. for C_25_H_21_N_4_O_3_ [M + H]^+^ 425.1608, found 425.1616.
*rel-(2’S,3S,3’R)-4’-amino-1-benzyl-3’’-methyl-2,5’’-dioxo-2’-phenyl-5’’H-dispiro[indoline-3,1’-cyclopentane-3’,4’’-isoxazol]-4’-ene-5’-carbonitrile* (**3ai**): according to the general procedure, the product was obtained as a white solid (61.9 mg, 87% yield), m.p. 233−236 °C; ^1^H NMR (400 MHz, DMSO-*d*_6_): δ 8.19–8.16 (m, 1H), 7.53 (s, 2H), 7.24–7.06 (m, 8H), 7.00 (d, *J* = 7.4 Hz, 2H), 6.81 (d, *J* = 7.2 Hz, 2H), 6.70–6.67 (m, 1H), 4.97 (d, *J* = 16.2 Hz, 1H), 4.75 (d, *J* = 16.2 Hz, 1H), 4.29 (s, 1H), 2.30 (s, 3H) ppm; ^13^C NMR (101 MHz, DMSO-*d*_6_): δ 176.5, 175.8, 165.7, 156.1, 142.1, 135.1, 130.4, 129.7, 129.4, 129.0, 128.33, 128.27, 127.8, 127.1, 127.0, 126.2, 122.5, 115.6, 109.5, 79.0, 66.9, 62.9, 57.3, 42.7, 38.8, 11.8 ppm. HRMS (ESI): *m/z* calcd. for C_29_H_23_N_4_O_3_ [M + H]^+^ 475.1765, found 475.1776.
*ethyl rel-(2’S,3S,3’R)-4’-amino-1,3’’-dimethyl-2,5’’-dioxo-2’-phenyl-5’’H-dispiro[indoline-3,1’-cyclopentane-3’,4’’-isoxazol]-4’-ene-5’-carboxylate* (**5**): according to the general procedure, the product was obtained as a white solid (56.8 mg, 85% yield), m.p. 227−229 °C; ^1^H NMR (400 MHz, DMSO-*d*_6_): δ 8.06–8.03 (m, 1H), 7.36 (s, 2H), 7.22 (td, *J*_1_ = 7.6 Hz, *J*_2_ = 1.2 Hz, 1H), 7.15 (t, *J* = 7.3 Hz, 1H), 7.10–7.03 (m, 3H), 6.96 (d, *J* = 7.4 Hz, 2H), 6.81 (d, *J* = 7.7 Hz, 1H), 4.13 (s, 1H), 3.80–3.68 (m, 2H), 3.00 (s, 3H), 2.27 (s, 3H), 0.77 (t, *J* = 7.1 Hz, 3H) ppm; ^13^C NMR (101 MHz, DMSO-*d*_6_): δ 178.0, 176.1, 166.0, 164.4, 154.6, 143.8, 143.6, 131.1, 129.8, 129.5, 128.6, 128.4, 128.1, 126.0, 121.6, 107.9, 101.0, 67.7, 61.8, 58.6, 56.6, 26.3, 13.6, 11.9 ppm. HRMS (ESI): *m/z* calcd. for C_25_H_24_N_3_O_5_ [M + H]^+^ 446.1710, found 446.1720.

### 3.4. Procedure for the Scaled-Up Synthesis of Compound 3aa

Under a dry atmosphere, a 100 mL reaction vial was charged with 4-unsaturated isoxazol-5-one **1a** (3.6 mmol) and 2-(2-oxoindolin-3-yl)malononitrile **2a** (4.8 mmol) using a precision analytical balance. Then, 10 mol% of 4-dimethylaminopyridine (DMAP) was added as the catalyst. The reaction mixture was dissolved in 25 mL of CH_2_Cl_2_, and the resulting mixture was stirred magnetically at 25 ± 2 °C. The reaction progress was monitored by TLC (eluent: petroleum ether/ethyl acetate = 3:1, *v*/*v*). After complete consumption of the starting materials, the mixture was concentrated under reduced pressure, and the residue was purified by flash column chromatography on silica gel using a gradient elution of CH_2_Cl_2_/ethyl acetate from 10:1 to 3:1 (*v*/*v*) to afford the desired product **3aa** in 82% overall yield.

## 4. Conclusions

In summary, a DMAP-catalyzed Michael addition/cyclization reaction has been developed for the efficient construction of bispirooxindole/cyclopentene-fused isoxazolone derivatives. This protocol employs DMAP as the catalyst and CH_2_Cl_2_ as the solvent, and proceeds smoothly at room temperature, affording the target products in up to 96% isolated yield with >20:1 diastereoselectivity. Moreover, the reaction exhibits broad substrate compatibility; although substrates bearing strongly electron-withdrawing groups reduce the reaction efficiency, most derivatives still enable the efficient construction of the bispirooxindole/cyclopentene–isoxazolone scaffolds. The practical utility of this method was further demonstrated by gram-scale experiments and derivatization of the products. This strategy provides a new route for the rapid construction of a library of spiro heterocyclic compounds featuring complex structures and quaternary carbon stereocenters.

## Figures and Tables

**Table 1:** Optimization of reaction conditions.

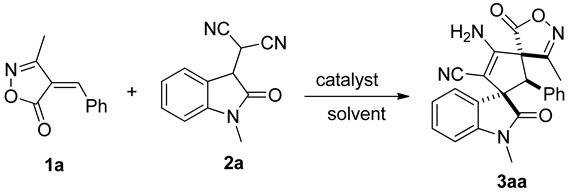			
Entry ^[a]^	Solvent	Catalyst	Yield ^[b]^ (%)
1	CH_2_Cl_2_	Et_3_N	58
2	PhMe	Et_3_N	56
3	CH3CN	Et_3_N	45
4	THF	Et_3_N	55
5	EtOH	Et_3_N	53
6	CHCl3	Et_3_N	57
7	CH_2_Cl_2_	DMAP	90
8	CH_2_Cl_2_	DIPEA	76
9	CH_2_Cl_2_	DBU	55
10	CH_2_Cl_2_	NaHCO_3_	57
11	CH_2_Cl_2_	Na_2_CO_3_	87
12	CH_2_Cl_2_	K_2_CO_3_	43
13	CH_2_Cl_2_	Cs_2_CO_3_	63
14	CH_2_Cl_2_	/	42
15 ^[c]^	CH_2_Cl_2_	DMAP	45
16 ^[d]^	CH_2_Cl_2_	DMAP	78
17 ^[e]^	CH_2_Cl_2_	DMAP	57
18 ^[f]^	CH_2_Cl_2_	DMAP	77

^[a]^ Unless otherwise specified, reactions were carried out with **1a** (0.15 mmol), **2a** (0.20 mmol) and catalyst (10 mol%) in solvent (1.0 mL) at room temperature for 12 h. Under all tested conditions, the product was obtained with a diastereomeric ratio of >20:1, as determined by ^1^H NMR analysis of the crude reaction mixture. ^[b]^ isolated yield after column chromatography purification. ^[c]^ The reaction was conducted at 50 °C. ^[d]^ The reaction was conducted at −10 °C. ^[e]^ catalyst loading: 20 mol%. ^[f]^ catalyst loading: 5 mol%.

## Data Availability

Data are contained within this article and Supplementary Materials.

## Supplementary Materials

The following supporting information can be downloaded at https://www.mdpi.com/article/10.3390/molecules31142461/s1, File S1: Copies of ^1^H and ^13^C NMR spectra of new compounds.
