# Supplementary material for: DMAP-Promoted Cascade Synthesis of Bispirooxindole–Cyclopentene–Isoxazolones
Source: Molecules. 2026 Jul 14;31(14):2461. doi: 10.3390/molecules31142461 (PMC13414304; doi:10.3390/molecules31142461)
Supplement: Supplementary file 1 [file molecules-31-02461-s001.zip › molecules-4382340-File S1.pdf]

DMAP-Promoted Cascade Synthesis of Bispirooxindole–Cyclopentene–Isoxazolones

Wei Zhang †, Rong-Rong Zhu *,† and Da-Ming Du ‡

School of Chemistry and Chemical Engineering, Beijing Institute of Technology, Beijing 100081, China; 3220231751@bit.edu.cn (W.Z.)

* Correspondence: plutorzr@outlook.com

† These authors contributed equally to this work.

‡ Deceased author.

Contents

1. Copies of ^{1}H ,^{13}C NMR and ^{19}F NMR spectra of new compounds………..........................S1-S23

1. Copies of ^{1}H , ^{13}C NMR and ^{19}F NMR spectra of new compounds

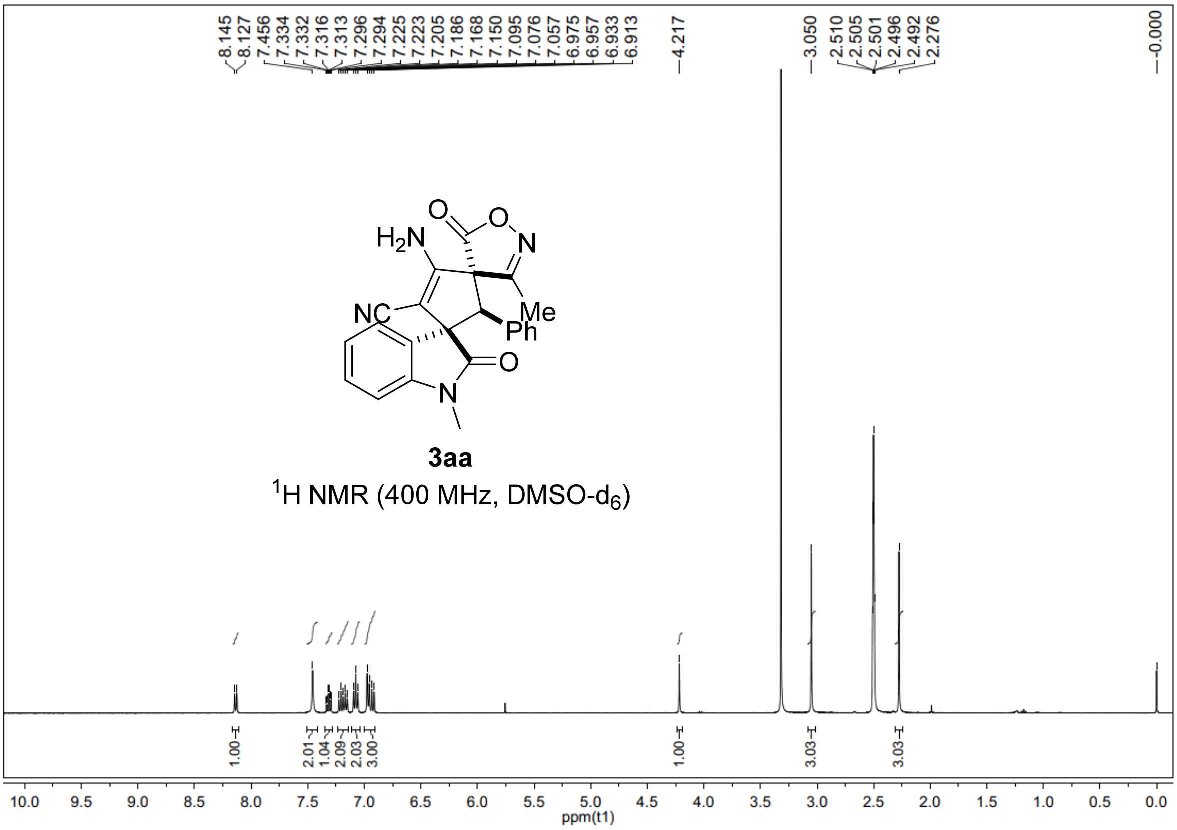

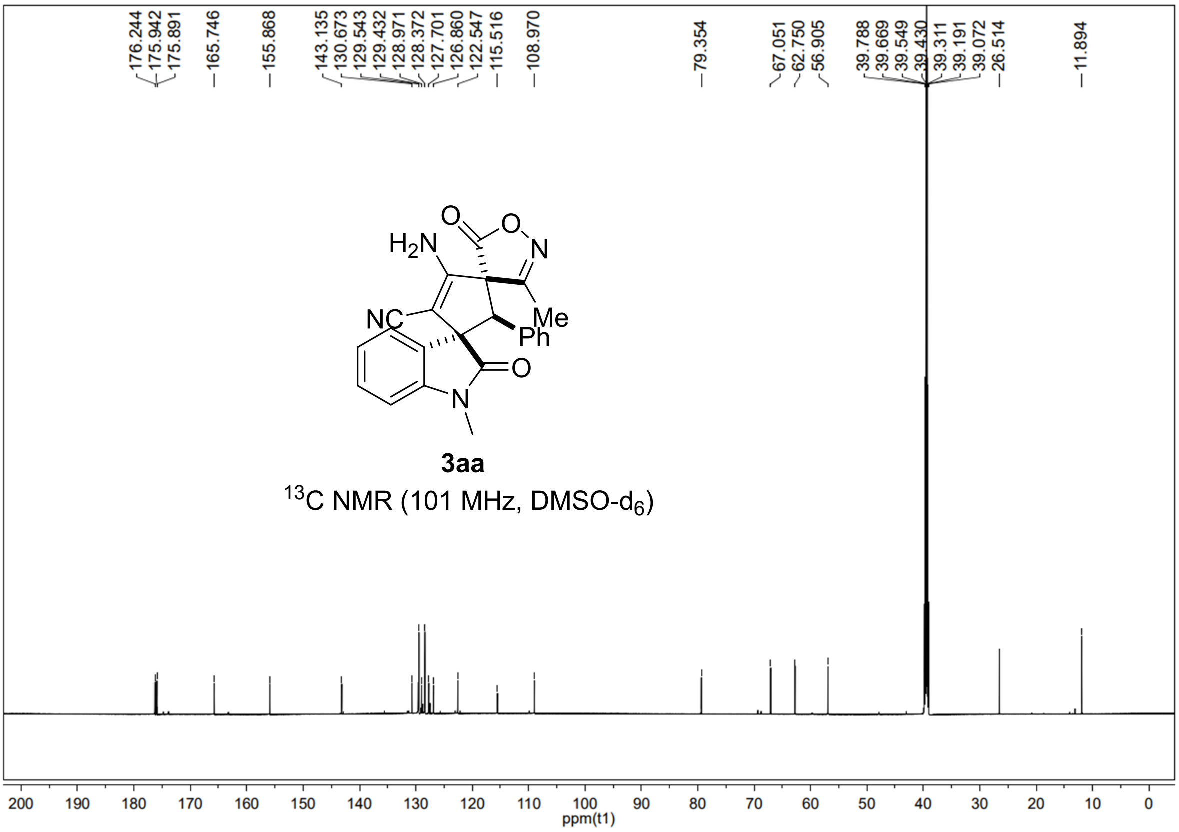

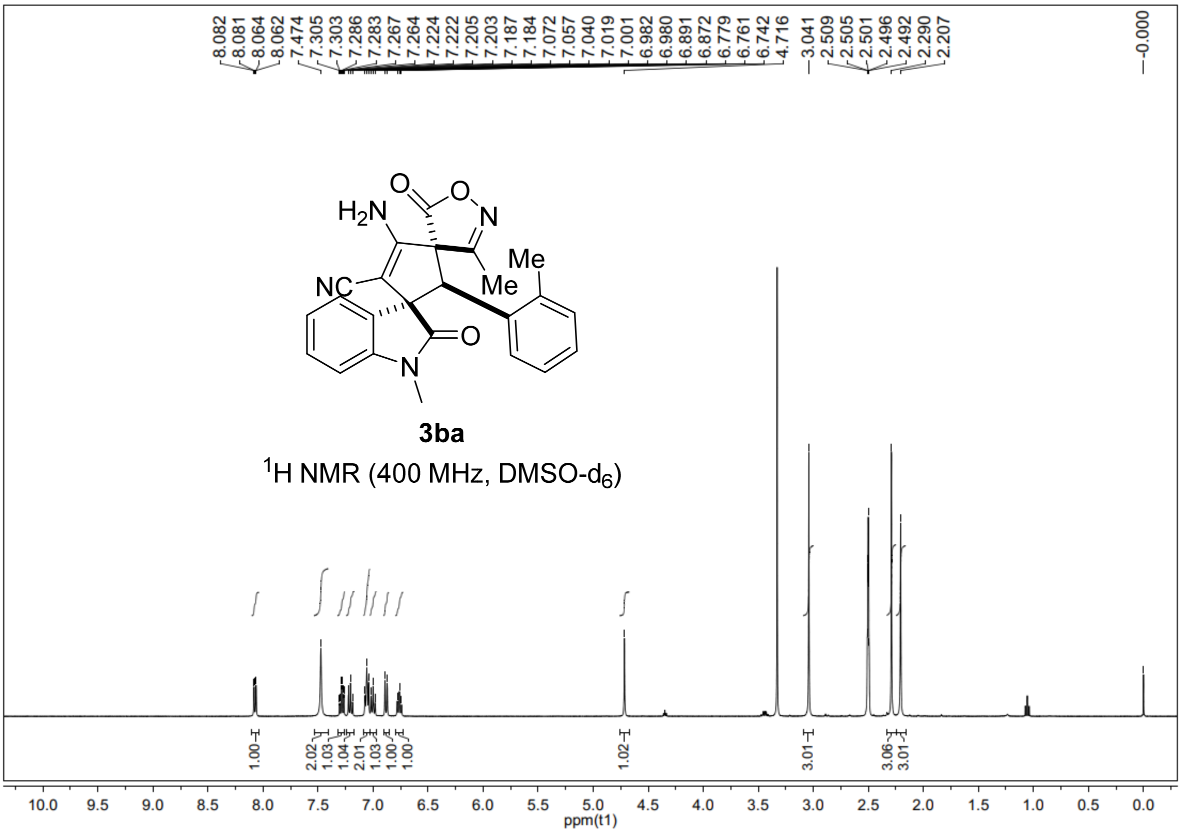
8.082
8.081
8.064
8.062
7.474
7.305
7.303
7.286
7.283
7.267
7.264
7.224
7.222
7.205
7.203
7.187
7.184
7.072
7.057
7.040
7.019
7.001
6.982
6.980
6.891
6.872
6.779
6.761
6.742
4.716
3.041
2.509
2.505
2.501
2.496
2.492
2.290
2.207
-0.000
H2N
O
O
N
Me
Me
NC
O
N
3ba
1H NMR (400 MHz, DMSO-d6)
1.00
2.02
1.03
1.04
2.01
1.03
1.00
1.00
1.02
3.01
3.06
3.01
10.0
9.5
9.0
8.5
8.0
7.5
7.0
6.5
6.0
5.5
5.0
4.5
4.0
3.5
3.0
2.5
2.0
1.5
1.0
0.5
0.0
ppm(t1)

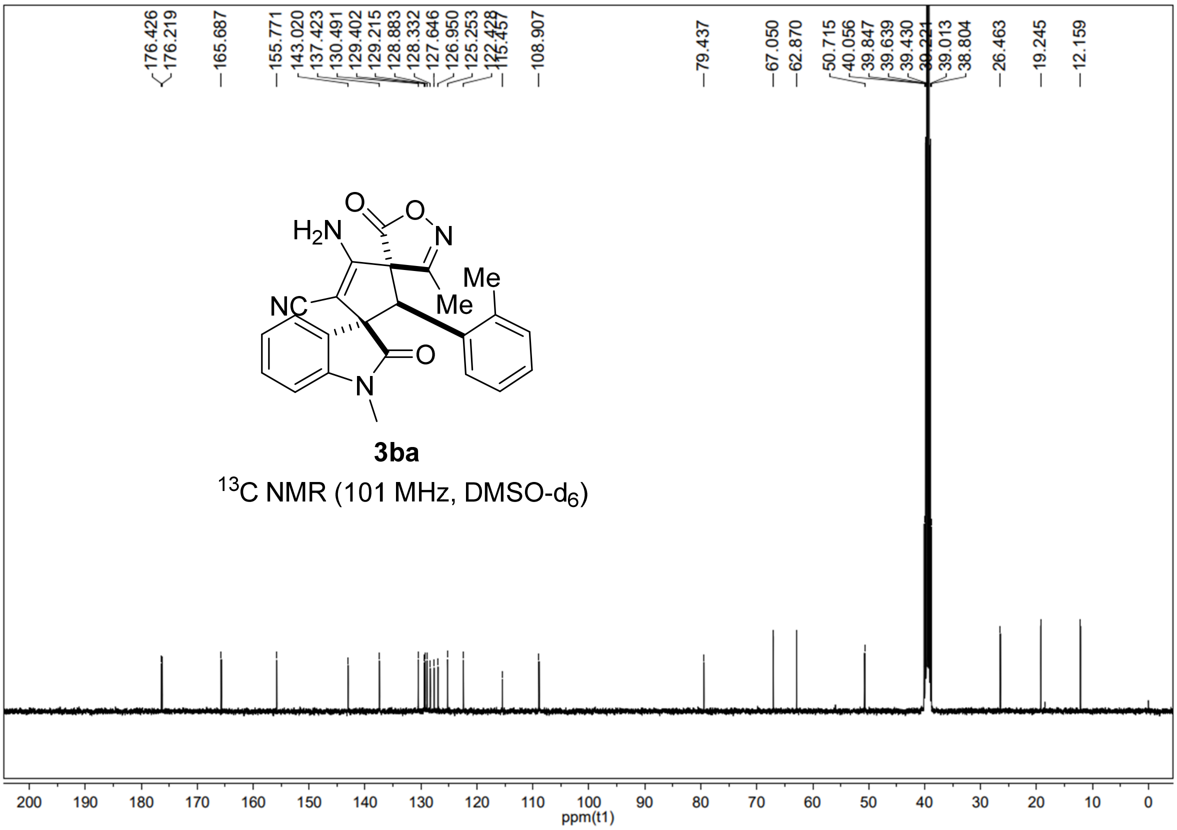
176.426
176.219
165.687
155.771
143.020
137.423
130.491
129.402
129.215
128.883
128.332
127.646
126.950
125.253
122.428
115.457
108.907
79.437
67.050
62.870
50.715
40.056
39.847
39.639
39.430
39.013
38.804
26.463
19.245
12.159
H2N
O
O
N
Me
Me
NC
O
N
3ba
13C NMR (101 MHz, DMSO-d6)
200
190
180
170
160
150
140
130
120
110
100
90
80
70
60
50
40
30
20
10
0
ppm(t1)

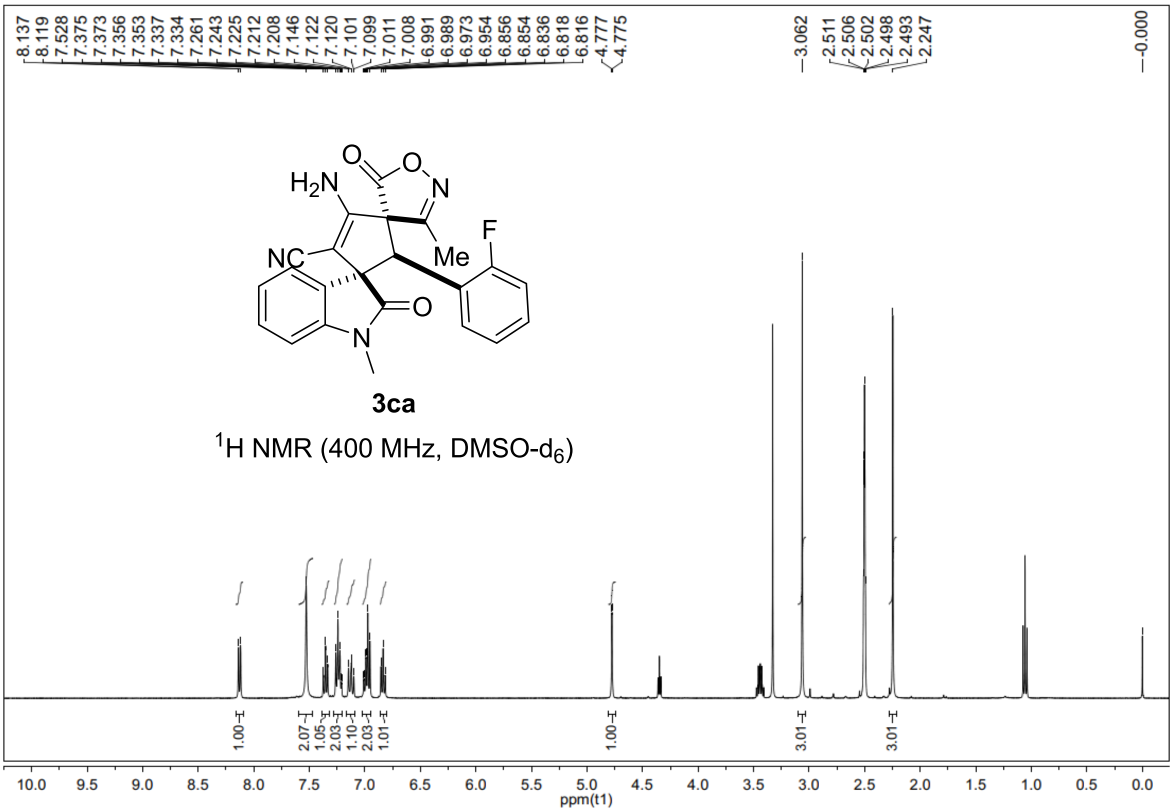
8.137
8.119
7.528
7.375
7.373
7.356
7.353
7.337
7.334
7.261
7.243
7.225
7.212
7.208
7.146
7.122
7.120
7.101
7.099
7.011
7.008
6.991
6.989
6.973
6.954
6.856
6.854
6.836
6.818
6.816
4.777
4.775
3.062
2.511
2.506
2.502
2.498
2.493
2.247
-0.000
H2N
O
O
N
NC
Me
F
O
N
3ca
1H NMR (400 MHz, DMSO-d6)
1.00
2.07
1.05
2.03
1.10
2.03
1.01
1.00
3.01
3.01
10.0
9.5
9.0
8.5
8.0
7.5
7.0
6.5
6.0
5.5
5.0
4.5
4.0
3.5
3.0
2.5
2.0
1.5
1.0
0.5
0.0
ppm(t1)

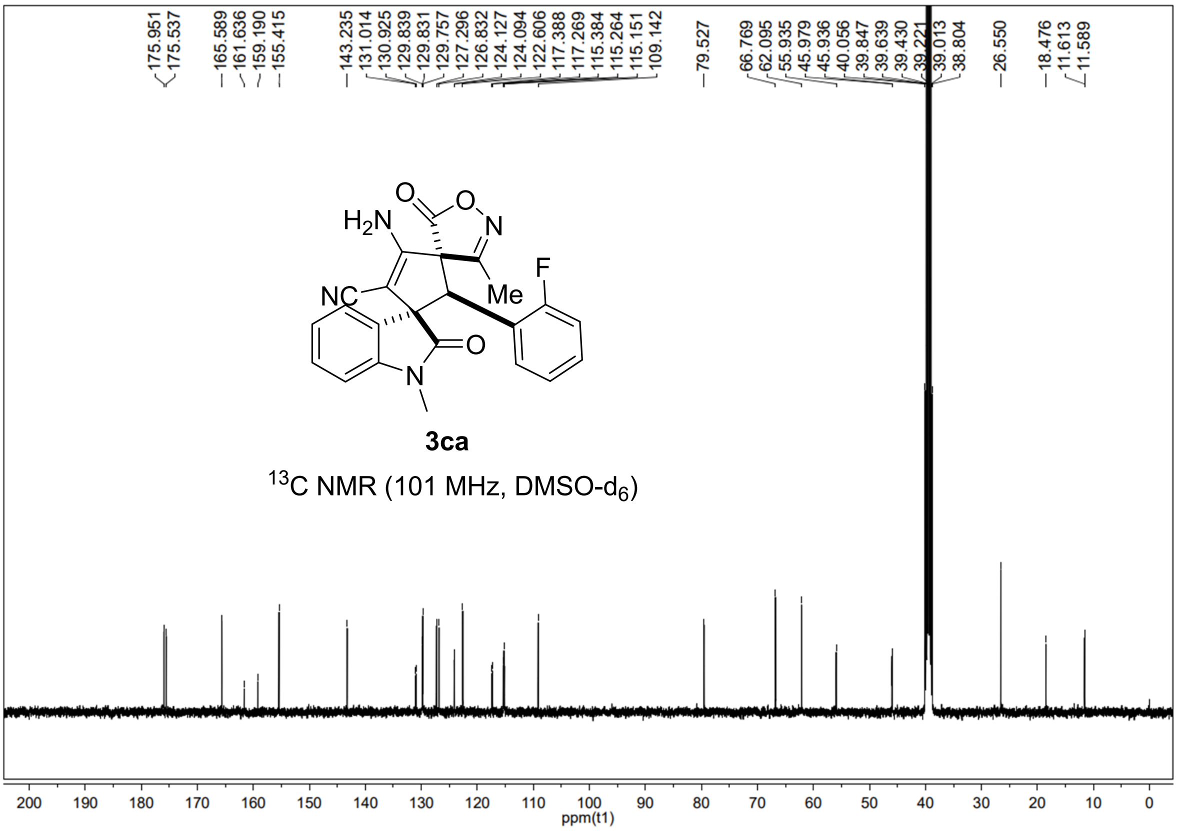
175.951
175.537
165.589
161.636
159.190
155.415
143.235
131.014
130.925
129.839
129.831
129.757
127.296
126.832
124.127
124.094
122.606
117.388
117.269
115.384
115.264
115.151
109.142
79.527
66.769
62.095
55.935
45.979
45.936
40.056
39.847
39.639
39.430
39.221
39.013
38.804
26.550
18.476
11.613
11.589
H2N
O
O
N
NC
Me
F
O
N
3ca
13C NMR (101 MHz, DMSO-d6)
200
190
180
170
160
150
140
130
120
110
100
90
80
70
60
50
40
30
20
10
0
ppm(t1)

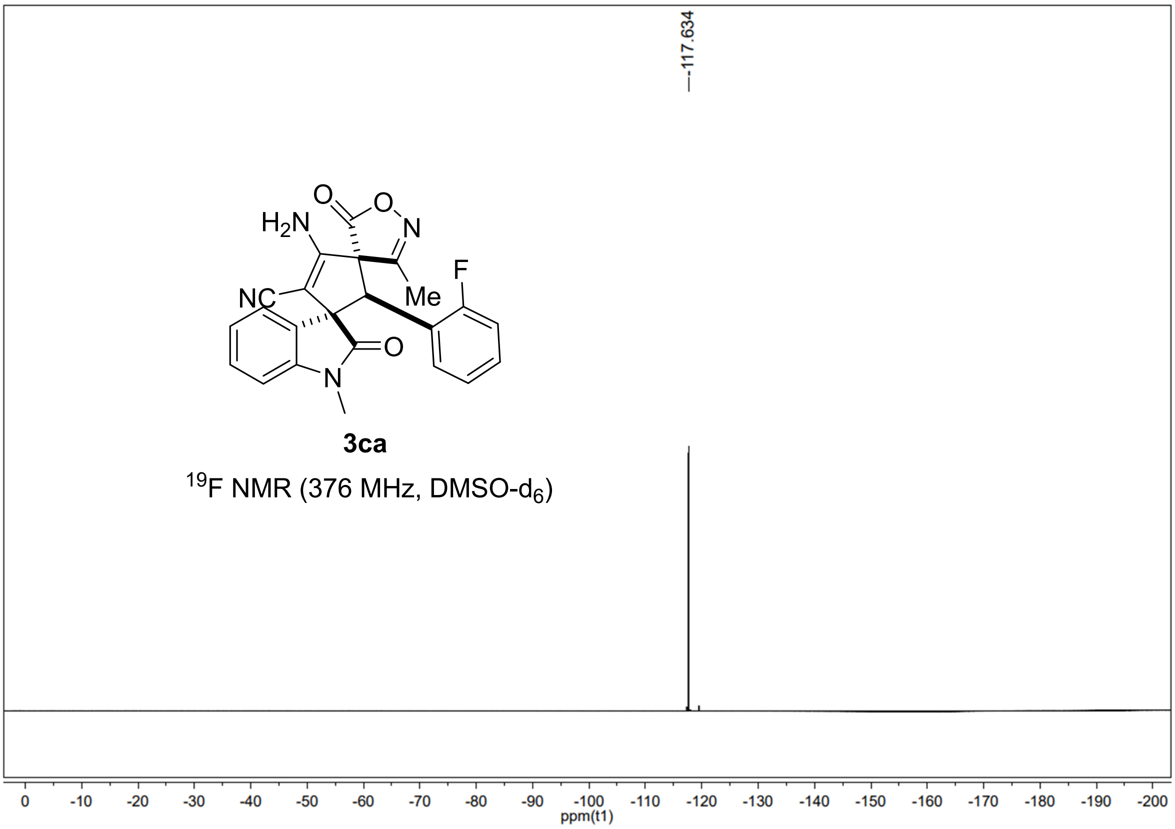
-117.634
3ca
19F NMR (376 MHz, DMSO-d6)
0 -10 -20 -30 -40 -50 -60 -70 -80 -90 -100 -110 -120 -130 -140 -150 -160 -170 -180 -190 -200
ppm(t1)

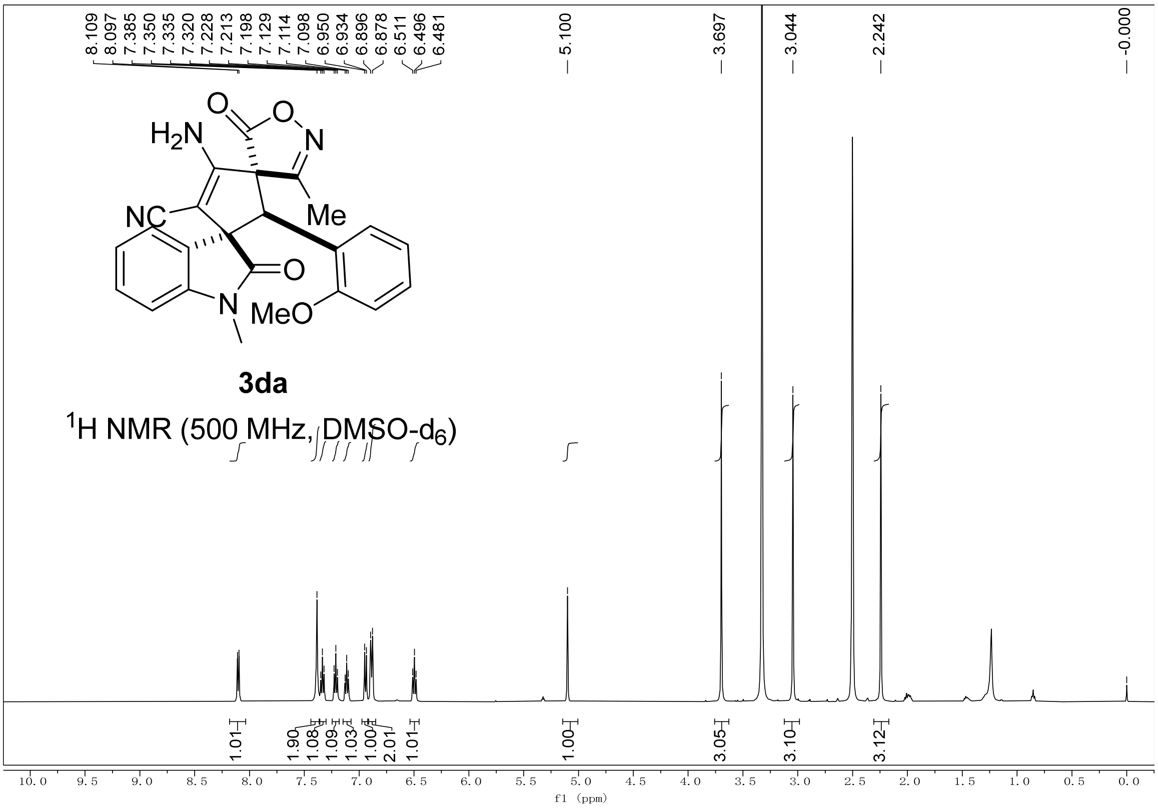
8.109 8.097 7.385 7.350 7.335 7.320 7.228 7.213 7.198 7.129 7.114 7.098 6.950 6.934 6.896 6.878 6.511 6.496 6.481
5.100
3.697
3.044
2.242
-0.000
3da
1H NMR (500 MHz, DMSO-d6)
1.01 1.90 1.08 1.09 1.03 1.00 2.01 1.01 1.00 3.05 3.10 3.12
10.0 9.5 9.0 8.5 8.0 7.5 7.0 6.5 6.0 5.5 5.0 4.5 4.0 3.5 3.0 2.5 2.0 1.5 1.0 0.5 0.0
f1 (ppm)

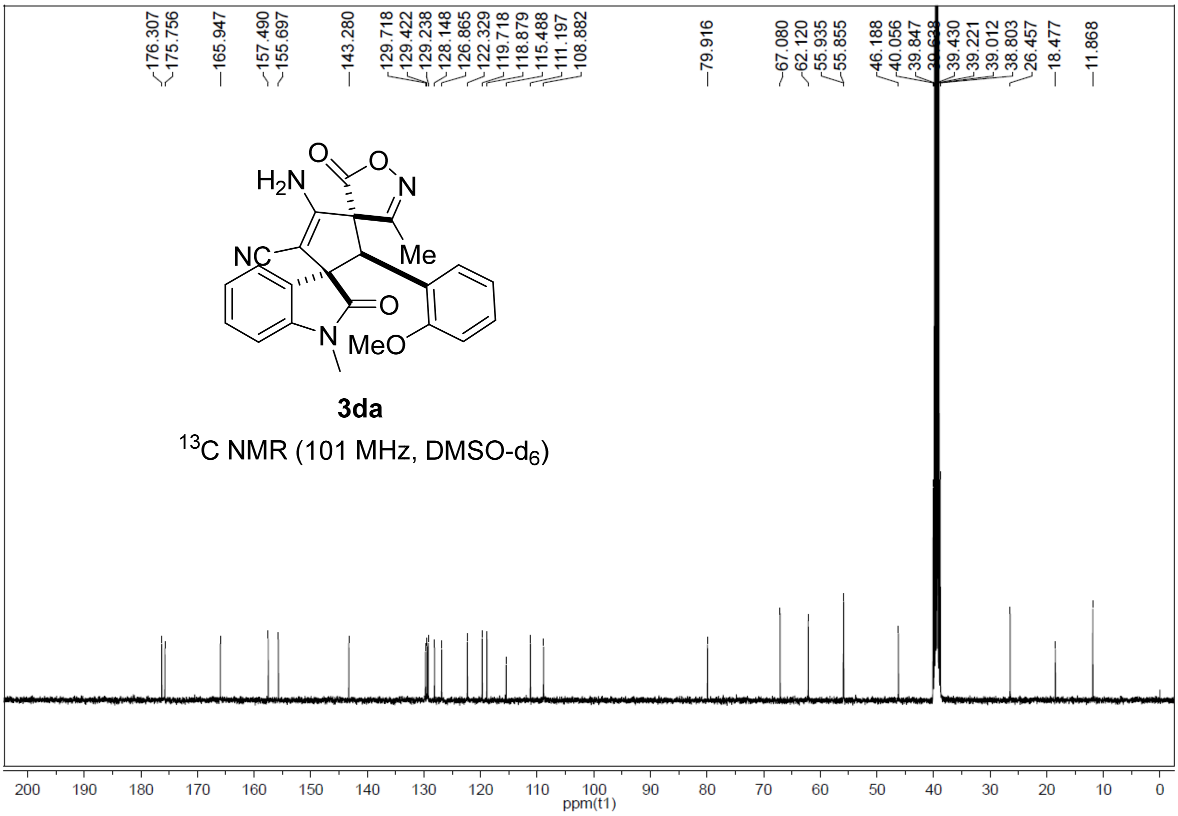

3da

^{13}C NMR (101 MHz, DMSO-d_6)

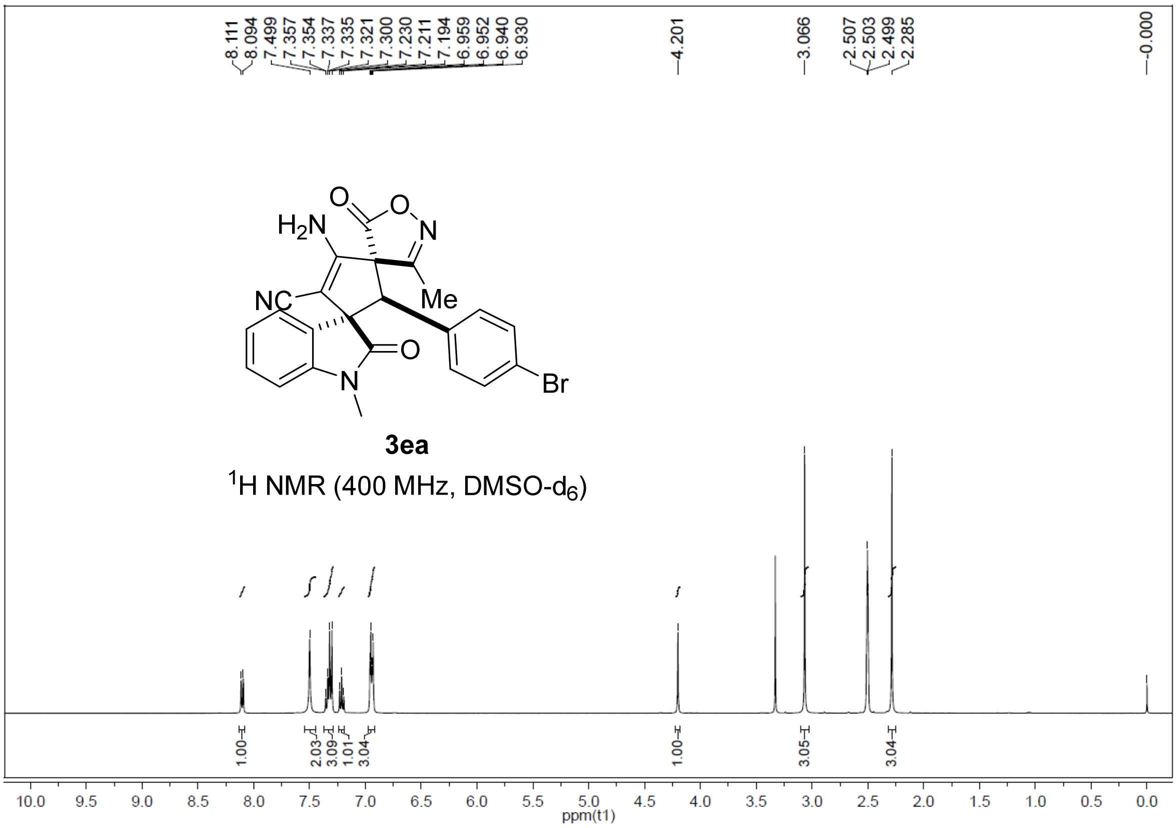

3ea

^{1}H NMR (400 MHz, DMSO-d_6)

Under the NMR acquisition conditions used, the baseline signal-to-noise ratio allows for reliable integration of signals down to approximately 5% of the major product. Since no minor diastereomer signals were observed above this detection threshold, the diastereoselectivity is conservatively reported as >20:1 (i.e., the minor isomer is below 5%). This practice is standard in organic chemistry reporting.

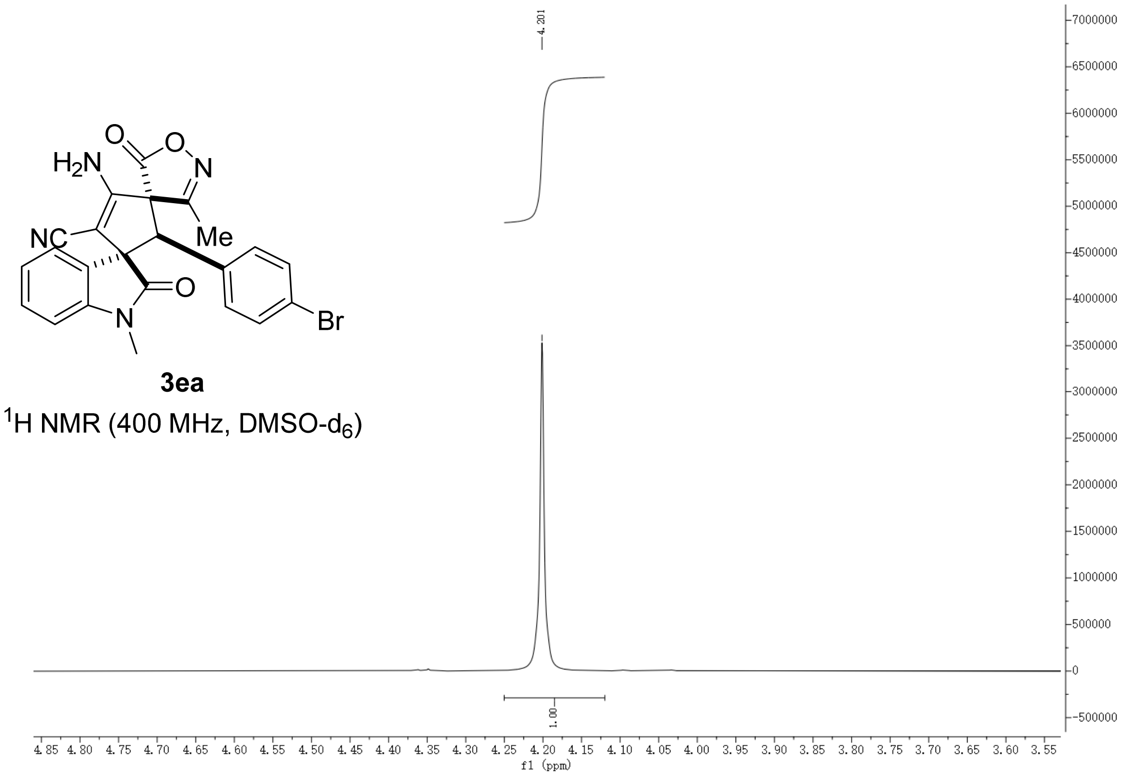

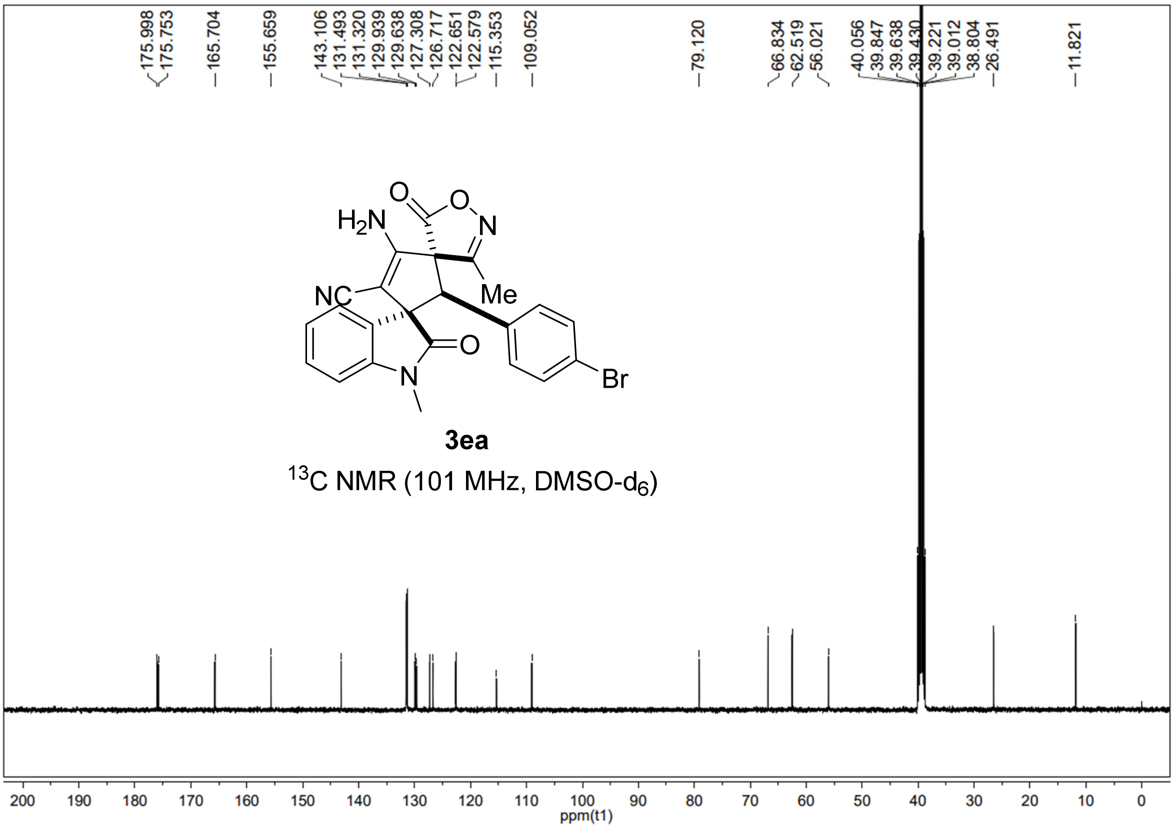

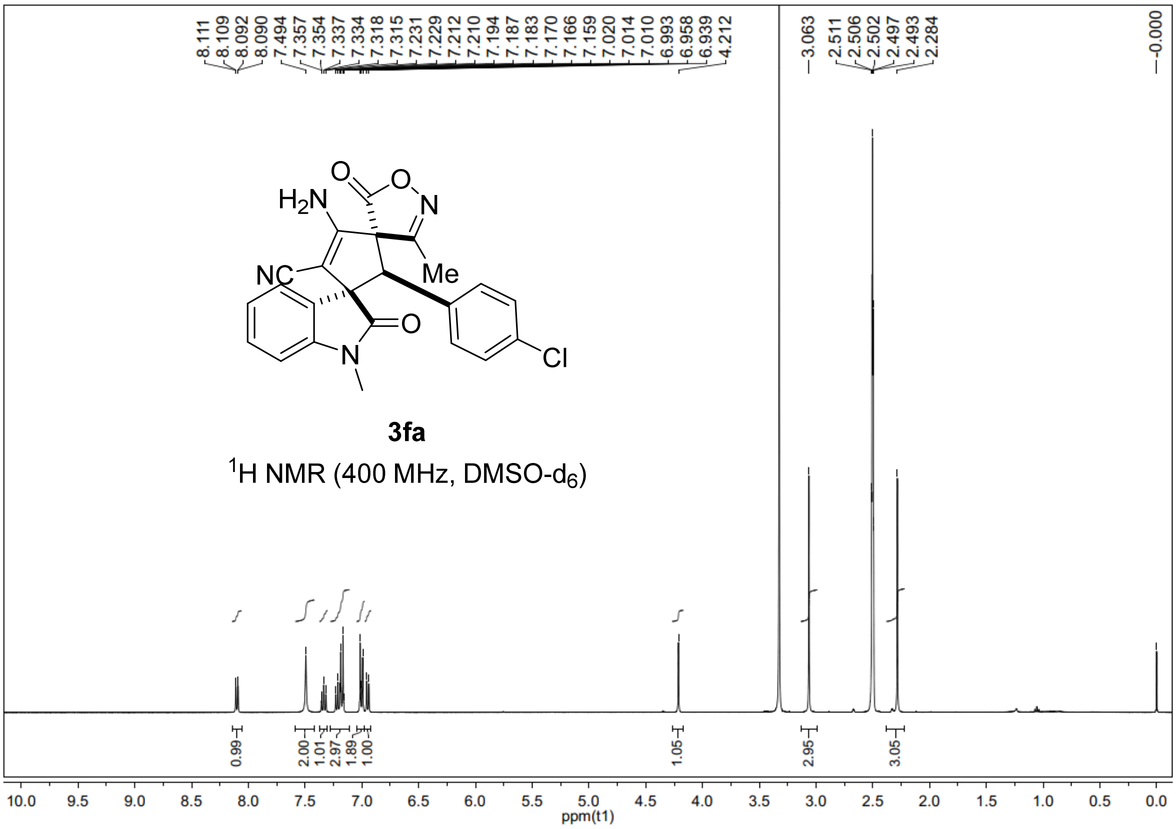
8.111
8.109
8.092
8.090
7.494
7.357
7.354
7.337
7.334
7.318
7.315
7.231
7.229
7.212
7.210
7.194
7.187
7.183
7.170
7.166
7.159
7.020
7.014
7.010
6.993
6.958
6.939
4.212
3.063
2.511
2.506
2.502
2.497
2.493
2.284
0.000
H2N
O
O
N
NC
Me
O
N
Cl
3fa
1H NMR (400 MHz, DMSO-d6)
0.99
2.00
1.01
2.97
1.89
1.00
1.05
2.95
3.05
ppm(t1)

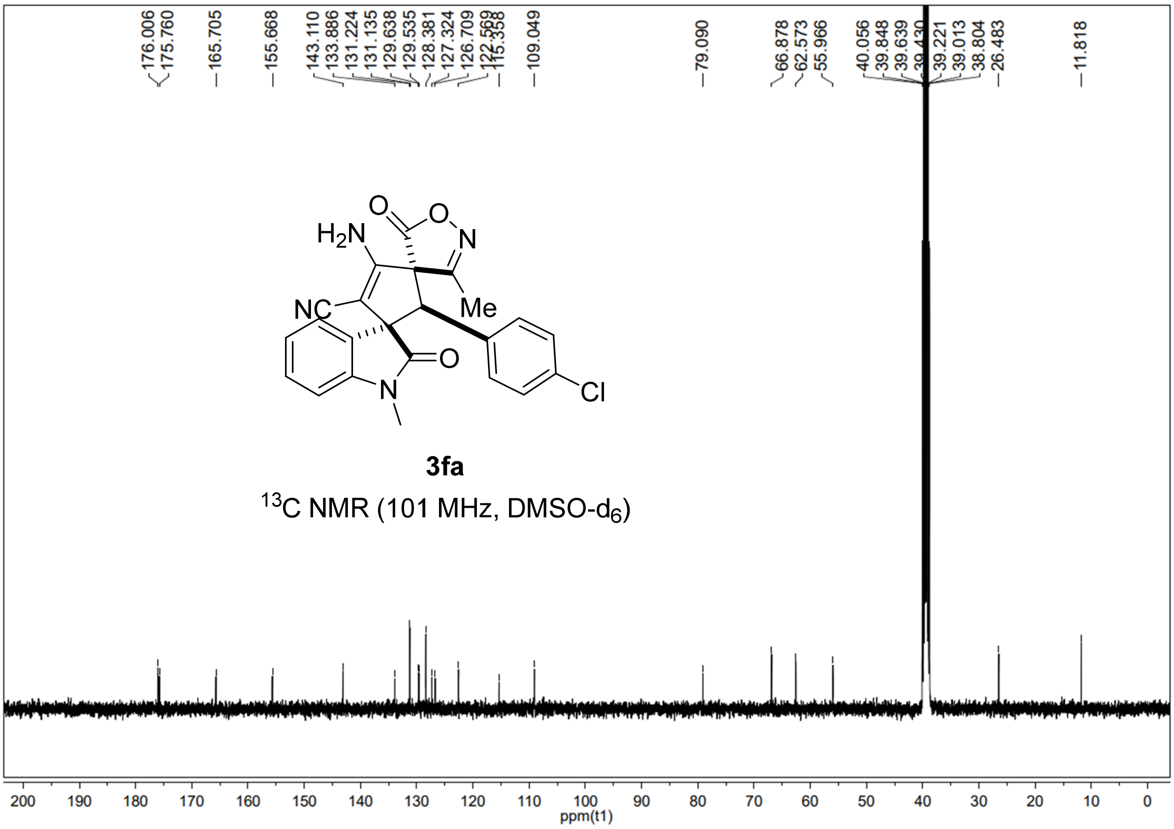
176.006
175.760
165.705
155.668
143.110
133.886
131.224
131.135
129.638
129.535
128.381
127.324
126.709
115.358
109.049
79.090
66.878
62.573
55.966
40.056
39.848
39.639
39.221
39.013
38.804
26.483
11.818
H2N
O
O
N
NC
Me
O
N
Cl
3fa
13C NMR (101 MHz, DMSO-d6)
ppm(t1)

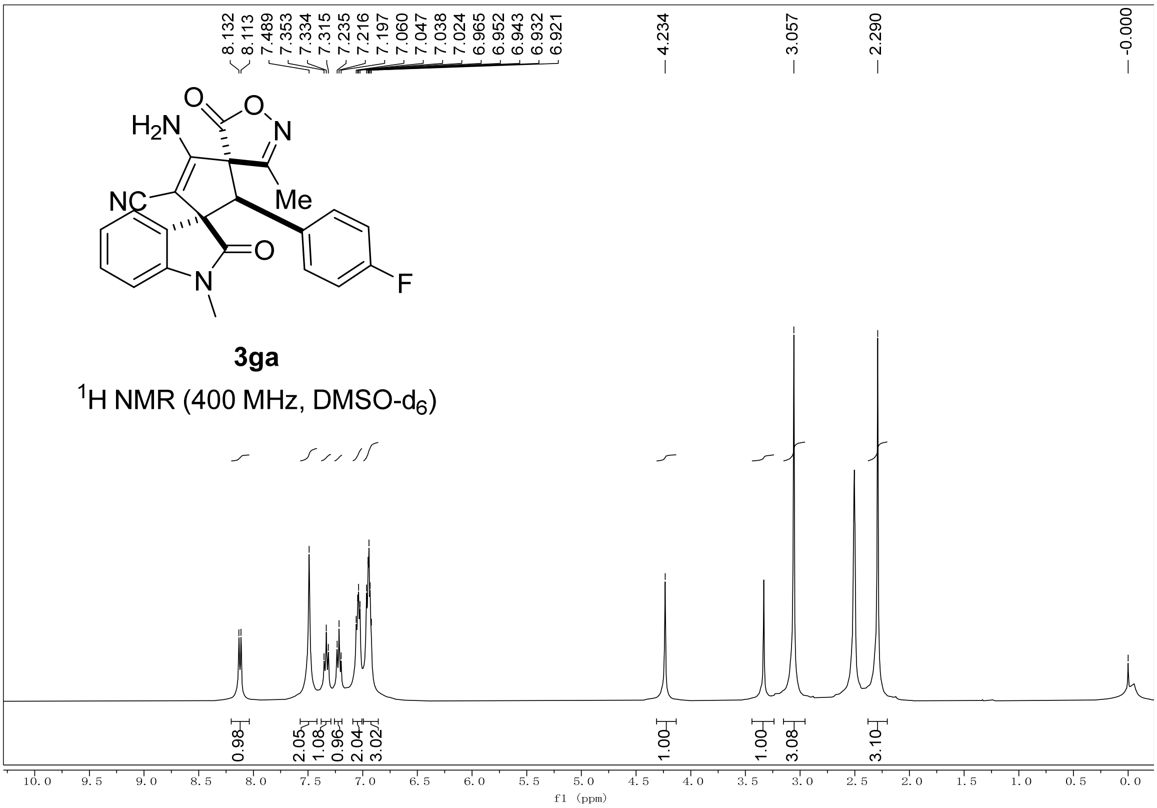
8.132
8.113
7.489
7.353
7.334
7.315
7.235
7.216
7.197
7.060
7.047
7.038
7.024
6.965
6.952
6.943
6.932
6.921
4.234
3.057
2.290
-0.000
3ga
1H NMR (400 MHz, DMSO-d6)
0.98
2.05
1.08
0.96
2.04
3.02
1.00
1.00
3.08
3.10
f1 (ppm)

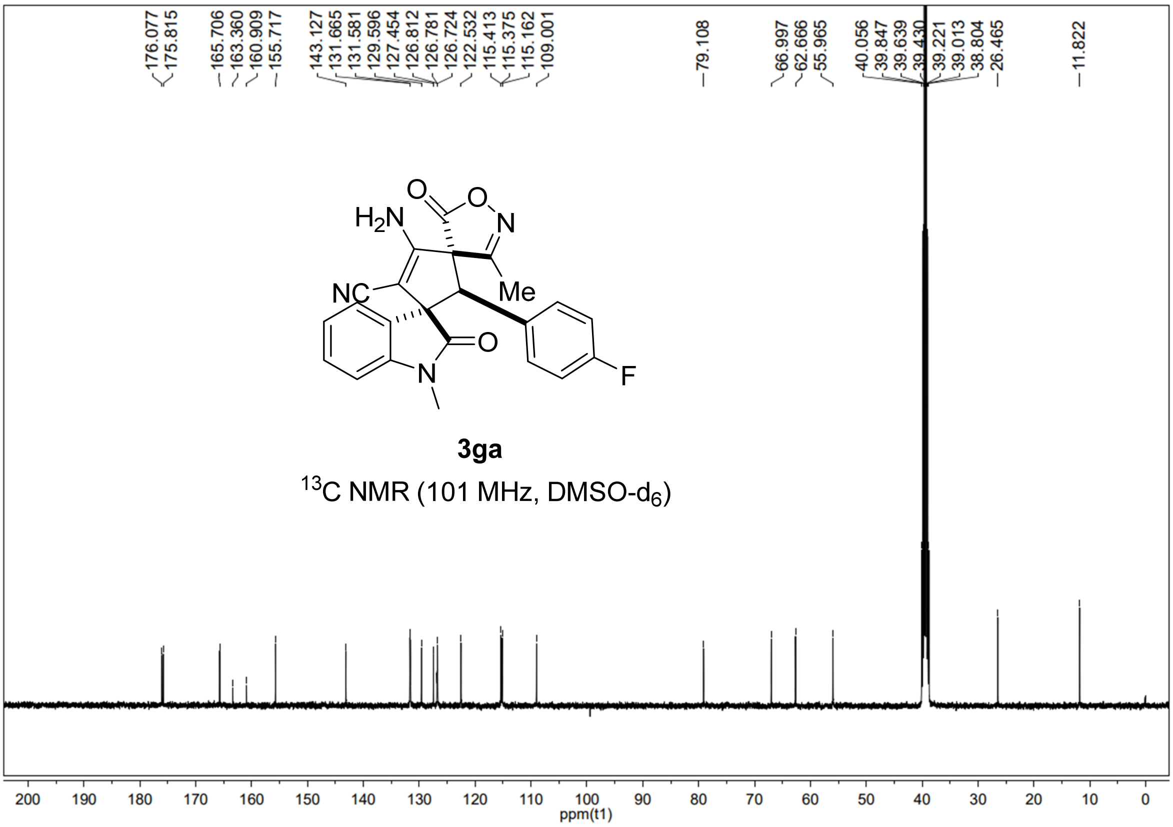
176.077
175.815
165.706
163.360
160.909
155.717
143.127
131.665
131.581
129.596
127.454
126.812
126.781
126.724
122.532
115.413
115.375
115.162
109.001
79.108
66.997
62.666
55.965
40.056
39.847
39.639
39.430
39.221
39.013
38.804
26.465
11.822
3ga
13C NMR (101 MHz, DMSO-d6)
ppm(t1)

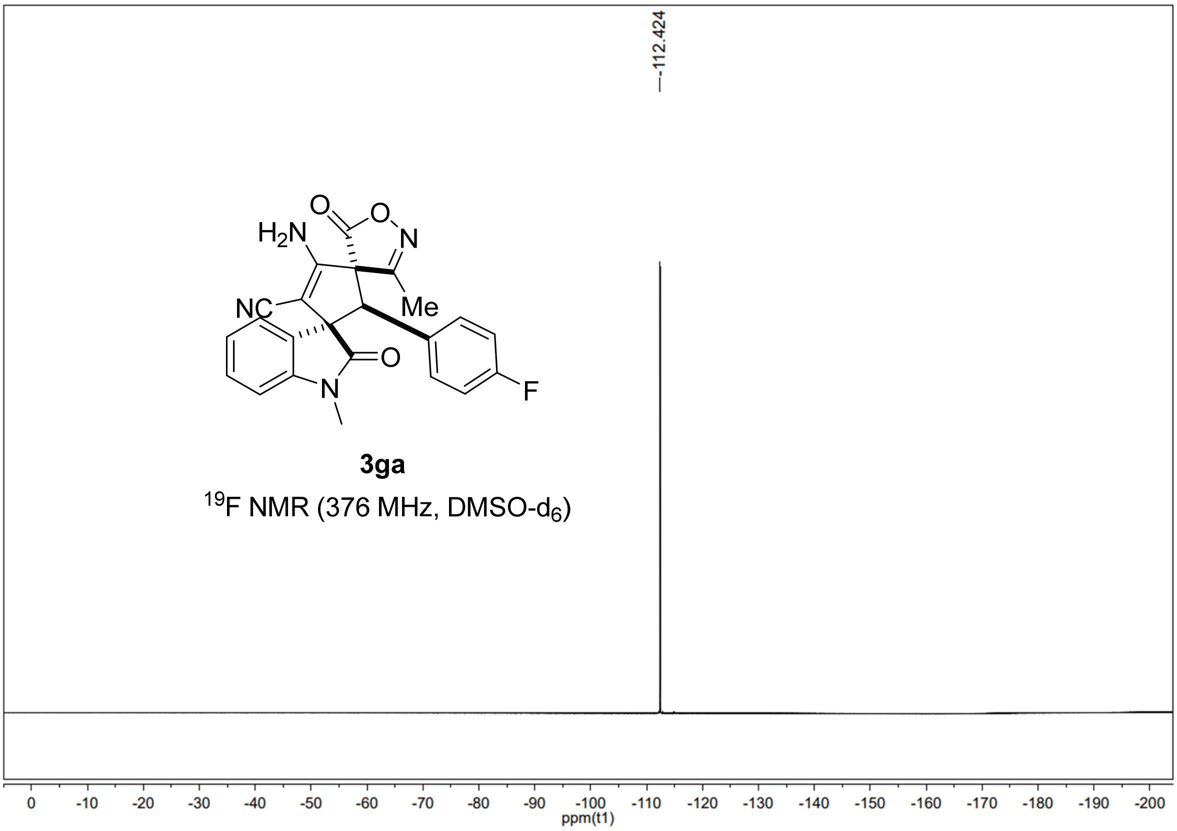
-112.424
H2N
O
O
N
NC
Me
O
N
F
3ga
19F NMR (376 MHz, DMSO-d6)
0 -10 -20 -30 -40 -50 -60 -70 -80 -90 -100 -110 -120 -130 -140 -150 -160 -170 -180 -190 -200
ppm(t1)

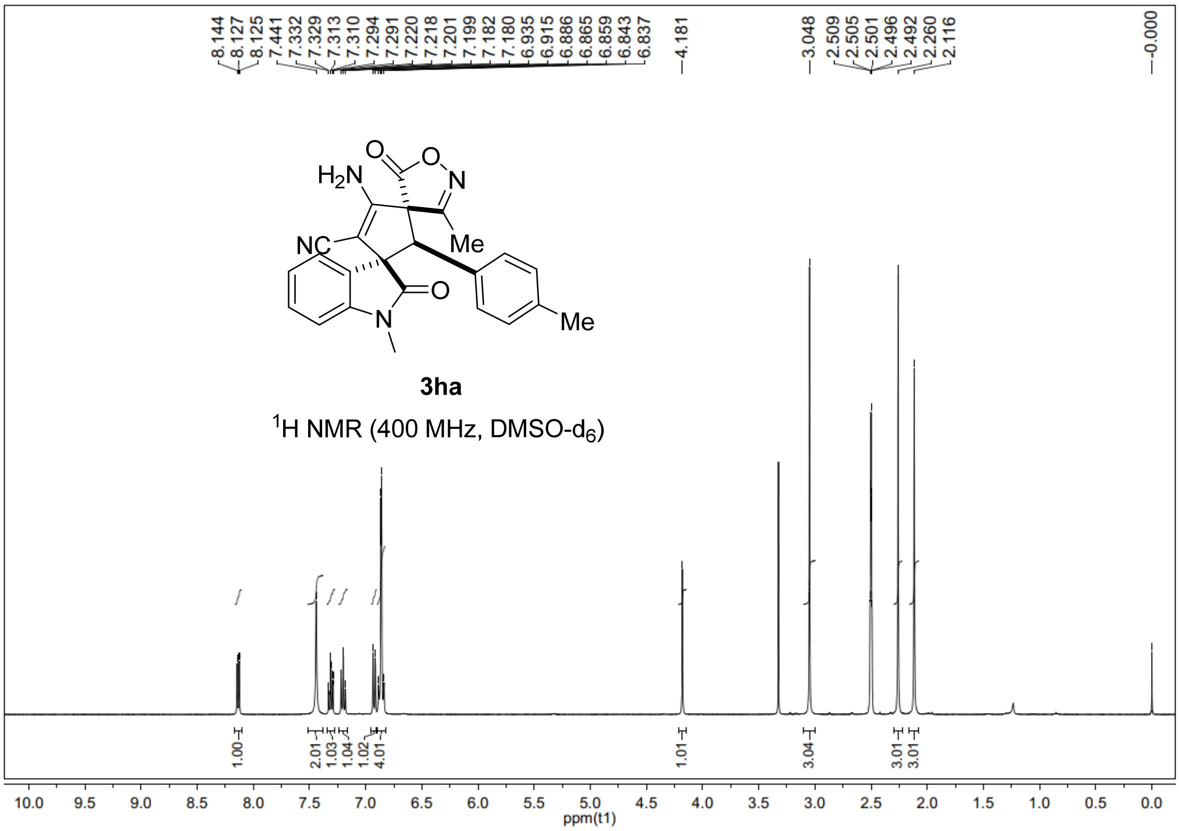
8.144 8.127 8.125 7.441 7.332 7.329 7.313 7.310 7.294 7.291 7.220 7.218 7.201 7.199 7.182 7.180 6.935 6.915 6.886 6.865 6.859 6.843 6.837
4.181
3.048 2.509 2.505 2.501 2.496 2.492 2.260 2.116
-0.000
H2N
O
O
N
NC
Me
O
N
Me
3ha
1H NMR (400 MHz, DMSO-d6)
1.00
2.01 1.03 1.04 1.02 4.01
1.01
3.04
3.01 3.01
10.0 9.5 9.0 8.5 8.0 7.5 7.0 6.5 6.0 5.5 5.0 4.5 4.0 3.5 3.0 2.5 2.0 1.5 1.0 0.5 0.0
ppm(t1)

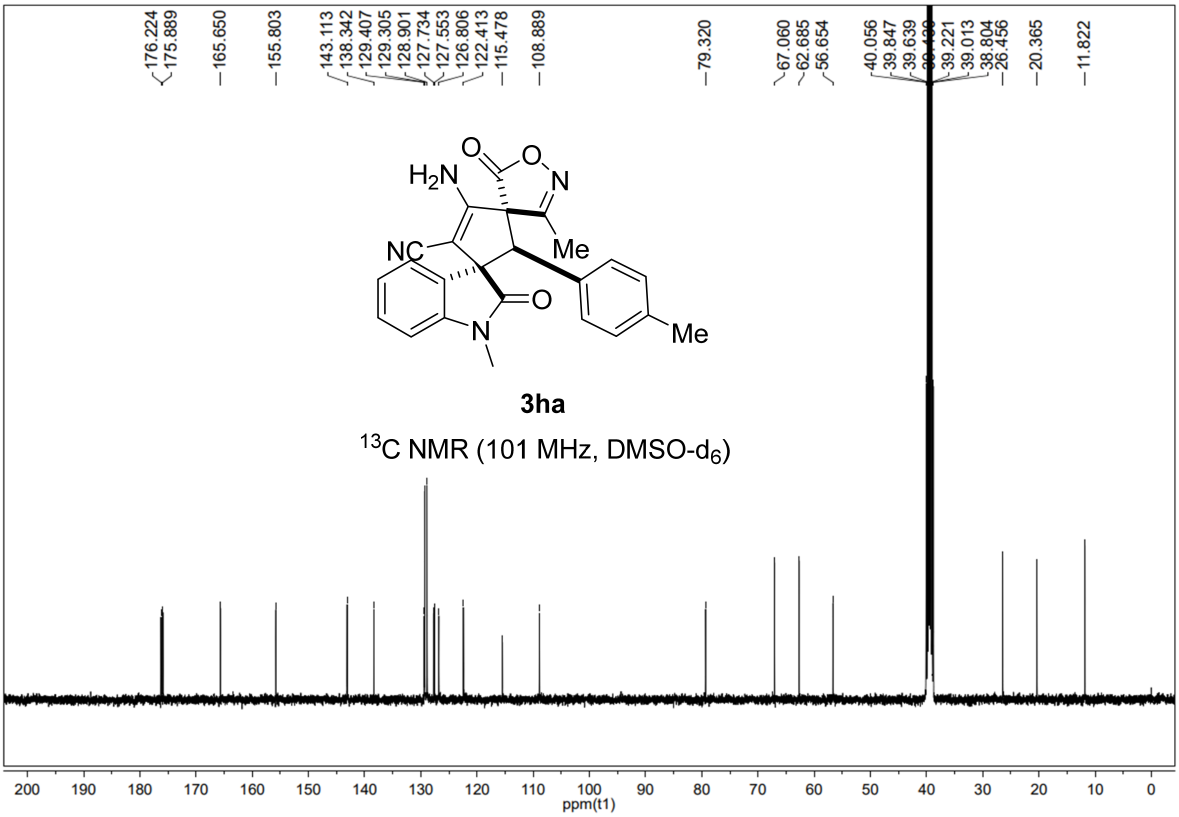
176.224
175.889
165.650
155.803
143.113
138.342
129.407
129.305
128.901
127.734
127.553
126.806
122.413
115.478
108.889
79.320
67.060
62.685
56.654
40.056
39.847
39.639
39.221
39.013
38.804
26.456
20.365
11.822
H2N
O
O
N
Me
NC
O
N
Me
3ha
13C NMR (101 MHz, DMSO-d6)
200 190 180 170 160 150 140 130 120 110 100 90 80 70 60 50 40 30 20 10 0
ppm(t1)

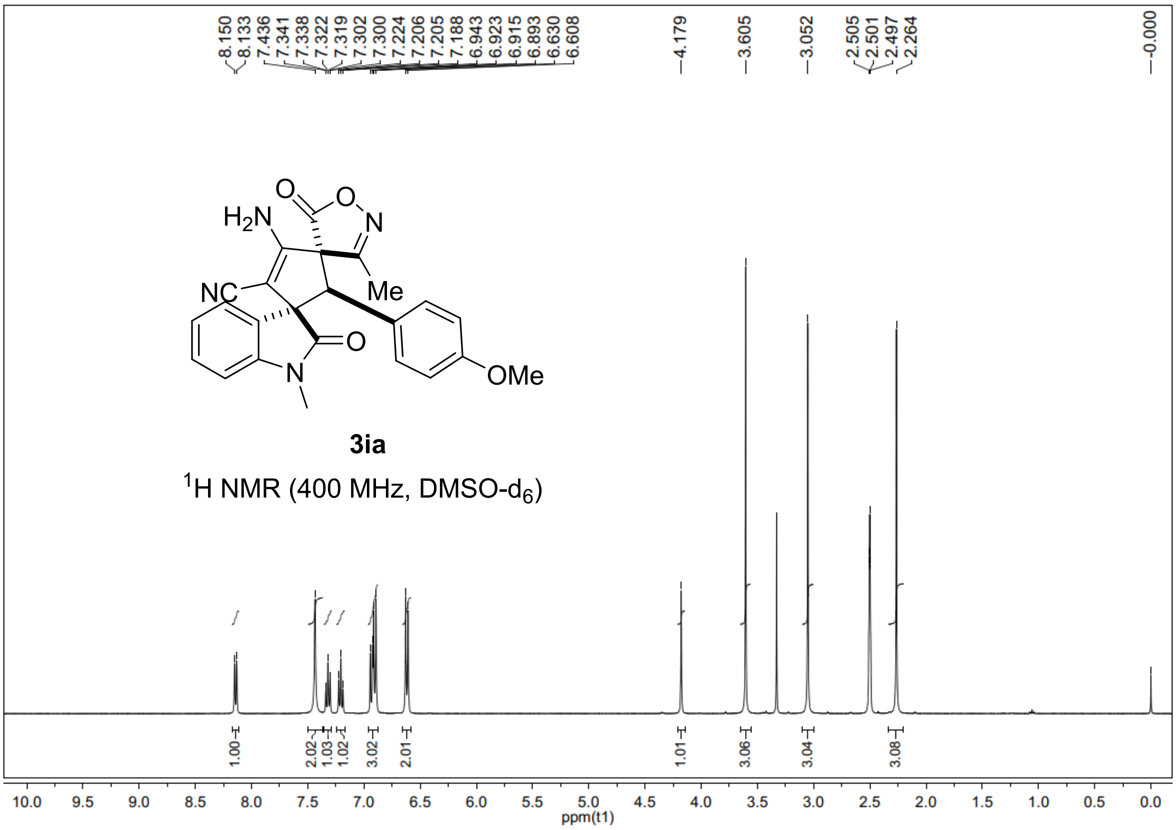
8.150
8.133
7.436
7.341
7.338
7.322
7.319
7.302
7.300
7.224
7.206
7.205
7.188
6.943
6.923
6.915
6.893
6.630
6.608
4.179
3.605
3.052
2.505
2.501
2.497
2.264
-0.000
H2N
O
O
N
Me
NC
O
N
OMe
3ia
1H NMR (400 MHz, DMSO-d6)
1.00
2.02
1.03
1.02
3.02
2.01
1.01
3.06
3.04
3.08
10.0 9.5 9.0 8.5 8.0 7.5 7.0 6.5 6.0 5.5 5.0 4.5 4.0 3.5 3.0 2.5 2.0 1.5 1.0 0.5 0.0
ppm(t1)

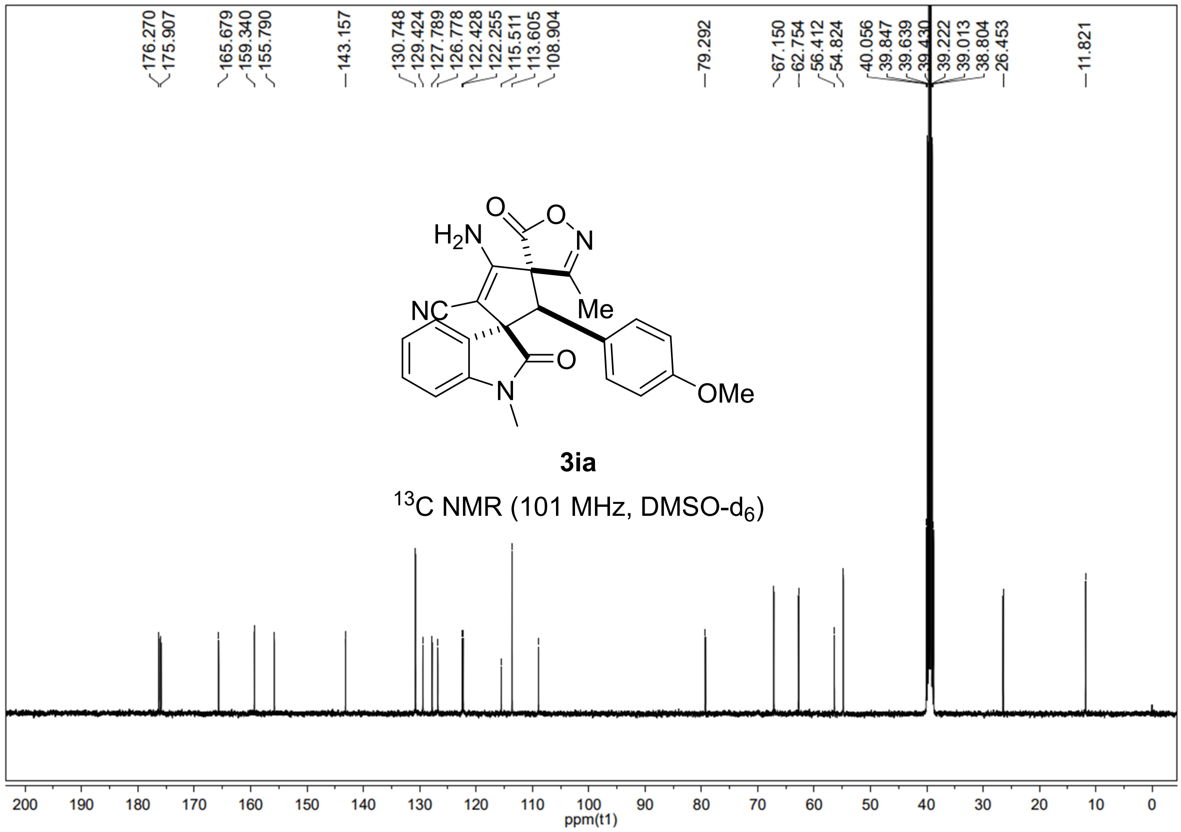
176.270
175.907
165.679
159.340
155.790
143.157
130.748
129.424
127.789
126.778
122.428
122.255
115.511
113.605
108.904
79.292
67.150
62.754
56.412
54.824
40.056
39.847
39.639
39.430
39.222
39.013
38.804
26.453
11.821
H2N
O
O
N
Me
NC
O
N
OMe
3ia
13C NMR (101 MHz, DMSO-d6)
ppm(t1)

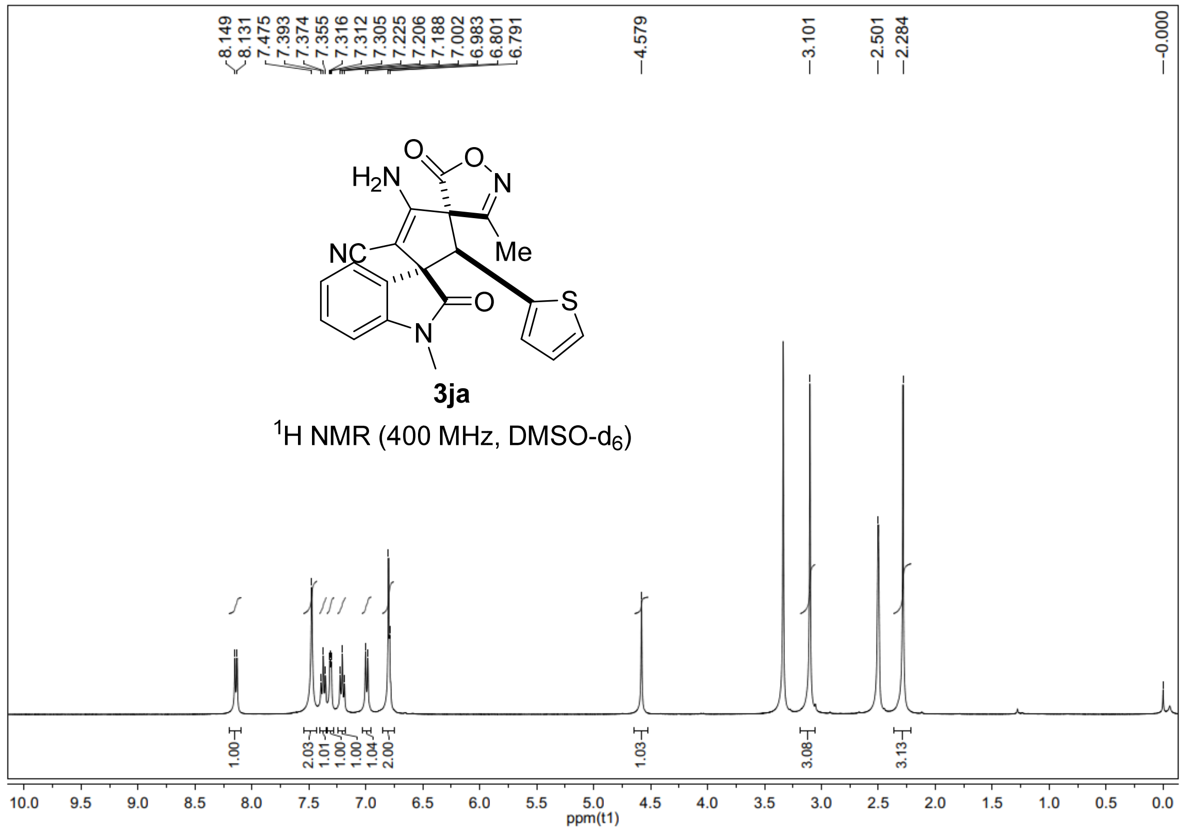
8.149
8.131
7.475
7.393
7.374
7.355
7.316
7.312
7.305
7.225
7.206
7.188
7.002
6.983
6.801
6.791
4.579
3.101
2.501
2.284
-0.000
H2N
O
O
N
Me
NC
S
O
N
3ja
1H NMR (400 MHz, DMSO-d6)
1.00
2.03
1.01
1.00
1.00
1.04
2.00
1.03
3.08
3.13
ppm(t1)

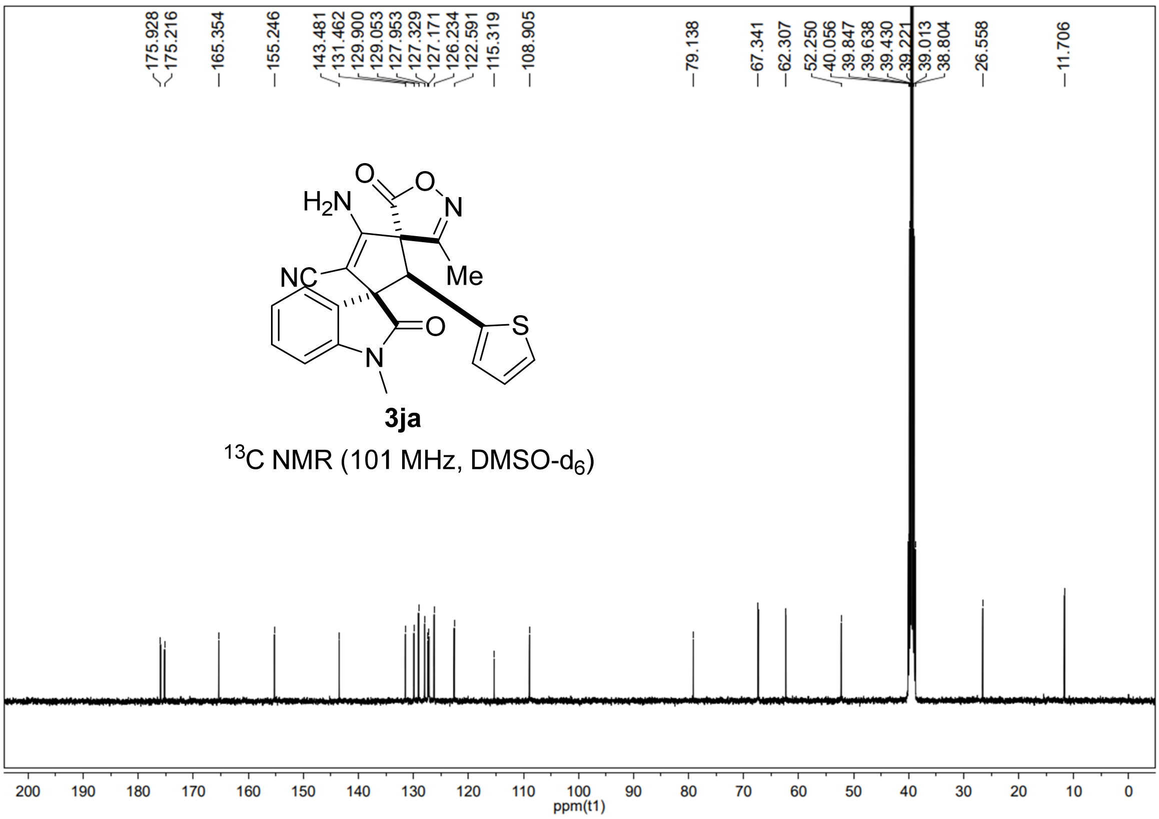
175.928
175.216
165.354
155.246
143.481
131.462
129.900
129.053
127.953
127.329
127.171
126.234
122.591
115.319
108.905
79.138
67.341
62.307
52.250
40.056
39.847
39.638
39.430
39.221
39.013
38.804
26.558
11.706
3ja
^{13}C NMR (101 MHz, DMSO-d_6)
ppm(t1)

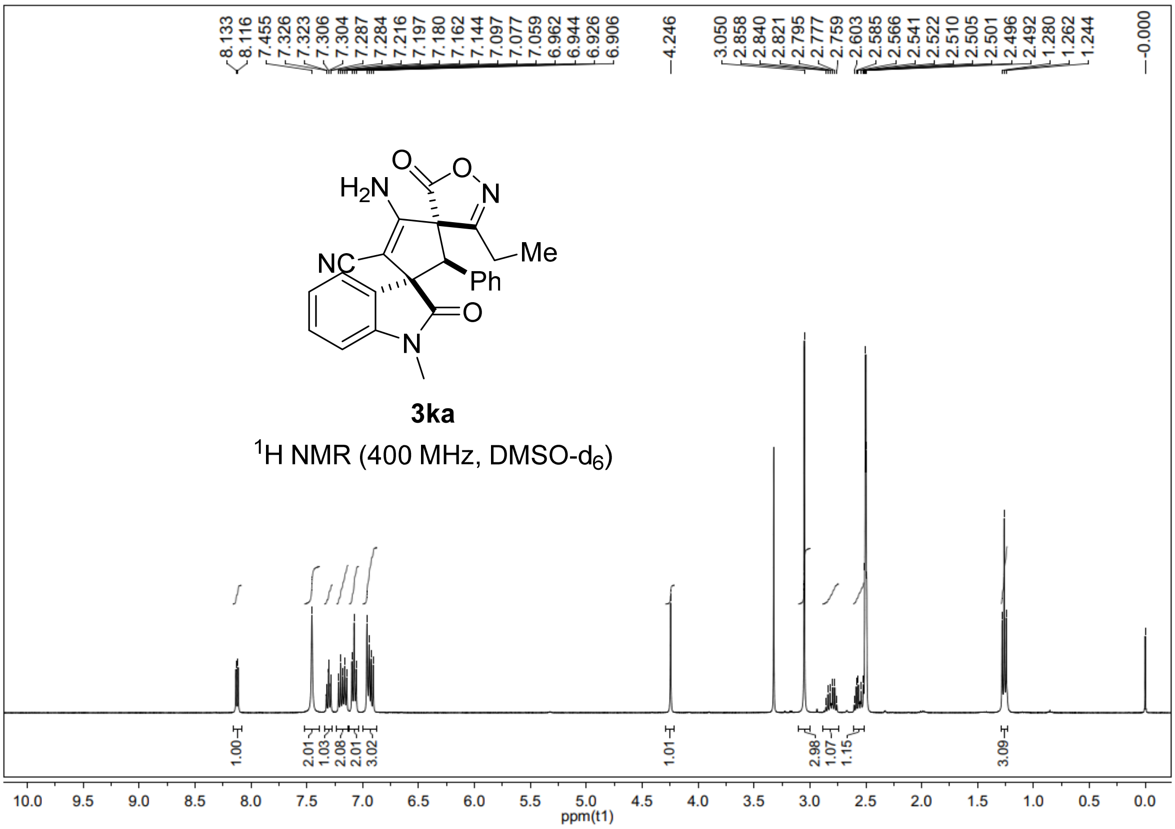
8.133
8.116
7.455
7.326
7.323
7.306
7.304
7.287
7.284
7.216
7.197
7.180
7.162
7.144
7.097
7.077
7.059
6.962
6.944
6.926
6.906
4.246
3.050
2.858
2.840
2.821
2.795
2.777
2.759
2.603
2.585
2.566
2.541
2.522
2.510
2.505
2.501
2.496
2.492
1.280
1.262
1.244
0.000
1.00
2.01
1.03
2.08
2.01
3.02
1.01
2.98
1.07
1.15
3.09
3ka
^{1}H NMR (400 MHz, DMSO-d_6)
ppm(t1)

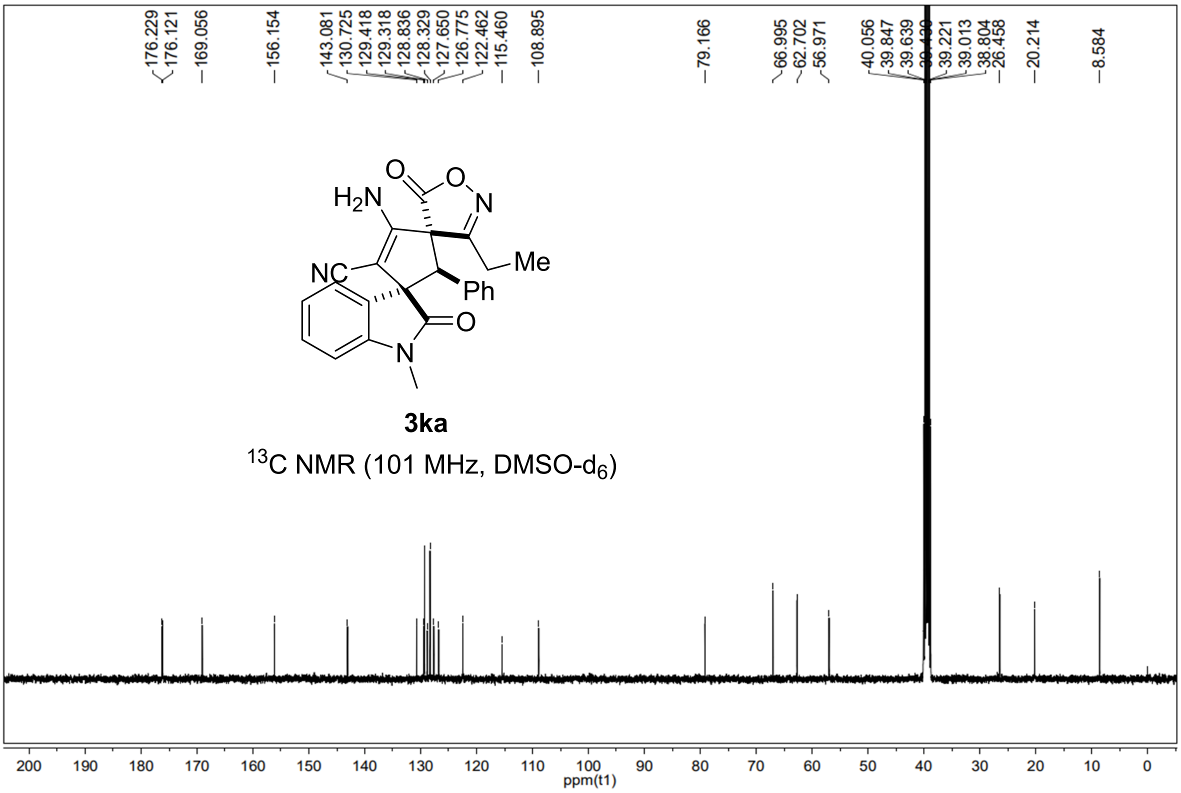
176.229
176.121
169.056
156.154
143.081
130.725
129.418
129.318
128.836
128.329
127.650
126.775
122.462
115.460
108.895
79.166
66.995
62.702
56.971
40.056
39.847
39.639
39.221
39.013
38.804
26.458
20.214
8.584
H_2N
O
O
N
Me
NC
Ph
O
N
3ka
^{13}C NMR (101 MHz, DMSO-d_6)
200 190 180 170 160 150 140 130 120 110 100 90 80 70 60 50 40 30 20 10 0
ppm(t1)

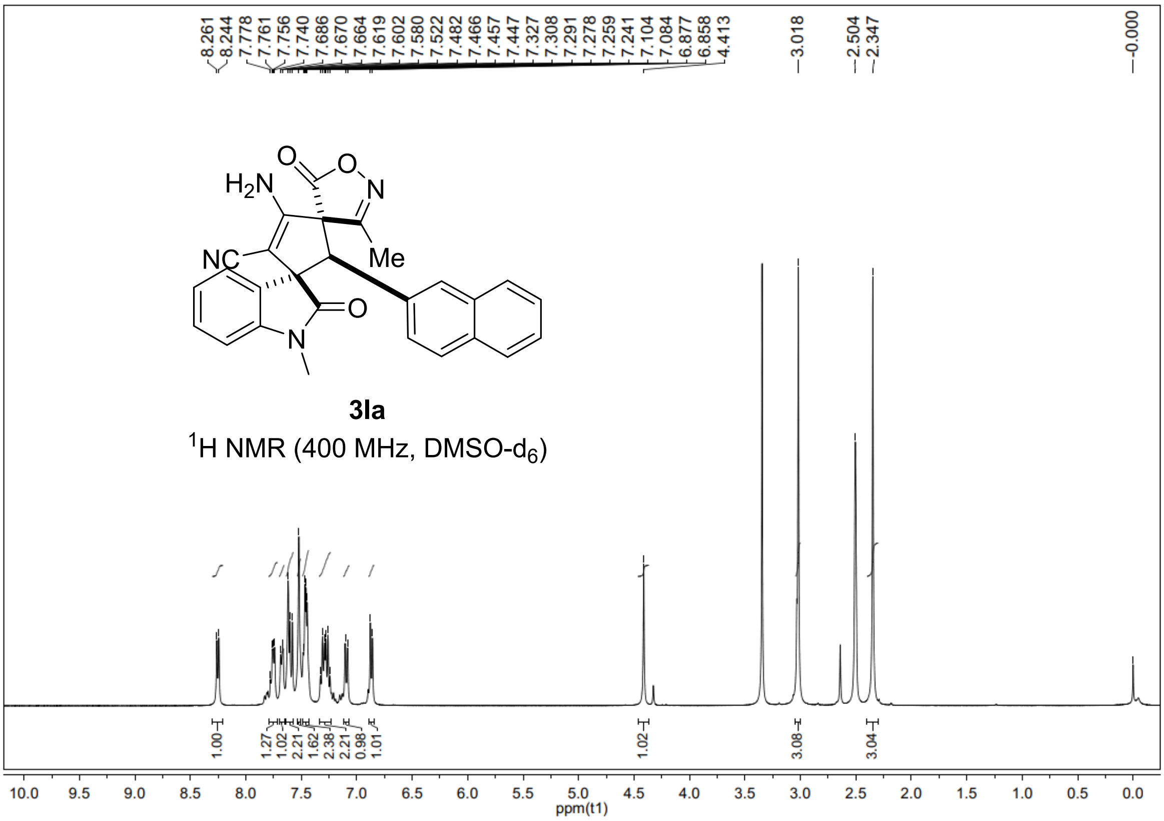
8.261
8.244
7.778
7.761
7.756
7.740
7.686
7.670
7.664
7.619
7.602
7.580
7.522
7.482
7.466
7.457
7.447
7.327
7.308
7.291
7.278
7.259
7.241
7.104
7.084
6.877
6.858
4.413
3.018
2.504
2.347
-0.000
H_2N
O
O
N
NC
Me
O
N
3la
^1H NMR (400 MHz, DMSO-d_6)
1.00
1.27
1.02
2.21
1.62
2.38
2.21
0.98
1.01
1.02
3.08
3.04
10.0 9.5 9.0 8.5 8.0 7.5 7.0 6.5 6.0 5.5 5.0 4.5 4.0 3.5 3.0 2.5 2.0 1.5 1.0 0.5 0.0
ppm(t1)

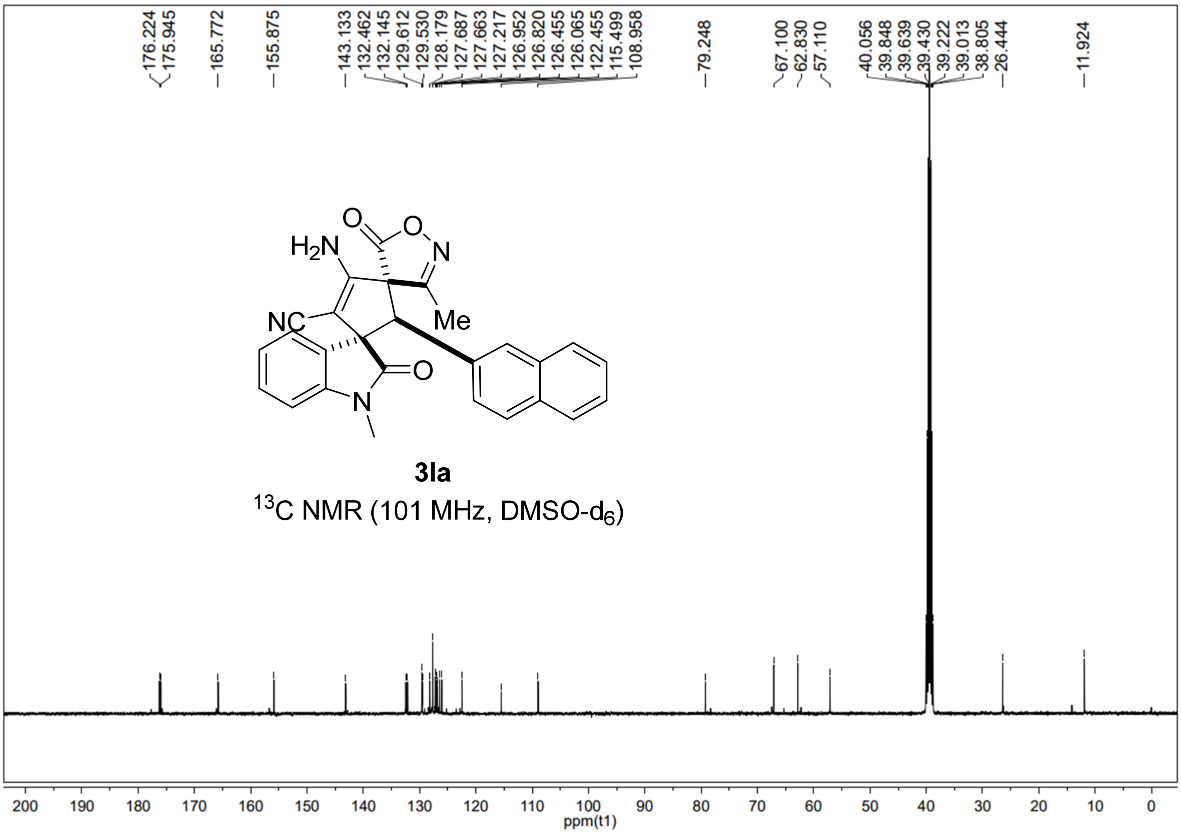
176.224
175.945
165.772
155.875
143.133
132.462
132.145
129.612
129.530
128.179
127.687
127.663
127.217
126.952
126.820
126.455
126.065
122.455
115.499
108.958
79.248
67.100
62.830
57.110
40.056
39.848
39.639
39.430
39.222
39.013
38.805
26.444
11.924
H2N
O
O
N
NC
Me
O
N
3la
13C NMR (101 MHz, DMSO-d6)
ppm(t1)

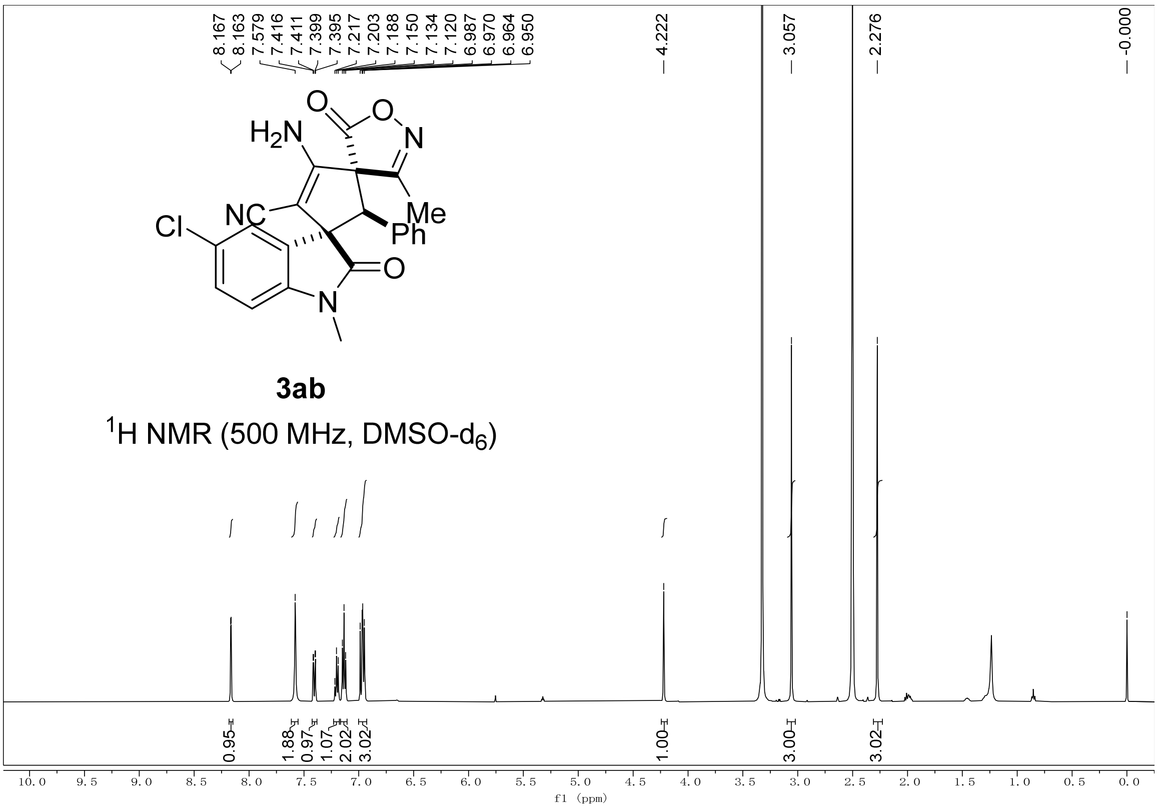
8.167
8.163
7.579
7.416
7.411
7.399
7.395
7.217
7.203
7.188
7.150
7.134
7.120
6.987
6.970
6.964
6.950
4.222
3.057
2.276
-0.000
H2N
O
O
N
Cl
NC
Me
Ph
O
N
3ab
1H NMR (500 MHz, DMSO-d6)
0.95
1.88
0.97
1.07
2.02
3.02
1.00
3.00
3.02
f1 (ppm)

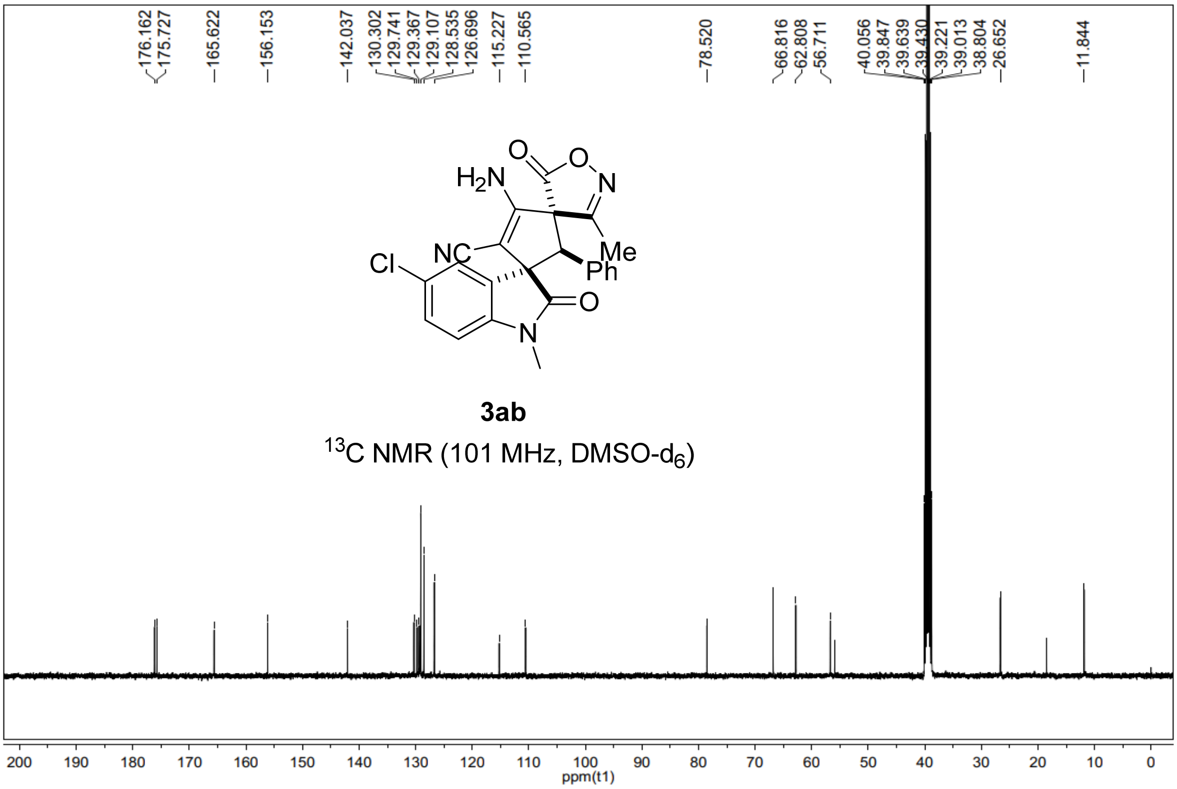
176.162
175.727
165.622
156.153
142.037
130.302
129.741
129.367
129.107
128.535
126.696
115.227
110.565
78.520
66.816
62.808
56.711
40.056
39.847
39.639
39.430
39.221
39.013
38.804
26.652
11.844
3ab
13C NMR (101 MHz, DMSO-d6)
ppm(t1)

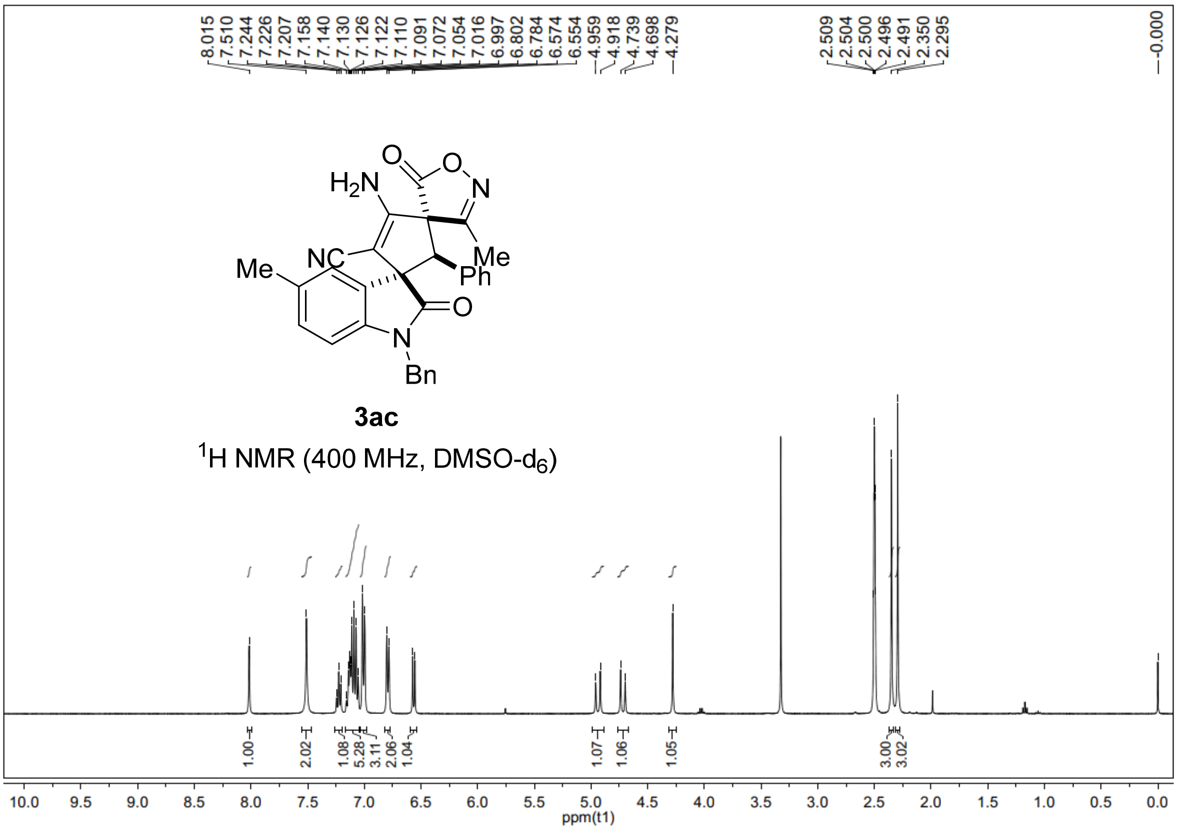
8.015
7.510
7.244
7.226
7.207
7.158
7.140
7.130
7.126
7.122
7.110
7.091
7.072
7.054
7.016
6.997
6.802
6.784
6.574
6.554
4.959
4.918
4.739
4.698
4.279
2.509
2.504
2.500
2.496
2.491
2.350
2.295
-0.000
3ac
1H NMR (400 MHz, DMSO-d6)
1.00
2.02
1.08
5.28
3.11
2.06
1.04
1.07
1.06
1.05
3.00
3.02
ppm(t1)

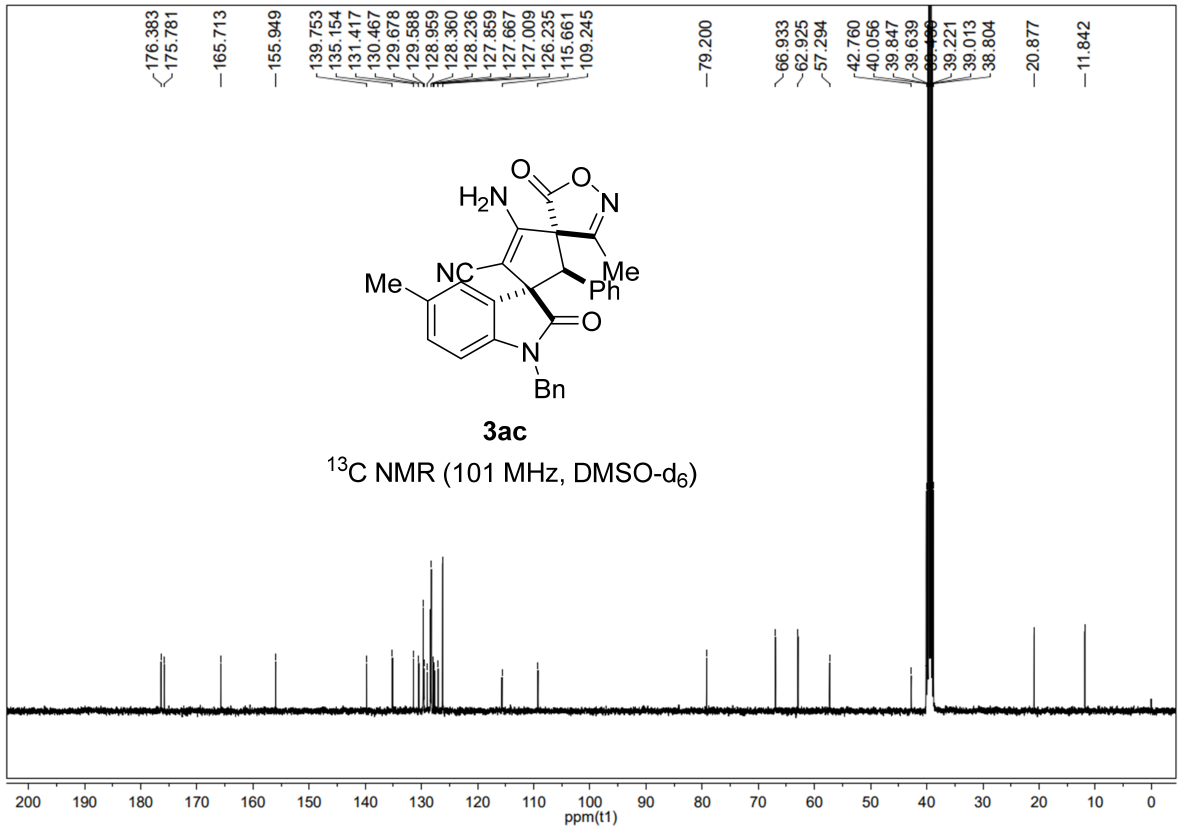

3ac

^{13}C NMR (101 MHz, DMSO-d$_6$)

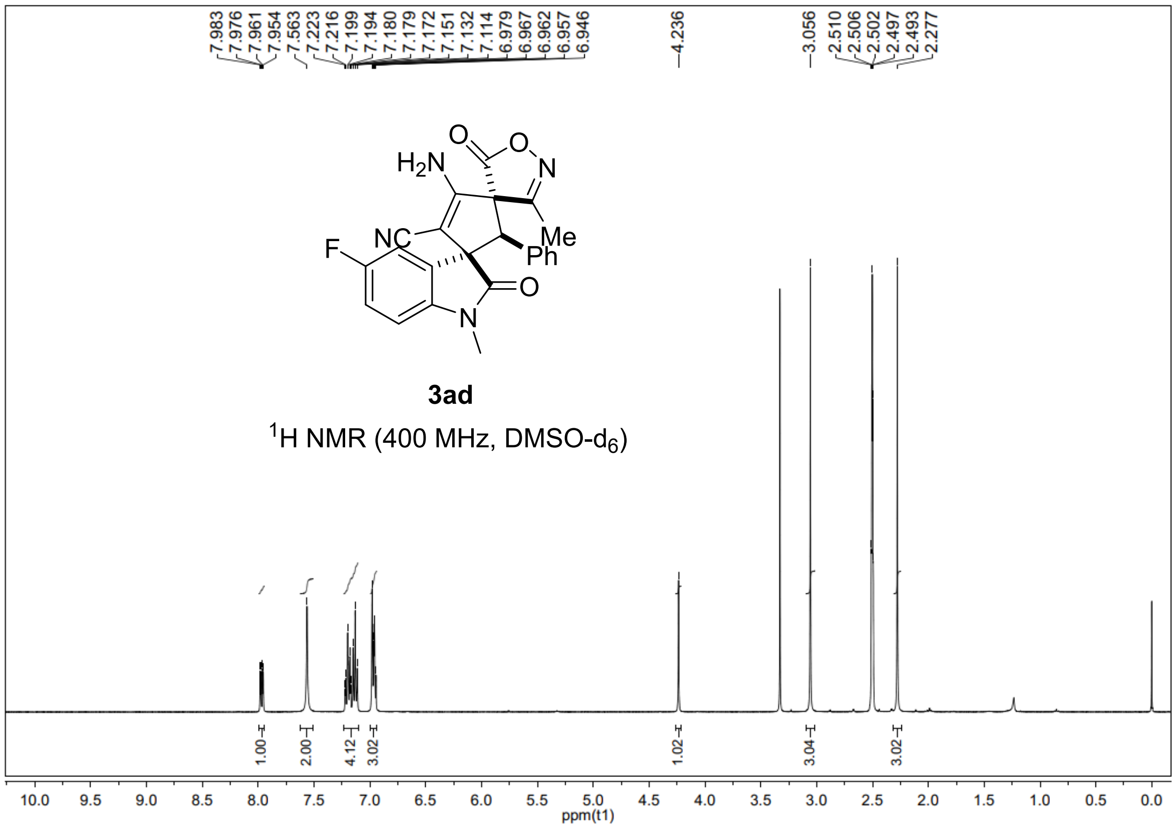

3ad

^{1}H NMR (400 MHz, DMSO-d$_6$)

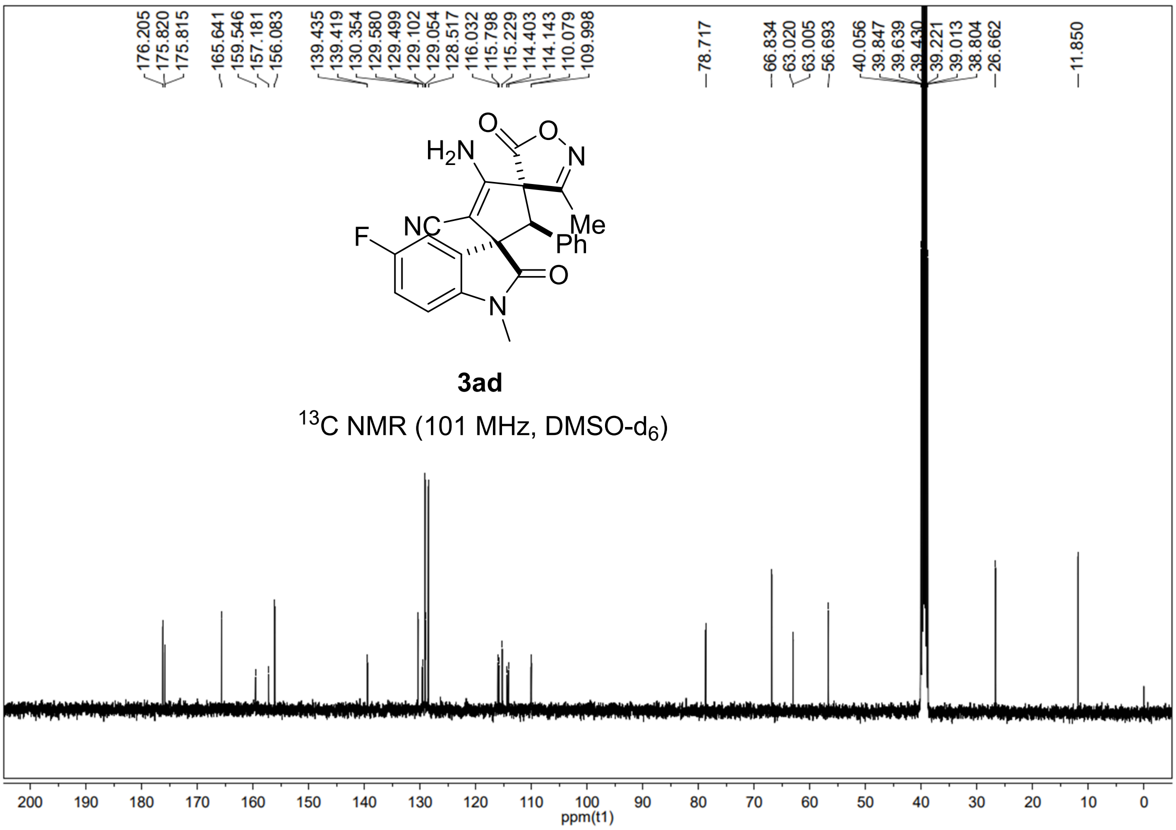
176.205
175.820
175.815
165.641
159.546
157.181
156.083
139.435
139.419
130.354
129.580
129.499
129.102
129.054
128.517
116.032
115.798
115.229
114.403
114.143
110.079
109.998
78.717
66.834
63.020
63.005
56.693
40.056
39.847
39.639
39.430
39.221
39.013
38.804
26.662
11.850
H2N
O
O
N
NC
Me
F
Ph
O
N
3ad
13C NMR (101 MHz, DMSO-d6)
ppm(t1)

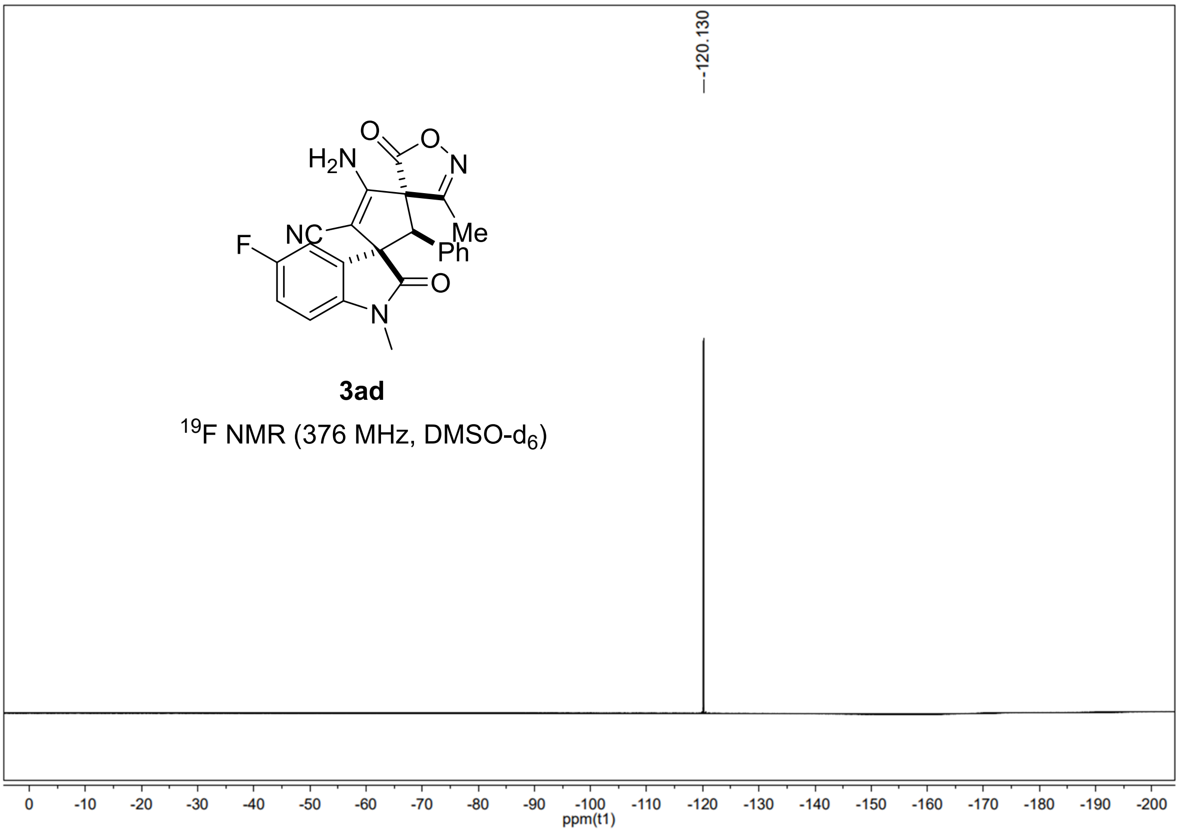
-120.130
H2N
O
O
N
NC
Me
F
Ph
O
N
3ad
19F NMR (376 MHz, DMSO-d6)
ppm(t1)

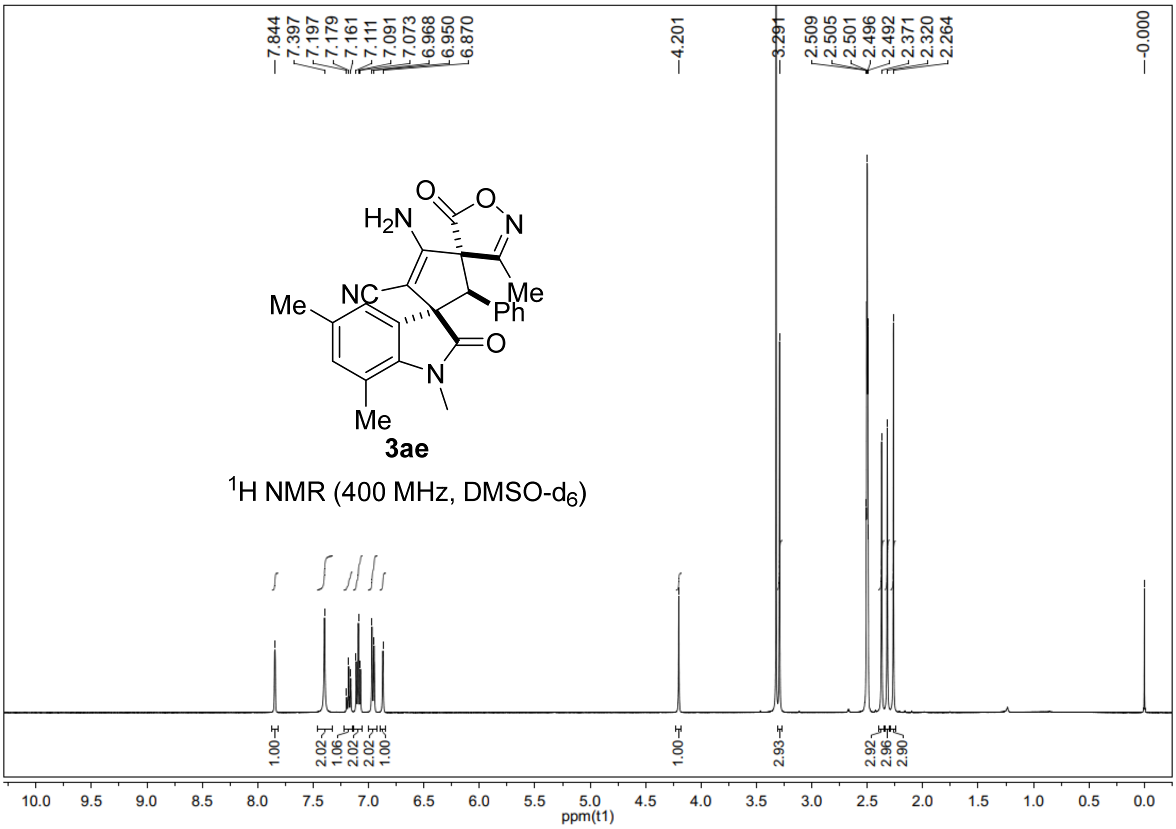
3ae
^{1}H NMR (400 MHz, DMSO-d6)
ppm(t1)

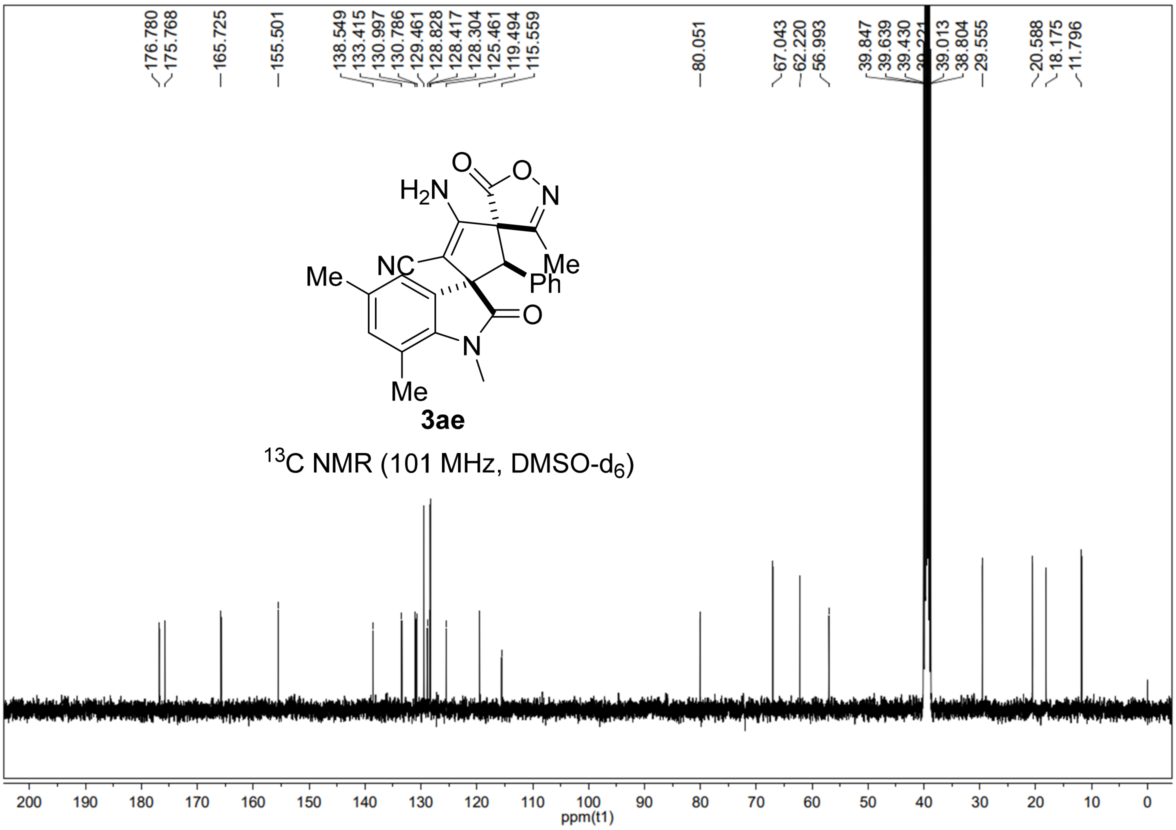
3ae
^{13}C NMR (101 MHz, DMSO-d6)
ppm(t1)

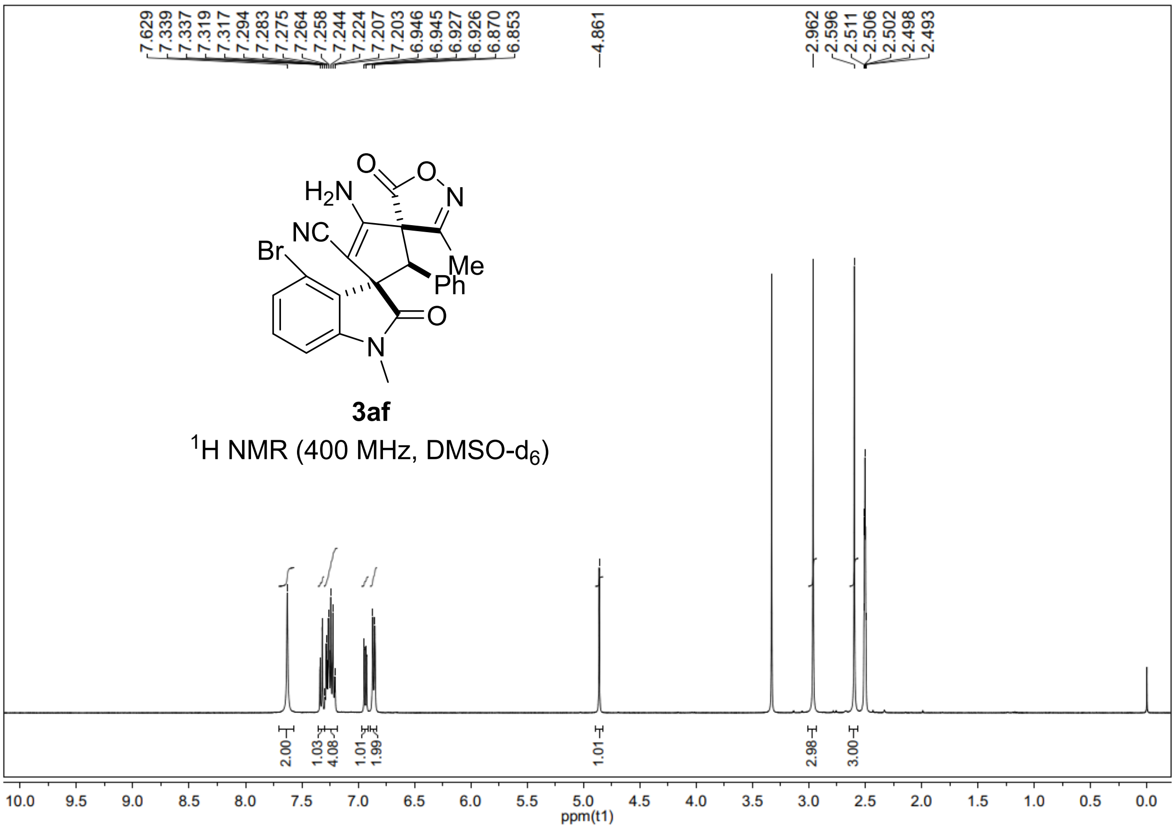
7.629
7.339
7.337
7.319
7.317
7.294
7.283
7.275
7.264
7.258
7.244
7.224
7.207
7.203
6.946
6.945
6.927
6.926
6.870
6.853
4.861
2.962
2.596
2.511
2.506
2.502
2.498
2.493
H2N
NC
Br
O
O
N
Me
Ph
O
N
3af
1H NMR (400 MHz, DMSO-d6)
2.00
1.03
4.08
1.01
1.99
1.01
2.98
3.00
10.0
9.5
9.0
8.5
8.0
7.5
7.0
6.5
6.0
5.5
5.0
4.5
4.0
3.5
3.0
2.5
2.0
1.5
1.0
0.5
0.0
ppm(t1)

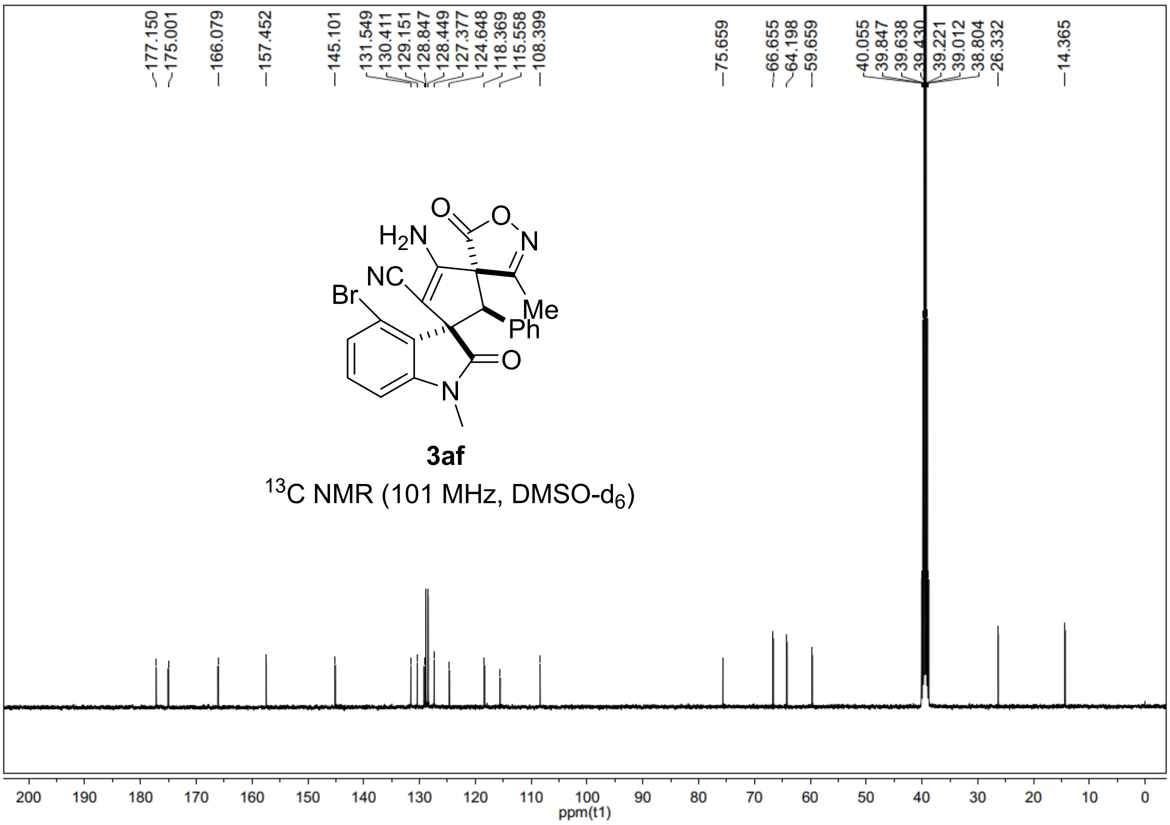
177.150
175.001
166.079
157.452
145.101
131.549
130.411
129.151
128.847
128.449
127.377
124.648
118.369
115.558
108.399
75.659
66.655
64.198
59.659
40.055
39.847
39.638
39.430
39.221
39.012
38.804
26.332
14.365
H2N
NC
Br
O
O
N
Me
Ph
O
N
3af
13C NMR (101 MHz, DMSO-d6)
200
190
180
170
160
150
140
130
120
110
100
90
80
70
60
50
40
30
20
10
0
ppm(t1)

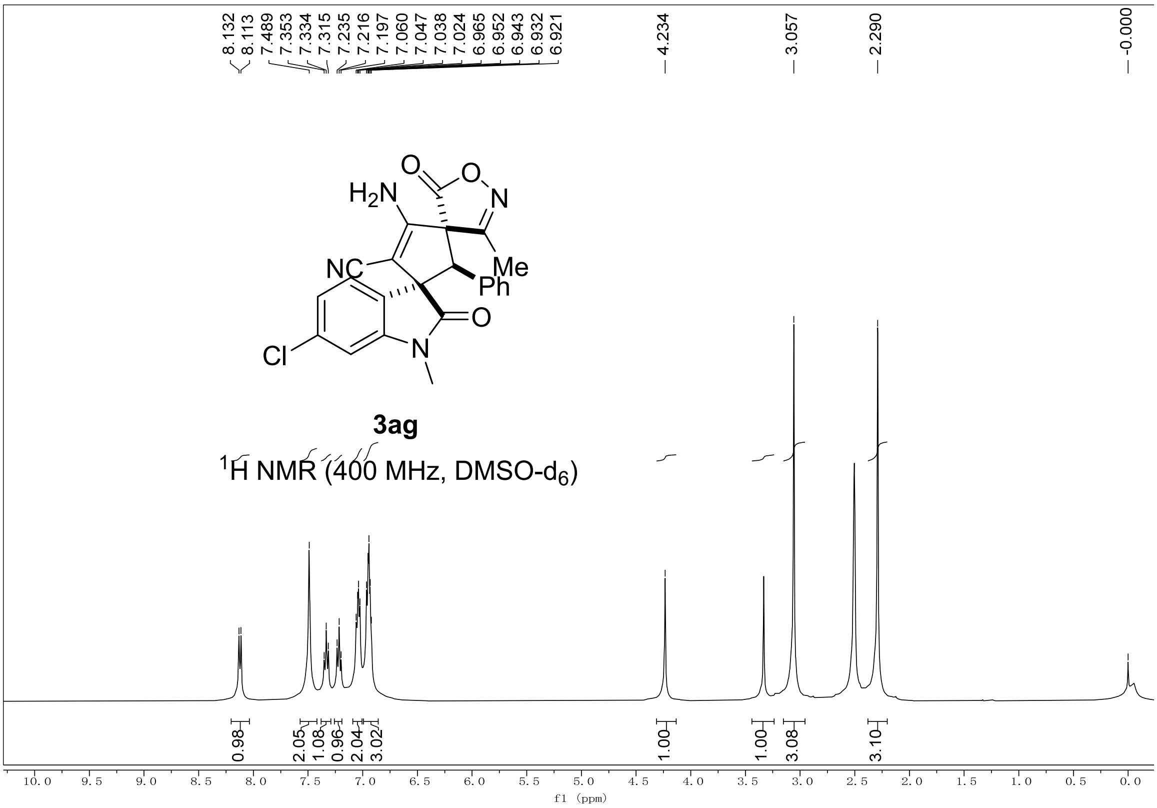
8.132
8.113
7.489
7.353
7.334
7.315
7.235
7.216
7.197
7.060
7.047
7.038
7.024
6.965
6.952
6.943
6.932
6.921
4.234
3.057
2.290
-0.000
3ag
^{1}H NMR (400 MHz, DMSO-d$_6$)
0.98
2.05
1.08
0.96
2.04
3.02
1.00
1.00
3.08
3.10
f1 (ppm)

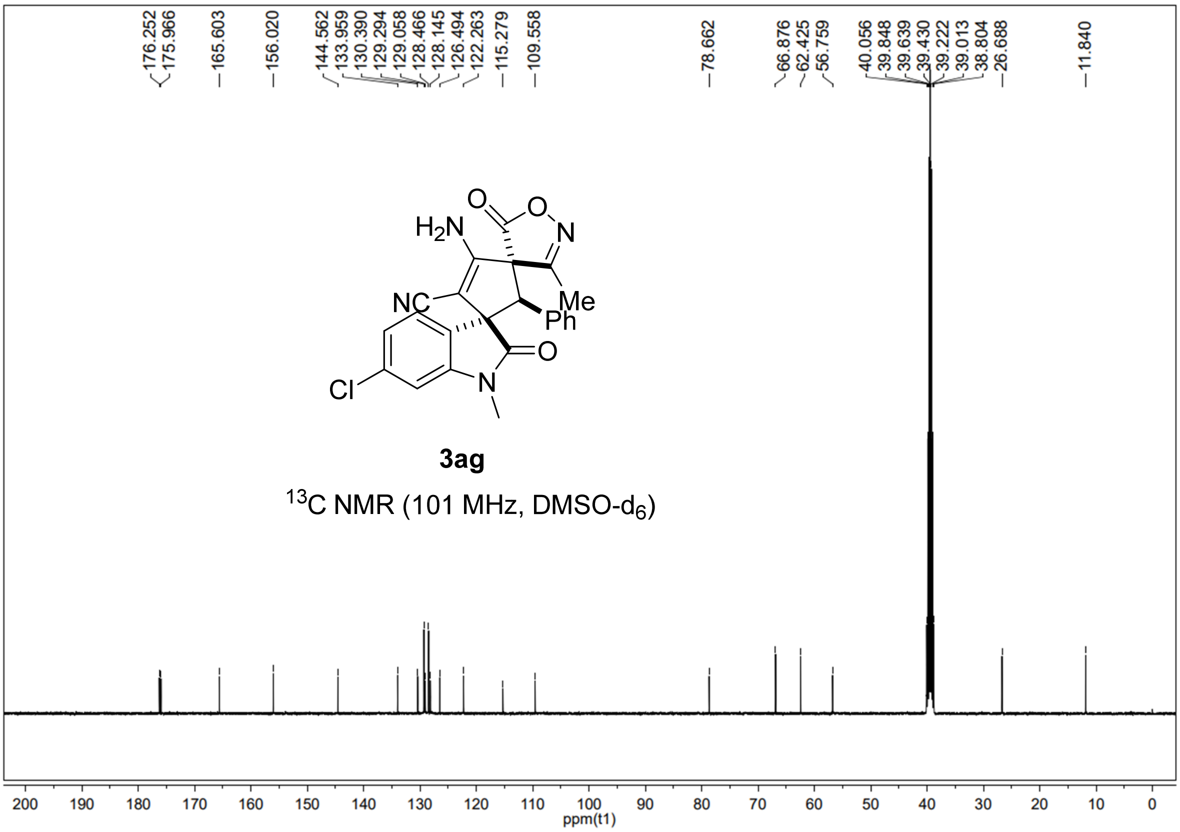
176.252
175.966
165.603
156.020
144.562
133.959
130.390
129.294
129.058
128.466
128.145
126.494
122.263
115.279
109.558
78.662
66.876
62.425
56.759
40.056
39.848
39.639
39.430
39.222
39.013
38.804
26.688
11.840
3ag
^{13}C NMR (101 MHz, DMSO-d$_6$)
ppm(t1)

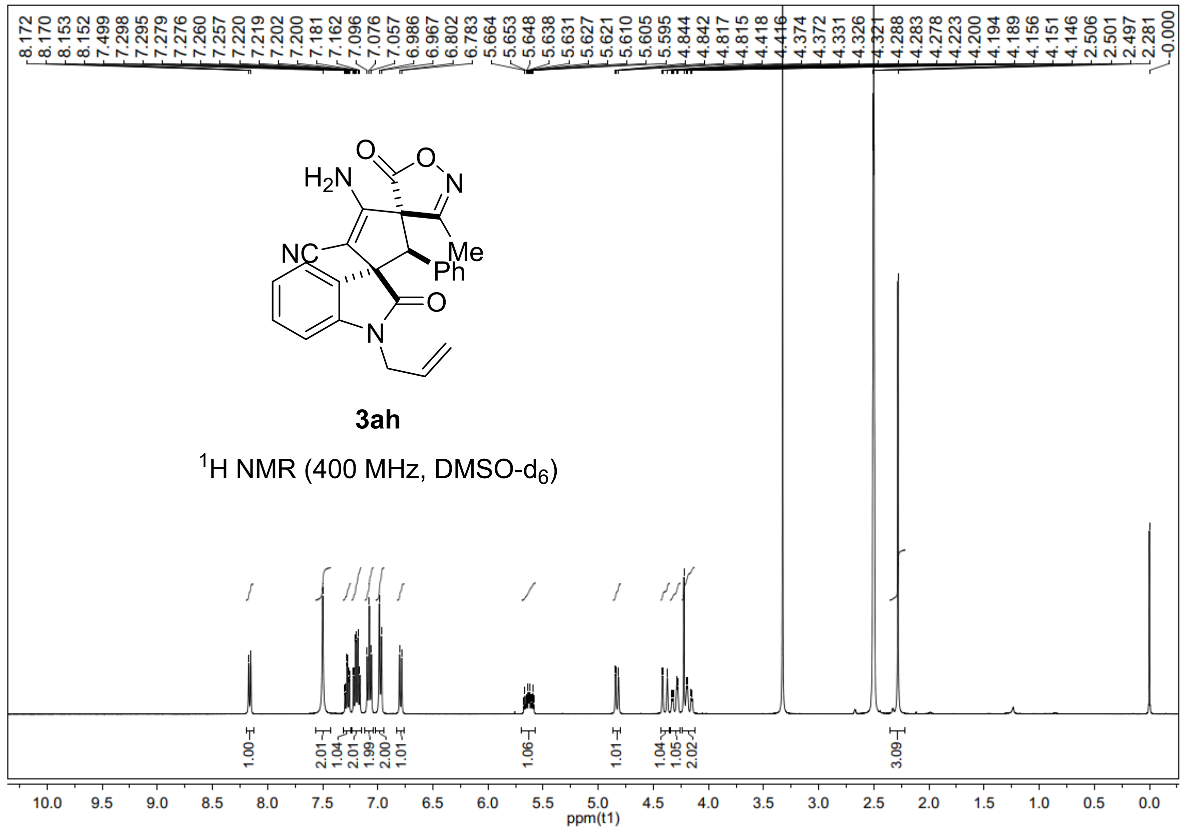
8.172 8.170 8.153 8.152 7.499 7.298 7.295 7.279 7.276 7.260 7.257 7.220 7.219 7.202 7.200 7.181 7.162 7.096 7.076 7.057 6.986 6.967 6.802 6.783 5.664 5.653 5.648 5.638 5.631 5.627 5.621 5.610 5.605 5.595 4.844 4.842 4.817 4.815 4.418 4.416 4.374 4.372 4.331 4.326 4.321 4.288 4.283 4.278 4.223 4.200 4.194 4.189 4.156 4.151 4.146 2.506 2.501 2.497 2.281 -0.000
H2N
O
O
N
NC
Me
Ph
O
N
3ah
1H NMR (400 MHz, DMSO-d6)
1.00 2.01 1.04 2.01 1.99 2.00 1.01 1.06 1.01 1.04 1.05 2.02 3.09
ppm(t1)

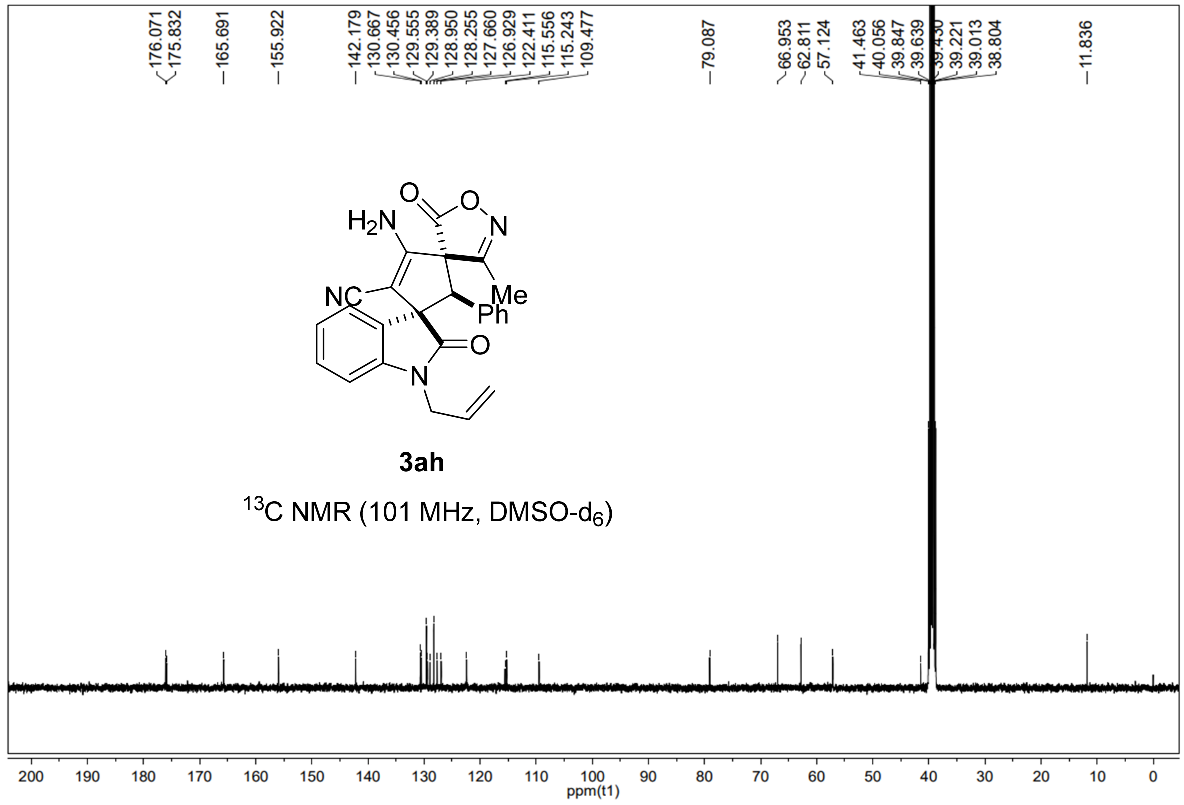
176.071 175.832 165.691 155.922 142.179 130.667 130.456 129.555 129.389 128.950 128.255 127.660 126.929 122.411 115.556 115.243 109.477 79.087 66.953 62.811 57.124 41.463 40.056 39.847 39.639 39.430 39.221 39.013 38.804 11.836
H2N
O
O
N
NC
Me
Ph
O
N
3ah
13C NMR (101 MHz, DMSO-d6)
ppm(t1)

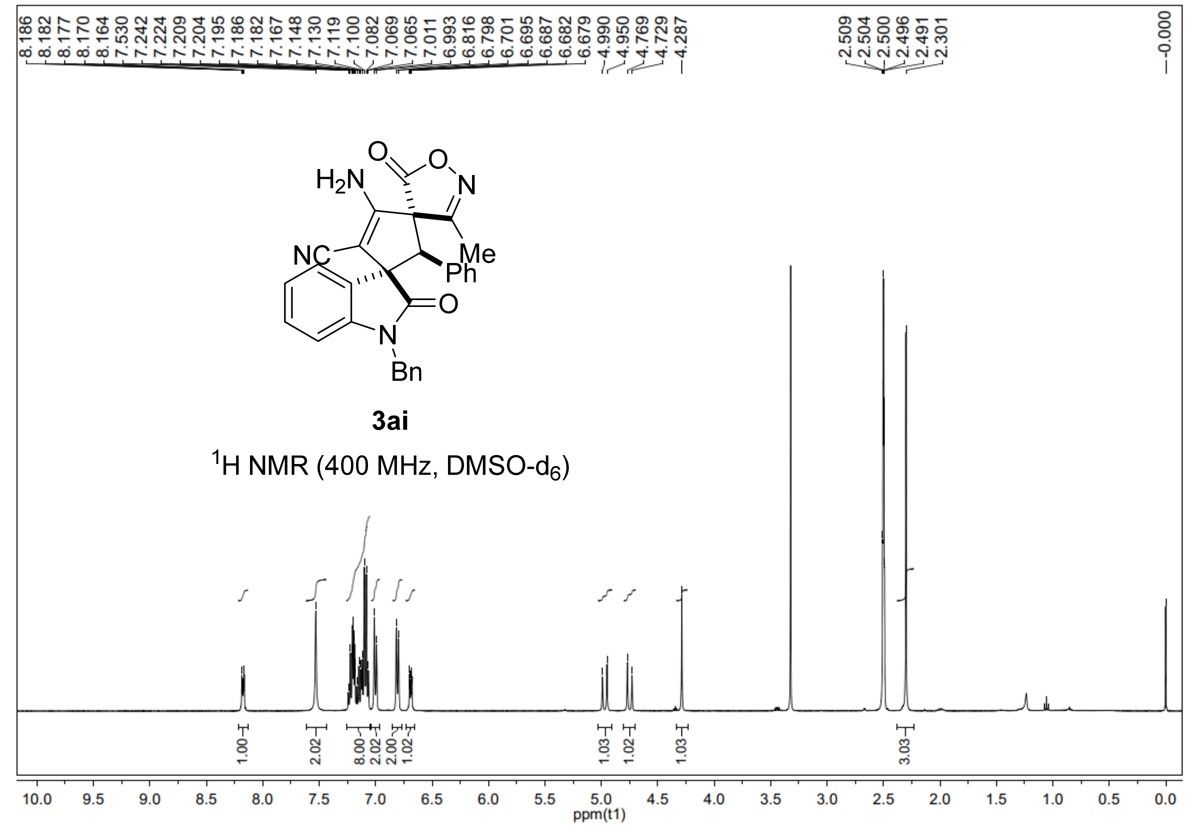
8.186 8.182 8.177 8.170 8.164 7.530 7.242 7.224 7.209 7.204 7.195 7.186 7.182 7.167 7.148 7.130 7.119 7.100 7.082 7.069 7.065 7.011 6.993 6.816 6.798 6.701 6.695 6.687 6.682 6.679
4.990 4.950 4.769 4.729 4.287
2.509 2.504 2.500 2.496 2.491 2.301
-0.000
H2N
O
O
N
NC
Me
Ph
O
N
Bn
3ai
1H NMR (400 MHz, DMSO-d6)
1.00 2.02 8.00 2.02 2.00 1.02 1.03 1.02 1.03 3.03
10.0 9.5 9.0 8.5 8.0 7.5 7.0 6.5 6.0 5.5 5.0 4.5 4.0 3.5 3.0 2.5 2.0 1.5 1.0 0.5 0.0
ppm(t1)

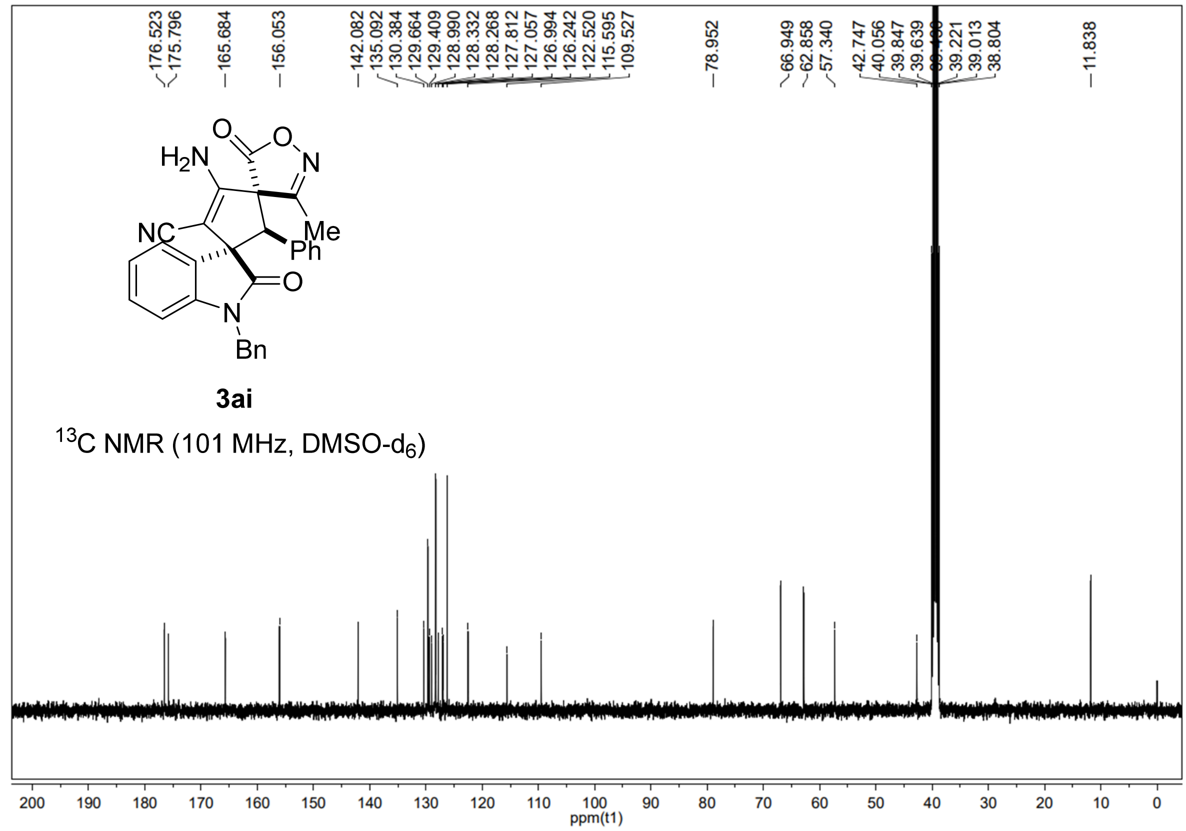
176.523 175.796 165.684 156.053 142.082 135.092 130.384 129.664 129.409 128.990 128.332 128.268 127.812 127.057 126.994 126.242 122.520 115.595 109.527 78.952 66.949 62.858 57.340 42.747 40.056 39.847 39.639 39.221 39.013 38.804 11.838
H2N
O
O
N
NC
Me
Ph
O
N
Bn
3ai
13C NMR (101 MHz, DMSO-d6)
200 190 180 170 160 150 140 130 120 110 100 90 80 70 60 50 40 30 20 10 0
ppm(t1)

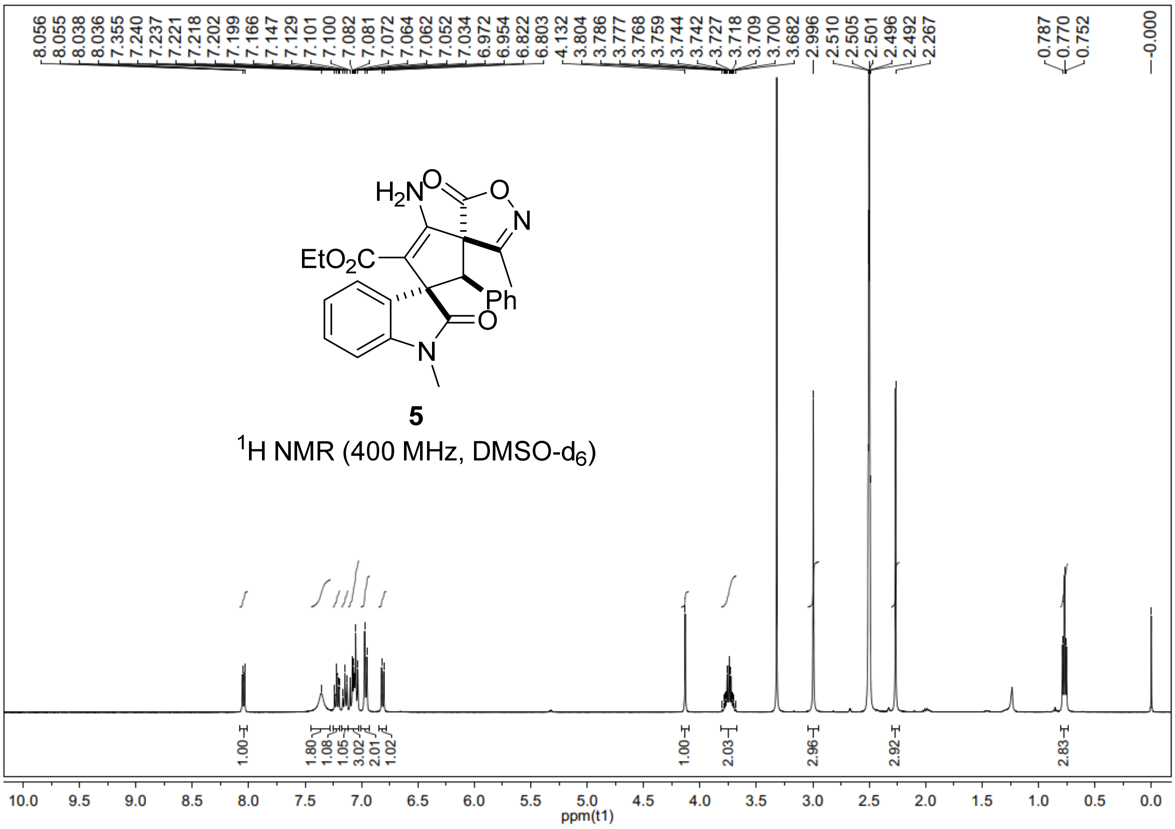
8.056 8.055 8.038 8.036 7.355 7.240 7.237 7.221 7.218 7.202 7.199 7.166 7.147 7.129 7.101 7.100 7.082 7.081 7.072 7.064 7.062 7.052 7.034 6.972 6.954 6.822 6.803 4.132 3.804 3.786 3.777 3.768 3.759 3.744 3.742 3.727 3.718 3.709 3.700 3.682 2.996 2.510 2.505 2.501 2.496 2.492 2.267 0.787 0.770 0.752 -0.000
H2N
O
O
N
EtO2C
Ph
O
N
5
1H NMR (400 MHz, DMSO-d6)
1.00 1.80 1.08 1.05 3.02 2.01 1.02 1.00 2.03 2.96 2.92 2.83
10.0 9.5 9.0 8.5 8.0 7.5 7.0 6.5 6.0 5.5 5.0 4.5 4.0 3.5 3.0 2.5 2.0 1.5 1.0 0.5 0.0
ppm(t1)

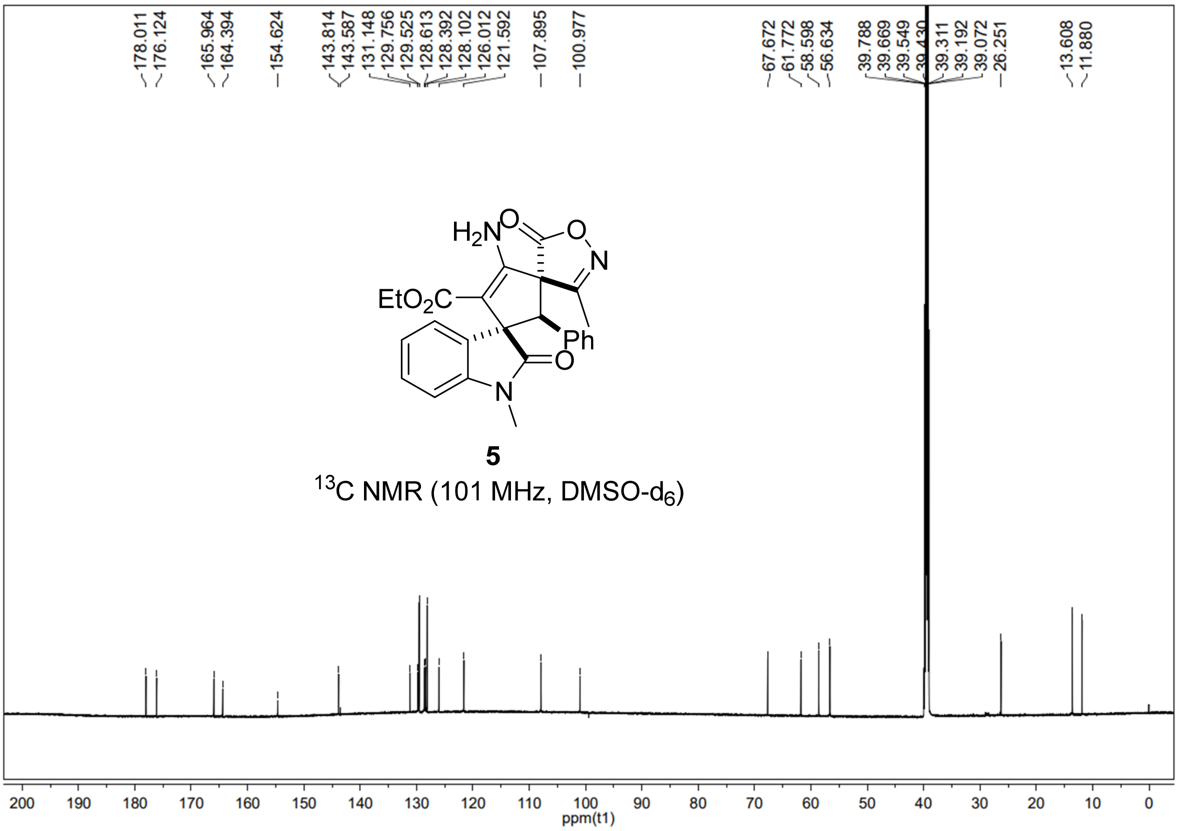
178.011 176.124 165.964 164.394 154.624 143.814 143.587 131.148 129.756 129.525 128.613 128.392 128.102 126.012 121.592 107.895 100.977 67.672 61.772 58.598 56.634 39.788 39.669 39.549 39.430 39.311 39.192 39.072 26.251 13.608 11.880
H2N
O
O
N
EtO2C
Ph
O
N
5
13C NMR (101 MHz, DMSO-d6)
200 190 180 170 160 150 140 130 120 110 100 90 80 70 60 50 40 30 20 10 0
ppm(t1)
